# A Randomized Controlled Trial of Adjunctive Family Therapy and Treatment as Usual Following Inpatient Treatment for Anorexia Nervosa Adolescents

**DOI:** 10.1371/journal.pone.0028249

**Published:** 2012-01-04

**Authors:** Nathalie Godart, Sylvie Berthoz, Florence Curt, Fabienne Perdereau, Zoé Rein, Jenny Wallier, Anne-Sophie Horreard, Irène Kaganski, Réjane Lucet, Frédéric Atger, Maurice Corcos, Jacques Fermanian, Bruno Falissard, Martine Flament, Ivan Eisler, Philippe Jeammet

**Affiliations:** 1 Department of Adolescents and Young Adults Psychiatry, Institut Mutualiste Montsouris, Paris, France; 2 National Institute for Health and Medical Research (Inserm), Unit UMR-S0669, Paris, France; 3 Paris-Sud and Paris Descartes Universities, Paris, France; 4 King′s College London, Institute of Psychiatry, London, United Kingdom; 5 Department of Biostatistics, Necker Hospital, Paris, France; 6 Institute of Mental Health Research, University of Ottawa, Royal Ottawa Hospital, Ottawa, Canada; The University of Queensland, Australia

## Abstract

Research on treatments in anorexia nervosa (AN) is scarce. Although most of the therapeutic programs used in ‘real world practice’ in AN treatment resort to multidisciplinary approaches, they have rarely been evaluated.

**Objective:**

To compare two multidimensional post-hospitalization outpatients treatment programs for adolescents with severe AN: Treatment as Usual (TAU) versus this treatment plus family therapy (TAU+FT).

**Method:**

Sixty female AN adolescents, aged 13 to 19 years, were included in a randomized parallel controlled trial conducted from 1999 to 2002 for the recruitment, and until 2004 for the 18 months follow-up. Allocation to one of the two treatment groups (30 in each arm) was randomised. The TAU program included sessions for the patient alone as well as sessions with a psychiatrist for the patient and her parents. The TAU+FT program was identical to the usual one but also included family therapy sessions targeting intra-familial dynamics, but not eating disorder symptoms. The main Outcome Measure was the Morgan and Russell outcome category (Good or Intermediate versus Poor outcome). Secondary outcome indicators included AN symptoms or their consequences (eating symptoms, body mass index, amenorrhea, number of hospitalizations in the course of follow-up, social adjustment). The evaluators, but not participants, were blind to randomization.

**Results:**

At 18 months follow-up, we found a significant group effect for the Morgan and Russell outcome category in favor of the program with family therapy (Intention-to-treat: TAU+FT :12/30 (40%); TAU : 5/29 (17.2%) p = 0.05; Per Protocol analysis: respectively 12/26 (46.2%); 4/27 (14.8%), p = 0.01). Similar group effects were observed in terms of achievement of a healthy weight (i.e., BMI≥10^th^ percentile) and menstrual status.

**Conclusions:**

Adding family therapy sessions, focusing on intra-familial dynamics rather than eating symptomatology, to a multidimensional program improves treatment effectiveness in girls with severe AN.

**Trial Registration:**

Controlled-trials.com ISRCTN71142875

## Introduction

Anorexia Nervosa (AN) is a severe illness affecting 0.5 to 1% of adolescent females [1]–[3]. AN has been associated with social disability [4], [5], psychological comorbidity [6], [7], physical complications [8], [9], as well as a 10% mortality rate [10]. There is evidence that the prognosis may be worse in patients for whom hospitalization is required [11], [12].

The research on treatments in AN is scarce. Although most of the therapeutic programs used in ‘real world practice’ in AN treatment resort to multidisciplinary approaches, they have rarely been evaluated [13]. Family therapy (FT) has been reported to be the most effective treatment for AN adolescents [13]–[15]. Specifically, studies in AN adolescents have documented the impact of family interventions that directly mobilize family resources in tackling anorexic behaviours [16]–[25].

Yet these previous studies left several important questions on the impact of FT in AN unanswered. Notably, as pointed out by Fairburn [26], it is unclear whether the effectiveness of ‘family-based treatment’ (e.g., the Maudsley manualized program, London, UK [16], [27]) is a consequence of parental involvement in getting patients to eat well and maintain a healthy weight, or whether it is rather due to major changes in intra-familial relationships. Moreover, while there is increasing evidence supporting the value of FT for the acute treatment of young AN outpatients, little is known regarding its effect among inpatients. One exception is the randomized controlled trial (RCT) study by Russell et al. [16], which supports the effectiveness of FT in this severely affected population, but only a small number of participants were included (11 had individual therapy; 10 had FT). In addition, no study has compared a program involving only the patient and the parents with one involving the whole family. Hence, other studies are needed to better understand the factors accounting for treatment effectiveness of FT in severe AN cases (e.g., young AN patients needing hospitalization).

This study [28] aimed to further investigate these questions by determining whether the adjunction of FT intervention, focusing on the improvement of the intra-familial dynamics, would be associated with a better outcome than that of the usual multi-dimensional treatment program alone (which addresses eating disorder symptomatology (see Methods), and in which the parents are routinely invited to participate [29].

To do so, we designed a pragmatic RCT to evaluate a modification of our usual multidisciplinary therapeutic approach, i.e adding a relationship-focused FT to the usual treatment. As it has been shown that strict treatment trial protocols are associated with low acceptance rates [30], [31], both arms of the RCT retained the flexibility of our current therapeutic outpatient program, which is systematically adapted to each individual situation. This procedure aimed to maximize treatment compliance and minimize dropout.

### Objectives

This study [28] aimed to determine whether the adjunction of FT intervention, focusing on the improvement of the intra-familial dynamics, would be associated with a better outcome than that of the usual multidimensional treatment program alone (which addresses eating disorder symptomatology) and in which the parents are routinely invited to participate [29].

## Methods

The protocol for this trial and supporting CONSORT checklist are available as supporting information (see Checklist S1 and Protocol S1).

### Participants

#### Inclusion Criteria

13 to 21 year-old females, with a DSM-IV diagnosis of AN, aged under 19 at illness onset and with an AN duration ≤3 years at admission to the hospital, hospitalized in our inpatient unit for AN, living in the Paris metropolitan area, and who had never received FT. The patient could receive appropriate medication.

Exclusion criteria: inability to speak or read French, and/or understand the interview questions, any metabolic pathology interfering with eating or digestion (e.g., diabetes), or psychotic disorder. This criterion also concerned the patients' parents.

### Recruitment and randomization

**Figure 1:**
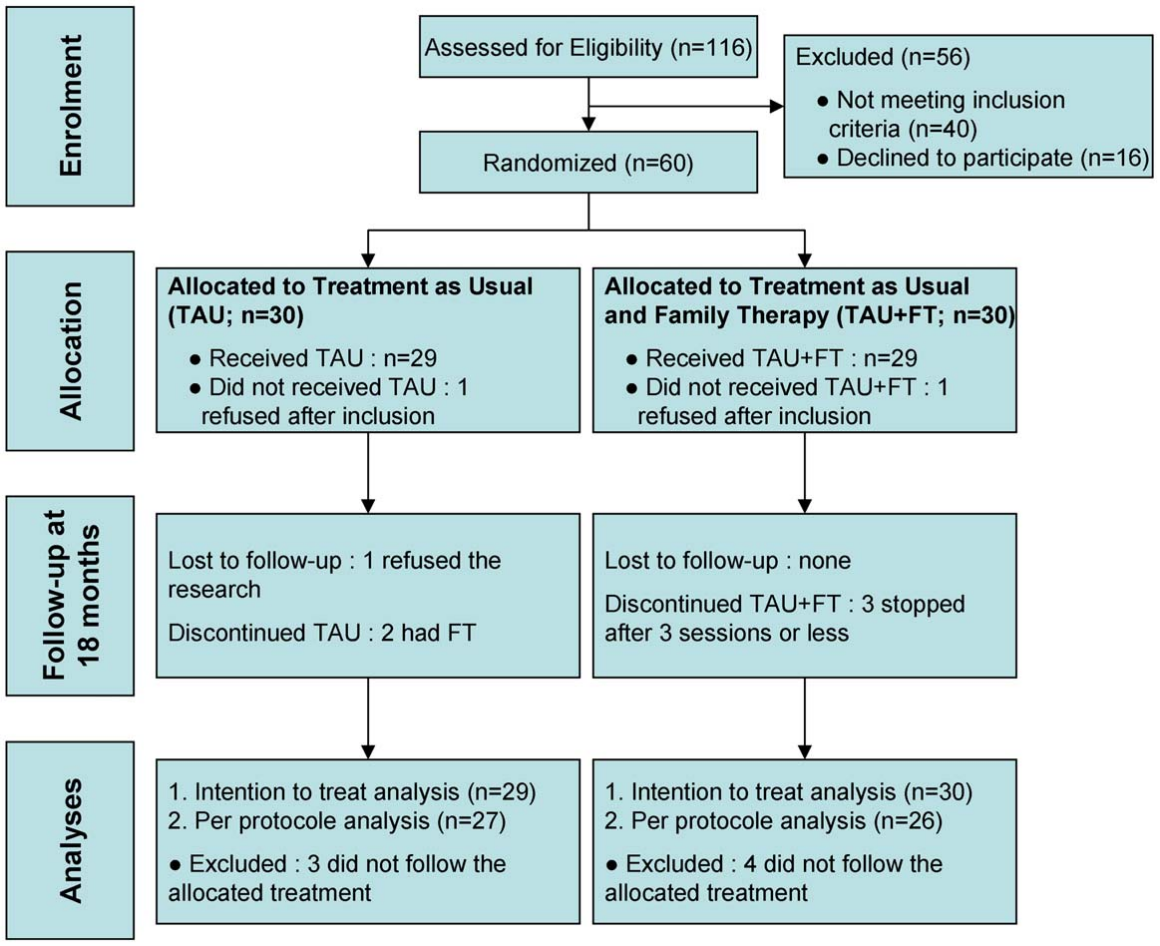
Flow chart of the randomized control trial. TAU: treatment as usual; TAU+FT: treatment as usual and family therapy.

Figure 1 illustrates participants' selection and their assignment to the two treatment groups.

This study received approval from the Ile-de-France III Ethics Committee and is in accordance with the terms of the Helsinki declaration. Participants were asked to provide informed consent after a time lapse for consideration. Written consent was completed by the patients and their parents. Prior to inclusion in the study, all participants were hospitalized in our care unit for life-threatening physical and/or mental states (including BMI below 14 and or rapid weight loss and/or compromised vital functions, severe depression, high suicide risk, chronic under-nutrition with low weight, and failure of out-patient care). Once the patient was admitted, the objectives of hospitalization were defined by means of a weight contract establishing a discharge target weight [29], [32], [33]. For each patient hospitalized between January 1999 and July 2002, a screening file sheet was completed by a psychiatrist not involved in the patient's treatment (NG or FP) but in collaboration with the patient's clinicians. Although each patient and her parents were informed of the study at admission, the inclusion and randomization occurred in the second half of their hospital stay (i.e., half way towards their target weight), at the time when the post-hospitalization program is defined. With respect to the delay in reporting these results, it was mainly due to a lack of funding.

Out of the 116 patients for whom eligibility was assessed during the recruitment period, 40 did not meet our selection criteria (10 males; 14 for whom illness onset occurred at age 19 or older; or an illness duration >3 years, 3 had a parent with schizophrenia; 5 were living outside the Paris area; 8 had had FT previously). Out of the 76 eligible participants, 16 refused (21%) to participate. Among these, 8 refused randomization, 2 refused any form of assessment, 6 refused follow-up. The patients and parents who refused to participate did not differ from those included with regard to socio-demographic variables, or clinical status on entry and at discharge (data not shown).

Allocation to one of the two parallel treatment groups (30 in each) was performed using the SPSS randomisation program (FC). The two groups were randomized by blocks of thirty. The result was issued to participants in a sealed envelope at inclusion by the psychiatrist in charge of signing the consent form (FP or NG).Theses psychiatrists enrolled the patients and assigned them to the intervention group. The first FT appointment was scheduled immediately after randomization.

### Treatment

#### Treatment as usual (TAU)

Consisted in ambulatory care initiated before hospital discharge and was tailored according to the mental and physical state of the patient [29], [32], [33]. It included individual consultations, regular interviews involving the parents, and, if required, individual psychotherapy with another therapist.

At each appointment, the psychiatrist conducted clinical investigation of the patient's mental state, eating habits, medical condition, and psychosocial environment. In addition, the psychiatrist provided support, coordinated services (e.g., general practitioner, psychotherapist, dietician or nutrionist, social worker, and school), prescribed medication as necessary, and offered parental support and guidance regarding conflicts they had with their daughter. Parents were advised to be supportive but to leave decisions about food to the adolescent and to discuss the difficulties they observed not directly with their daughter during or after the meal, but at the time of the consultations with the psychiatrist and their daughter. In addition, nutritional/dietetic advice was provided to the patients who were not gaining weight or not gaining sufficient weight.

#### Family therapy (FT)

Was designed by our team as one component of a multi-dimensional outpatient care program [28], [34]–[36]. We considered AN as a disorder resulting from multidimensional pathways [37], [38]. In interaction with premorbid personality or predispositions, the intra-familial dynamic was conceptualized as potentially influencing the occurrence and maintenance of the patient's eating problems [39].

The main aims of FT were:

1. To construct and maintain the therapeutic alliance;
2. To identify areas of individual responsibility and clarify inter-generational boundaries;
3. To promote abilities to protect, contain and provide support to the family;
4. To enable appropriate expression and management of conflict;
5. To enable the family to rediscover its own resources and strengths;
6. To restore a collective sense of family identity;
7. To develop the patient's autonomy.

Accordingly, FT focused not only on issues in the here-and-now, but also on unresolved issues from the past, as well as on expectations of how these might impact the future. Sessions focused on the familial dynamic as a whole and did not address eating behaviors directly (which were addressed by the reference psychiatrist). The sessions included the patient, her parents, and her siblings if they were over the age of 6 and living in the home. They lasted approximately 1 h30 mn and took place every three or four weeks. To optimize outcome, the frequency of sessions was flexible [20]. FT was proposed for a period of 18 months.

#### Treatment integrity

Two co-therapists (IK,RL) jointly conducted the entire FT, so that the approach was consistent. The psychiatrist and psychologist involved in the study had more than four years of experience in the outpatient care of AN adolescents. In addition, the family therapists attended weekly meetings with the reference psychiatrists and other practitioners, during which forthcoming situations in the families were discussed. To ensure that the therapies were running satisfactorily, further meetings were programmed every two to three months with the research team members and the family therapists. In this way, the consistency of follow-up was verified.

### Assessment and Procedure

The following evaluations were conducted at the time of randomization and 18 months later (see [28] for further details) in the Institut Mutualiste Montsouris department of psychiatry (Paris France):

the Mini-Neuropsychiatric Interview (MINI, [40]);
the Global Outcome Assessment Scale (GOAS, [41], [42]);
the Eating Disorder Inventory (EDI, [43], [44]);
the Weissman's Social Adjustment Scale (SAS, [45], [46]).

In addition, BMI (kg/m^2^), menstrual status, contraceptive use and the number of hospitalizations in the course of follow-up were recorded.

Regarding weight status assessment, in view of the patients' age, we considered the Ideal Body Weight (IBW) (which is classically defined as the average body weight of the general population over 15 years of age) to be a less relevant index than BMI percentiles. Hence, to take the ages of our patients into account, we referred to the INSERM (French National Institute for Health and Medical Research) weight curves for the French female population [47], in which a BMI<10^th^ percentile indexes AN [48]. We defined the outcome categories as follows [16], [49]: 1) Good outcome : weight >10^th^ BMI percentile and regular menstruation; 2) Intermediate outcome: >10th BMI percentile but amenorrhea (i.e., the absence of menstruation for at least the past three months); 3) Poor outcome: weight <10th BMI percentile and/or presence of bulimic symptoms.

At 18 months, in case of contraceptive use, subjects with a BMI<10^th^ percentile were conservatively rated as presenting amenorrhea (8 participants).

To ensure comparability we used the methodology recommended by Russell et al. [16], and pooled the Good and Intermediate outcome categories.

The evaluators, but not participants, nor the therapists, were blind to randomization. The interviews were conducted by one psychologist and two psychiatrists previously trained in the administration of the above-mentioned instruments. Each patient, her parents and siblings were assessed individually at inclusion and at 6, 12 and 18 months after inclusion. The patients and their parents were evaluated by two different interviewers (see [28]).

Drop-outs were restricted in number by systematic postal or telephone recall by the research team, the psychiatrist, or the family therapists. One patient refused follow-up (Figure 1).

Immediately after each evaluation, a dual check procedure was applied to the files obtained, enabling verification of the exhaustiveness of questionnaire completion. Thereafter, the evaluators conducted qualitative checks with the family's clinicians and, when required, the patients' medical charts. Outcome category scoring was conducted by the patient's interviewer, and then discussed with and validated by the coordinating psychiatrist (NG).

### Data Analysis

#### Power/Effect size calculation

Sample size estimation was calculated according to the Casagrande & Pike method [50] and based on RCT data on AN adolescent inpatients available at the time when the study was designed (i.e. in 1997) [16]. The expected proportion of Good or Intermediate outcomes was set to 90% in the TAU+FT group and 40% in the TAU group. With a type one error (2-sided) and a type two error equal to 0.05, the sample size estimates was 50. In line with the study by Russell et al., who reported 17% participants lost to follow-up [16], 10 additional participants were added. Thus a sample size of 60 participants was planned (30 in each arm; Figure 1). The recruitment procedure ended when this number was reached.

#### Evaluation criteria at 18 months after inclusion

The primary outcome criterion was the Morgan and Russell outcome category (good or intermediate outcome versus poor outcome) at 18 months.

The secondary outcome indicators were the GOAS total score, AN symptoms or their consequences (BMI, amenorrhea, EDI scores), social adjustment and the number of hospitalizations in the course of follow-up. The effect size was evaluated for qualitative variables by the odd ratio and its confidence interval as recommended by Fleiss et al [51] and for quantitative variables by Cohen's d test.

#### Between-group comparisons

Treatment groups were compared on socio-demographic and clinical characteristics at baseline and at 18 months of follow-up.

#### Completeness of follow-up data

Fifty-six participants were seen at 6 months, 49 at 12 months, and 55 at 18 months.

Five were not seen at 18 months: 2 in the TAU group and 3 in the TAU+FT group. Of these 5 participants, only one was completely lost to follow-up, 2 were seen only at 6 months, and 2 were seen for the last time at 12 months. Missing data were modeled using the Last Observation Carried Forward (LOCF) procedure, which enabled the inclusion of 59 participants (29 TAU; 30 TAU+FT).

We first realized Intention to Treat Analyses (ITTA) and then Per Protocol Analysis (PPA). For the ITTA, randomized patients who didn't receive any treatment were included in the analyses and these patients were followed up in the trial. For PPA, in line with Russell's et al. trial [16], only those who attended more than three sessions of FT were considered in the analyses. Accordingly, 53 received the treatment provided for in the protocol (Figure 1). Among the TAU+FT participants, 4/30 (13.3%) did not receive FT (≤3 FT sessions). Conversely, 2/30 of the TAU participants (6.7%) did in fact receive FT prescribed by there psychiatrist (outside the trial) due to a context of family crisis; 1 was lost to follow-up (Figure 1). Therefore, the PPA compared 27 TAU with 26 TAU+TF.

#### Analyses

The two treatment groups were compared at 18-months of follow-up with an alpha risk of 0.05 for two-sided tests. The Chi^2^ or Fisher Exact Probability tests were used for the categorical variables. Either Student t-tests or Mann-Whitney tests were used (as appropriate) for the continuous variables. Finally, we used matched series Student tests for intra-group comparisons exploring the evolution of quantitative criteria, and Mac Nemar tests for qualitative variables between inclusion and 18-months of follow-up. All tests were two-sided. Analyses were performed using SPSS 11.

## Results

### Participant characteristics

Descriptive statistics of the 60 AN participants are presented in Table 1. There were 5 AN purging subtype in the TAU+FT group and 3 in the TAU group (no group effect, p = .71). At the start of the study, all the participants were on amenorrhea and the TAU and TAU+FT groups were comparable. The mean BMI at admission clearly indicates the seriousness of their condition (i.e., much lower than the third percentile: 16.23 kg/m^2^ for 16 to 16.4 year-old [47]). Both groups had a mean BMI at discharge over the 10^th^ percentile (i.e., 17 kg/m^2^ for16.5 to 16.9 year-old [47]). A quarter of the participants had been previously hospitalized for AN: 11participants had one previous hospitalization, 1 was previously hospitalized twice, 3 had three previous hospitalizations. The two groups were comparable in terms of comorbid mood and anxiety disorders (i.e., major depressive disorder, social phobia, panic disorder, agoraphobia, obsessive compulsive disorder, post traumatic stress disorder; details available on request from the authors). Importantly, on average, the participants received 18.9 (±7.3) psychiatric consultations, including 6.5 (±4.6) parental consultations in 18 months, with no between-group differences (p = 0.20). In addition, 14 participants were treated with individual therapy (7 in each group) and received, on average, 23.4 (±23.03) sessions; there was no significant difference (p = 0.22). The TAU+FT participants attended an average of 11.8 (±5.7) FT sessions. The total number of treatment sessions (consultations, FT, individual therapy) did not differ between the two groups (TAU: 27.2±12.7; TAU+FT: 33.7±24.6; p = 0.55).

**Table 1:** Patients Characteristics at inclusion.

	All (n = 60)	TAU+FT (n = 30)	TAU (n = 30)	t tests or χ2; df	P
Age at onset of disorder: years, mean (SD)	14.8 (1.6)	14.7 (1.7)	15.0 (1.5)	−0.64; 58	.52
Age at inclusion: years, mean (SD)	16.6 (1.6)	16.4 (1.7)	16.6 (1.7)	−0.27; 58	.79
AN duration: months, mean (SD)	16.6 (6.8)	17.1 (8.3)	16.1 (5.2)	0.54; 58	.59
Minimum BMI: kg/m^2^, mean (SD)	13 (1.1)	12.9 (1.1)	13.1 (1.2)	−0.91; 59	.37
BMI at admission: kg/m^2^, mean (SD)	13.6 (1.1)	13.5 (1.0)	13.7 (1.3)	−0.87; 58	.39
BMI at inclusion: kg/m^2^, mean (SD)	16.9 (1.1)	17.0 (1.2)	16.9 (1.0)	0.36; 58	.72
BMI at discharge: kg/m^2^, mean (SD)	17.5 (1)	17.6 (1.1)	17.5 (0.9)	0.46; 58	.65
% of ABW at admission: mean (SD)	64.2 (5.5)	63.5 (5.3)	64.9 (5.7)	−0.97; 58	.33
% of ABW at inclusion: mean (SD)	83.6 (5.2)	83.9 (5.6)	83.3 (5.0)	0.93; 58	.70
% of ABW at discharge: mean (SD)	86.6 (4.9)	86.9 (5.3)	86.2 (4.5)	0.52; 58	.60
Duration of hospitalization: weeks, mean (SD)	21 (13.9)	22.4 (16.1)	19.4 (11.5)	0.82; 58	.41
GOAS: Global Score, mean (SD)	4.3(1.1)	4.3 (1.1)	4.3 (1.2)	−0.16; 58	.87
EDI: Global score, mean (SD)	60.7(35.1)	61.3 (36.2)	60.2 (34.6)	0.12; 58	.90
SAS: Global score, mean (SD)	2.6 (0.6)	2.6 (0.6)	2.6 (0.6)	−0.11; 58	.91
Previously hospitalized: No (%)	15 (25.0)	8 (26.7)	7 (23.3)	0.09; 1	.77
Drop-out (below discharge target weight): No (%)	12 (20.0)	5 (16.7)	7 (23.3)	0.42; 1	.52
Family status: Not intact, No [%]	9 [15.0]	3 [10.0]	6 [20.0]	-; 1	.47

ABW: Average body weight [59]; AN: anorexia nervosa; BMI: body mass index; EDI: Eating disorders inventory; GOAS^:^ Global Outcome Assessment Scale; SAS: Social Adjustment Scale; SD: standard deviation; TAU: treatment as usual; TAU+FT: treatment as usual and family therapy; No: number; % percentage.

### Changes in the group as a whole

Between inclusion and 18–months of follow-up, the overall sample showed significant improvement for all the parameters considered: the MR outcome score, the GOAS score, the EDI and SAS total scores, the BMI and menstrual status (Detailed results available on request from the authors).

### Primary Outcome

The proportion of patients who belong to the Good and Intermediate Outcome category was more important in the group treated with adjunctive family therapy (Table 2). In terms of odds ratio, the TAU+FT participants achieved Good or Intermediate outcome 3.2 times as often as those from the TAU group in the whole group (ITTA: p = 0.054) and 4.9 times as often as those in the restraint group (PPA: p = 0.013) (Table 2). Among the participants with a Good or Intermediate outcome (17/59), more than half (10/17) met the criteria for Good outcome.

**Table 2:** Global Outcome at 18 months.

	TAU+FT	TAU	χ2; df	p	Absolute effect size (95% CI)	Relative effect size OR (95% CI)
Good or intermediate MR outcome score ITTA: (n = 59), No/n. [%]	12/30 [40]	5/29 [17.2]	3.7;1	.054	22.8 (−0.4;42.9)	3.2 (0.9;10,)
Good or intermediate MR outcome score PPA: (n = 53) No/n.(%)	12/26 [46.2]	4/27 [14.8]	6.2;1	.013	31.3 (6.5;51.8)	4.9 (1.3;18.3)

95% CI: 95% confidence interval; MR: Morgan and Russell; ITTA: intention to treat analysis; PPA: per protocol analysis; OR: odd ratio; TAU: treatment as usual; TAU+FT: treatment as usual and family therapy.

### Secondary Outcome criteria

- The GOAS total (Table 3) and sub-scale scores (details available on request from the authors) did not differ between the two groups.

**Table 3:** Secondary Outcome (ITTA).

	TAU+FT (n = 30)	TAU (n = 29)	t or χ2; df	p	Absolute effect size (95% CI)	Effect size* (95% CI)
BMI≥10th percentile, No.[%]	16 [53.4]	8 [27.6]	4.0; 1	.044	25.8 (0.76;46.7)	3 (1.0;8.9)
Amenorrhea, No.[%]	11 [36.7]	19 [65.5]	4.9; 1	.027	28.9 (3.4;49.6)	0.3 (0.1;0.9)
GOAS Global Score, mean (SD)	7.6 (2.2)	7.1 (2.2)	.83; 57	.41	0.5	0.23 (−0.56;1.3)
EDI Total score, mean (SD)	48.2 (29.8)	47.4 (28.4)	.95; 52	.92	0.8	0.03 (−10.6;10.4)
SAS total score, mean (SD)	2.0 (0.8)	2.0 (0.8)	−.23; 48	.82	0	0 (−.29;0.29)
Psychiatric re-hospitalizations, No.[%]	12 [40.0]	16 [55.2]	1.4; 1	.24	0.15 (−10;37.5)	1.8 (0.7;5.2)
Re-hospitalisation for AN, No.[%]	10 [33.3]	14 [48.3]	1.4; 1	.24	14.9 (−9.7;37.3)	1.9(0.8;5.3)

Abbreviations: MR: Morgan and Russell; GOAS ^:^ Global Outcome Assessment Scale; ITTA: intention to treat analysis; TAU: treatment as usual; TAU+FT: treatment as usual and family therapy; SAS: Social Adjustment Scale; SD standard deviation; 95% CI: 95% confidence interval.

*Relative effect size: odd ratio for qualitative variables; Cohen's d for quantitative variables.

- The proportion of patients who achieved a healthy weight (BMI≥10^th^ percentile) and resumed menstruation was more important in the TAU+FT group (Table 3).

**-** The TAU+FT participants achieved a healthy weight about 3 times as often as those from the TAU group in the whole group (ITTA: p = 0.044) (Table 3).

**Figure 2:**
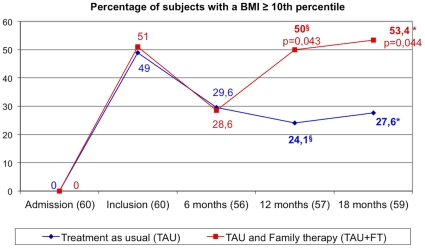
Percentages of participants with a BMI≥10^th^ percentile. TAU: treatment as usual; TAU+FT: treatment as usual and family therapy.

Figure 2 illustrates the proportion of patients with a BMI above the 10^th^ percentile at the end of the follow-up period. Overall, BMI increased significantly (n = 59: 16.9±1.09 to 17.6±2.3; t = −2.36,df = 58, p = 0.021). Nevertheless, the gap between the two treatment groups began to widen significantly at 12 months of treatment and remained at 18 months.

When each group was considered separately, only the TAU+FT group showed a significant evolution in average BMI (30 TAU+FT: 17.0±2.0 to 17.8±2.1, t = −2.11, df = 29, p = 0.044; 29 TAU: 16.9±1.0 to 17.4±2.4, t = 1.27 df = 28, p = 0.22).

- The TAU+FT group presented amenorrhea significantly less often (OR  = 0.3; p  = 0.027) than the TAU group (19/29) (Table 2).

- We observed no significant group effect for the EDI total (Table 3) and sub-scale scores (details available on request from the authors).

- Mean SAS scores (Table 3) did not differ between the two treatment groups (p>0.05).

- Overall, 28/59 (47.5%) of the patients were re-hospitalized at least once for AN or another psychiatric disorder. Although this percentage was greater in the TAU (55.17%; i.e., 16/29) than in the TAU+FT (40%; i.e., 12/30) group, the difference did not reach statistical significance (Table 3).

## Discussion

The present RCT study assessed the therapeutic effectiveness of the adjunction of family therapy (FT), focusing on the family dynamics, to the usual outpatient treatment (TAU) of severely ill AN adolescents. Our hypothesis was that, relative to TAU alone, TAU+FT would improve global outcome, AN symptoms, social adjustment and would reduce the frequency of re-hospitalization at 18 months of follow-up.

We showed that the proportion of patients who belong to the Good and Intermediate Outcome category was more important in the group treated with adjunctive family therapy (between 22.8% and 31.3%, depending on the ITTA or PPA analyses). In other terms, patients treated with adjunctive family therapy were 3 to 4.9 times more likely to belong to the Good and Intermediate Outcome category [49]. Specifically, the proportion of patients who achieved a healthy weight and resumed menstruation was more important in the group treated with adjunctive family therapy (respectively 25.8% and 28.9%). In other terms, over 3 times more AN adolescents achieved a healthy weight and resumed menstruation. However, we found no differences for subjective evaluations of eating behaviors and attitudes, social adjustment, or for relapses.

We found two main results. First, in AN adolescents, adding family therapy (including parents and siblings), with a specific focus on intra-familial dynamics (and not on eating behaviors), to an established integrative multi-disciplinary outpatient treatment, significantly improved the outcome at 18 months of follow-up. This finding suggests that a treatment targeting intra-familial dynamics has a specific effect. Our study design made it possible to rule out the hypothesis that the key ingredient for family therapy effectiveness in AN is that it places “*greater emphasis on getting patients to eat well and maintain a healthy weight*” (see [26], page S27). Moreover, our results are in line with those of Pike et al. [52] who showed that cognitive behaviour therapy in post-hospitalisation treatment of AN adult patients is significantly more effective in improving outcome and preventing relapse than nutritional counselling alone.

Second, we showed that weight and menstruation normalization occurred significantly more often in the FT group, despite the fact that these symptoms were not specifically targeted during the therapy sessions. This finding has a critical clinical implication, as long illness duration has been associated with higher mortality rates [53], and lasting denutrition and amenorrhea have been linked to severe somatic complications (such as osteopenia or osteoporosis [14]).

In the literature, only six studies in AN adolescents have compared FT to another treatment. These studies compared the contribution of FT to that of individual therapy [16], [17], [21], [22], [54], or compared two types of FT intervention [18], [19], [54], [55], or compared two FT durations [23], [24]. Overall, these studies suggest that: FT participants have a better outcome; conjoint and separated FT have similar effects; FT of six or 12 months' duration have similar effect.

Across all these studies, between 60 and 95% of patients achieved a good or intermediate outcome and continued to improve during follow-up. Here, this was the case for 46.2% of the participants treated with family therapy (versus 14.2% among the treatment as usual participants). Several factors could account for this discrepancy, such as the use of different criteria for hospitalization as well as variations in referral and recruitment procedures.

The most direct comparison is with the study by Russell et al. [16], which included adolescents with similarly low weights on admission to hospital (around 65% ABW), similar duration of illness (1.2–1.5 years) and high levels of previous treatment. Yet several arguments suggest a possible difference in illness severity between our sample and that of Russell et al. First, whereas these authors exclusively included patients who agreed to hospitalization and who completed the inpatient program, we included numerous adolescents who had refused care at the time of admission but who were hospitalized by their parents (i.e., they were minors). Second, we did not exclude participants who had not reached their target weight when they were discharged from hospital (20% of our sample). Finally, in the Russell et al. study [16], FT participants had a significantly shorter hospital stay (8.8 weeks) than those in the individual therapy group (12.1 weeks). This could be an indirect indicator of a selection bias towards participants experiencing lesser difficulties in their FT group.

With respect to the other studies in AN adolescents that compared FT to another treatment, the seriousness of the participants' condition was usually below that of our sample:

The reported weights at the time of treatment inclusion (e.g, 91% of Ideal Body Weight in the study by Robin et al. [21]) are above those of our study participants (i.e., 64.2% at admission and 83.6% at inclusion);
The participants were younger on average by 2 to 3 years and had shorter illness duration (i.e., inclusion criteria included an illness duration <1 year [22], [23]) than in our study;
Past hospitalization was also less common (e.g., half at most had been previously hospitalized in the study by Eisler et al. [18], versus 100% in our sample) (but see also [16]).

Hence, the question whether FT effectiveness is predicted by severity of illness should be addressed in future studies.

With respect to the proportion of favorable outcomes, the finding of a relatively small difference (although significant) between our two treatment groups might also be partially explained by the fact that, unlike the study by Russell et al. [16], the parents here were involved in both types of treatment with a substantial benefit. Indeed, similar small differences in favor of FT have been observed in studies which, like the present one, compared two modes of care involving the parents in some way [20], [21]. Future studies comparing different FT approaches should help to address this question.

In the present study, contradicting our hypothesis, adjunctive family therapy had no significant effect on the reduction of relapses relative to the usual treatment (respectively 33.3% and 48.3%). Nevertheless 46.7% of the overall sample required re-hospitalization in the course of follow-up (18 months). Although this is higher than the 10% re-admission rates reported by the Maudsley group [16], [17], it is similar to those of other follow-up studies of AN adolescent outpatients (e.g., 25–30% of re-admissions after a first admission and 50–75% after subsequent admissions [56]–[58]).

The main strength of the present study, which gives us confidence in the findings, is that it was sufficiently powered, with low participant dropout at follow-up. Nevertheless, one limitation of this research was that we did not use a FT manual. However, though not formally set out in a manual, our method has been described in medical publications, journals, and training sessions [34]. Furthermore, since only two family therapists from our team jointly conducted the sessions, we believe that this limitation had little impact [35]. It could also be argued that another limitation is that the FT group received 12 additional sessions compared to the other group. This is not in fact the case, as the total number of treatment sessions of all kinds did not differ between the two groups.

To our knowledge, this is the first randomized controlled trial designed to compare two multidimensional post-hospitalization outpatient treatment programs for adolescents with AN, which differed solely with regard to the presence of family therapy centered on intra-familial dynamics of the whole family.

FT was effective, although the family therapists did not directly address eating problems, weight, and the evolution of the illness. It yielded better progress at 18 months of follow-up in terms of global outcome, weight and menstruation status than the standard treatment. The additional burden of treatment in terms of time for the family, and in terms of cost, is moderate (on average, 12 sessions of 1 h30).

Although the family therapy and therapeutic program modalities set out in our protocol are somewhat different from those described by the teams that have published their investigations on this topic, they were found effective here. Different team cultures, varying departmental backgrounds, and different healthcare systems have generated many techniques to treat anorexia nervosa. These techniques, although different, may be equally effective and not necessarily better or worse one than another. What is essential, in our view, is that there is a need to assess the contribution of each technique, its prerequisites or its limitations. Subsequent to this, it would be possible in the future to compare these different FT techniques, with regard to their effectiveness, but above all to determine the best indications for each. For example, one might consider which patients would benefit more from focusing on eating attitudes and weight during family therapy and which would not.

The evaluation of these techniques and the determination of their particular indications might make it possible to avoid situations where patients ‘sink’ into prolonged periods of malnutrition despite treatment. These difficult-to-treat cases remain all too numerous, and progress in this domain would make it possible to offer patients, at the beginning of treatment, optimum individually tailored care.

## Supporting Information

Protocol S1 **Trial Protocol.**
(DOC)

Checklist S1 **CONSORT Checklist.**
(DOC)
