# Supplementary material for: A Randomized Controlled Trial of Adjunctive Family Therapy and Treatment as Usual Following Inpatient Treatment for Anorexia Nervosa Adolescents
Source: PLoS One. 2012 Jan 4;7(1):e28249. doi: 10.1371/journal.pone.0028249 (PMC3251571; doi:10.1371/journal.pone.0028249)
Supplement: Protocol S1 — Trial Protocol. (DOC) [file pone.0028249.s001.doc]

EXPOSE DU PROJET DE RECHERCHE:

**Apports de la thérapie familiale au traitement des patientes anorexiques mentales**

**Rédaction du projet :**

Nathalie Godart [[1]](#footnote-2)1 , Martine Flament [[2]](#footnote-3)2 , Maurice Corcos[[3]](#footnote-4)3 , Frédéric Atger[[4]](#footnote-5)4, Irène Kaganski[[5]](#footnote-6)5 , Réjane Lucet[[6]](#footnote-7)6 , Philippe Jeammet [[7]](#footnote-8)7 .

**Conseil statistique :**

Fernando Perez-Diaz[[8]](#footnote-9)8

**Conseil a l’élaboration et suivi du projet :**

Christopher Dare[[9]](#footnote-10)9 , Yvan Eisler[[10]](#footnote-11)10

TABLE DES MATIERES

1. Résumé du protocole

2. Introduction et justification de la recherche

2.1. Pourquoi ce projet

2.2. Pourquoi une étude préliminaire : les problèmes pratiques pour la réalisation d’une telle étude

2.2.1. Nombre de sujets nécessaires à la mise en évidence des hypothèses

2.2.2. Durée de l’étude

2.2.3. Conclusion

2.3. Pourquoi une demande de financement avant la fin de l’étude préliminaire

2.4. Pourquoi une étude unicentrique

2.5. Résultats attendus et leurs conséquences (perspectives concrètes et utilité pour les malades et pour la pratique médicale)

2.5.1. Résultats attendus

2.5.2. Conséquences : Perspectives concrètes et utilité pour les malades et pour la pratique médicale

3. Données de la littérature et pré-requis

3.1. Données de la littérature :

3.1.1. Thérapie familiale et anorexie mentale: revue de la littérature

3.1.2. Fonctionnement familial

3.1.3. Symptomatologie et facteurs de pronostic

3.1.4. Caractéristiques familiales et facteurs de pronostic.

3.1.5. Adaptation sociale et facteurs de pronostic.

3.1.6. Alexithymie et maladies à composante psychosomatique

4. Objectifs de la recherche (hypothèses testées, objectif primaire, objectifs secondaires)

4.1. Hypothèses testées

4.1.1. Première hypothèse

4.1.2. Deuxième hypothèse

4.2. Objectif du projet

4.2.1. Objectif primaire

4.2.2. Objectifs secondaires

5. plan expérimental :

5.1. Choix du plan expérimental et justification

5.1.1. Plan de l'étude

5.1.2. Randomisation

5.2. Critères de sélection des personnes, mode de recrutement

5.2.1. Critères d'inclusion

5.2.2. Critères de non inclusion

5.2.3. Mode de recrutement et Bilan d'inclusion

5.2.4. Non respect du protocole

5.3. Nombre prévu de personnes et justification

5.4. Durée de participation de chaque personne ayant accepté de participer à la recherche

6. Schéma et conduite de la recherche (voir schéma ci-dessous)

6.1. Suivi des patients :

6.1.1. chronologie et contenu des visites

6.1.2. actes

6.1.3. lieu de réalisation des examens

6.2. Durée totale prévisionnelle de la recherche

7. Procédures évaluées

7.1. Traitement en hospitalier

7.2. Traitement étudié : la thérapie familiale

7.2.1. Modalités pratiques

7.2.2. Généralités

7.2.3. Axes de traitement

7.3. Traitements ambulatoire après hospitalisation

7.3.1. Pratique classique

7.3.2. Adaptations pour l’étude

8. Critères d’évaluation : variables mesurées et méthodologie

8.1. Critères de jugement

8.1.1. Critères de jugement principaux

8.1.2. Critères de jugement annexes

8.1.3. Actes réalisés

8.2. Méthodes et techniques utilisées : instruments composants le questionnaire et l’autoquestionnaire

8.2.1. Diagnostics

8.2.2. Symptômes et attitudes alimentaires :

8.2.3. Les facteurs psycho-sociaux :

8.2.4. La psychopathologie générale : le SCL-90-R

8.2.5. La symptomatologie anxio-dépressive

8.2.6. Antécédents de maladies psychosomatiques et d’alexithymie

8.2.7. Les relations familiales :

9. 9- Gestion des données et statistique :

9.1. 9.1 Stratégie d’analyse des données collectées

9.2. Justification des tests statistiques

9.3. Responsable de l’analyse des données (déclaration CNIL si nécessaire) et logiciels de travail

10. Effets graves éventuels :

11. Aspects légaux et éthiques

11.1.1. Note d’information aux sujets et formulaire de consentement adaptés à chaque groupe si nécessaire

12. Analyse et chiffrage des surcoûts hospitaliers avec leurs modalités de financement

12.1. personnel (médical, non médical)

12.1.1. Surcoût hospitalier lié à l’inclusion

12.1.2. Surcoût hospitalier lié à l’évaluation par une psychologue

12.1.3. Surcoût hospitalier lié à la thérapie familiale

12.2. dépenses générales (missions, fournitures de bureau ...)

12.2.1. Surcoût lié à l’impression des dossiers.

12.2.2. Surcoût lié au traitement des données.

12.2.3. Surcoût lié à la saisie des données.

12.2.4. Surcoût lié à l’ analyse des données.

12.2.5. Prévision des dépenses de Mission

12.2.6. Surcoût lié à l’acquisition du logiciel Exner

12.3. .Conclusion

13. Références bibliographiques

14. Annexes

14.1. Annexe 1

14.2. Annexe 2

# Résumé du protocole

***objectifs*** Nous projetons de réaliser une **étude clinique, randomisée, prospective**, évaluant **l’efficacité de l’adjonction d’une psychothérapie familiale au programme de soins classiquement proposé dans l’anorexie mentale**, à travers plusieurs dimensions que sont : la nature et gravité des symptômes alimentaires, la symptomatologie anxio-dépressive, l’adaptation sociale, les relations familiales.

***Justificatif*** La **fréquence de l'anorexie mentale et la gravité de son évolution**, malgré les soins proposés, nous ont conduit à nous interroger sur **l'adaptation possible des modalités thérapeutiques** à proposer à ces sujets. **L'expérience dont nous disposons** d'une contribution de l'approche familiale aux soins de l'anorexie mentale (Jeammet et al, 1980 ; Kaganski et al, 1989) nous ont conduit à poser l'hypothèse de l'intérêt d'une **thérapie familiale** systématique au cours de l'anorexie mentale, en plus de l'approche classique. **Ce type d’étude standardisé n’a, à notre connaissance, jamais été réalisée**. Après une étude préliminaire afin de calculer l’effectif de l’échantillon à inclure dans une telle étude, de déterminer sa durée, d’évaluer le taux d’acceptation des familles et leur compliance au traitement, de tester notre protocole d’évaluation, nous souhaitons mettre en place **une étude de suivi et d’évaluation de cette cohorte de patients pendant un minimum initial de deux ans** ( durée de traitement souhaitable pour une pathologie de durée d’évolution longue).

***Etat de la question*** Si les articles de discussions cliniques sur l’efficacité de la thérapie familiale dans le traitement de l’anorexie mentale sont très nombreux, on constate qu’il existe **peu d'études contrôlées** évaluant leur **efficacité**. Nous avons retrouvé trois équipes impliquées dans ces recherches d’évaluation des thérapies familiales entre 1966 et 1998, ayant réalisé en tout six études. Ces études montrent une meilleure efficacité de la thérapie familiale comparée à la thérapie individuelle chez les sujets ayant débuté leur anorexie avant 18 ans et depuis moins de trois ans.

***Hypothèses*** La **thérapie familiale** adjointe aux soins classiquement proposés après une hospitalisation pour **anorexie mentale** permet d’en **favoriser la guérison**.

***Méthode*** Nous pensons recruter deux groupes de minimum **trente familles** pour les suivre deux ans (voir calendrier). Les sujets et les parents seront évalués individuellement, à l’entrée dans l’étude tous les six mois, lors d’entretiens menés par un membre de l’équipe de recherche, grâce au questionnaire précédemment défini. **L’évaluation** des patients et de leur famille sera réalisée à partir d’un **autoquestionnaire et d’un hétéroquestionnaire.**

L’hétéroquestionnaire comprendra : le Mini International Neuropsychiatric Interview (MINI), la section " angoisse de séparation" de la SADS-LA (R) (version révisée de la "Schedule for Affective Disorders and Schizophrénia modified for the study of anxiety disorders"), des éléments cliniques, l'échelle de Morgan et Russell sur les troubles alimentaires, l’échelle d’adaptation sociale de GRONINGEN: "Groningen Social Disability Schedule seconde version", l'échelle de dépression d’Hamilton, l'échelle de phobie sociale de Liebowietz, l'échelle d'Obsession Compulsion de Yale-Brown (Yale-Brown Obsessive-Compulsive Scale, Y-BOCS), une échelle de fonctionnement familial . L’autoquestionnaire comprendra : le questionnaire de dépression de Beck, le questionnaire de dépression Blatt, le questionnaire sur l’alimentation EDI (Eating Disorders Inventory) de Garner, le questionnaire d’adaptation sociale (Social adjutsment scale self report : SAS-SR), le questionnaire de psychopathologie générale SCL-90-R de Derogatis, un questionnaire de fonctionnement familial le FACES et un questionnaire sur l’attachement le PBI.

***Analyses*** Nous donnerons une **description détaillée de nos échantillons**. Les sujets ayant refusés de participer et les non compliants seront comparés au reste de l’échantillon. La **comparaison de l’efficacité** des deux protocoles thérapeutiques proposés se fera en comparant les deux groupes de sujets définis sur les variables considérées

# Introduction et justification de la recherche

## Pourquoi ce projet

L'**anorexie mentale** est une **pathologie fréquente** parmi les adolescents. Elle concerne, selon les critères du DSM-III-R (American Psychiatric Association, 1989), approximativement 1% des jeunes filles en fin d’adolescence. (American Psychiatric Association, 1994 ; Flament et al 1995). Ce trouble est beaucoup moins fréquent parmi les garçons qui ne représentent que 1/10 des patients soignés pour anorexie mentale.

L’anorexie mentale, se caractérise **par la gravité de son pronostic**, qui la classe au premier rang des pathologies psychiatriques mettant en jeu le pronostic vital (Wipple et Manning, 1978): 50% de ces patients ont un devenir qualifié "d'intermédiaire ou de mauvais" (Steinhausen et al, 1991 ).

Les **taux de mortalité** les plus souvent relatés dans la littérature internationale se situent entre **4% et 10%** ; plus le suivi est long plus la mortalité augmente. Elle atteint **20% à 20 ans de suivi**. Ces décès sont la conséquence directe de l'anorexie pour 50% d'entre eux, les autres étant liés à un suicide (24%), à des pathologies pulmonaires (6%), à des accidents (6%), et à des causes inconnues (14%) (Herzog 1988). Une étude réalisée dans notre service (Jeammet et al 1991), rapporte une mortalité de 7%. Cette mortalité est considérable pour des sujets de cet âge puisqu'elle est, selon Patton et al (1988), six fois supérieure à celle observée dans une population générale comparable.

**L'âge moyen de début** de l'anorexie mentale se situe à l'adolescence vers 17 ans (Hardy et al, 1989 ; Willi et al, 1990 ; DSM-IV, 1994). A cette période de leur vie ces patients vivent encore pour la plus part chez leur parents, ou ont quitté depuis peu le foyer familial. Les liens affectifs entre le sujet et ses parents sont intenses. On conçoit aisément le rôle central que joue alors les parents à cette époque charnière de la vie du sujet. L’adolescence conduit le sujet de l'enfance à l'état adulte, de la dépendance aux parents à l'autonomisation et à la sexualisation des individus. Elle est caractérisée par des transformations physiques, biologiques, psychiques et sociales. C'est aussi un moment de reviviscence des conflits infantiles et d'une reprise des conflits d'identification (Jeammet, 1989a).

C'est bien souvent à la faveur de la perspective de la **séparation d’avec les parents**, ou de la mise en acte de cette séparation, que se **déclarent les symptômes anorexiques liés** à la fragilité de l'individu. En effet, l'adolescence est un moment révélateur de la qualité des intériorisations établies pendant l'enfance, et parfois, comme dans le cas de l'anorexique, de leur échec partiel, comme en témoigne la permanence d'une dépendance de ces adolescents à l'égard de leur entourage (Jeammet 1991b). En effet, si l'enfant n'a pu intérioriser les relations avec son entourage, il restera dépendant physiquement de celui-ci, générant alors une situation conflictuelle qui lui est très difficile à gérer. Pour ces sujets, recevoir quelque chose des personnes dont ils sont dépendants c'est se soumettre à elles et s'abandonner jusqu'à menacer leur identité. Ces adolescents perçoivent alors celui qui peut satisfaire ces besoins comme détenteur d'un pouvoir sur eux (Jeammet, 1989). Si la plupart des auteurs ne soutiennent plus une relation de causalité entre les interactions familiales et la survenue de l'anorexie mentale, ils y voient le plus souvent un élément favorisant la pérennisation du trouble (Jeammet et al, 1980)**.** L'hypothèse la plus consensuelle actuellement développée fait de **l'anorexie mentale un trouble du comportement alimentaire d'origine multifactorielle** (Garner, 1993, Halmi, 1996). Comme le résume Garner (1993) l'anorexie mentale résulterait de facteurs prédisposants, de facteurs déclenchants et de facteurs renforçants. Ce qu'il schématise ainsi:

**Facteurs Facteurs Facteurs**

**prédisposant précipitant pérennisants**

individu

(biologie, psychologie) insatisfaction régime pour symptômes

concernant augmenter de dénutrition

famille le poids estime de soi et et réaction

et la silhouette maîtrise de soi des autres

culture

**Anorexie mentale en tant que trouble multifactoriel**

**(D'après Garner The Lancet, 1993)**

**C'est pour cela que les soins dispensés aux sujets anorexiques par de nombreuses équipes (Whipple et Manning, 1978 ; Gatard, 1988 ;Yager, 1988, 1994; Haller,1992 ; Halmi et al, 1996,), dont la nôtre, sont multidisciplinaires**. Ils comportent une approche psychothérapeutique individuelle, des soins somatiques, une assistance pour les problèmes sociaux et d’insertion scolaire. En plus des soins individuels des patients, une prise en charge parentale systématique au travers d'entretiens familiaux et de thérapie de groupe de parent s’est mise en place.

En revanche, à la sortie de l'hospitalisation, si une prise en charge bifocale avec un psychiatre consultant et un psychothérapeute est le plus souvent proposée, le travail avec les parents est moins systématiquement préconisé. Les parents sont reçus de façon espacée par le consultant, à leur demande ou à celle du psychiatre, sans que l'on puisse parler de thérapie ; s'ils le souhaitent le groupe de parents leur reste ouvert, mais ils l'utilisent rarement. Dans quelques cas, bien souvent sur des arguments de gravité concernant le patient et/ou concernant les relations familiales, on prescrit une thérapie familiale dont les objectifs sont d'aider le sujet et sa famille à aménager leurs relations, tant à l’intérieur qu’à l’extérieur de la famille, tout en renonçant au symptôme alimentaire (Kaganski et al, 1989).

**La fréquence de l'anorexie mentale et la gravité de son évolution malgré les soins proposés, nous ont conduit à nous interroger sur l'adaptation possible des modalités thérapeutiques à proposer à ces sujets, afin d'améliorer leur devenir**. L'anorexie mentale est une affection dont l'origine psychique est sous-entendue dans son nom lui même. Actuellement tous les cliniciens soignant ces patients, quelques soient leurs orientations théoriques, s'accordent pour dire que la base des traitements qui doivent être dispensés est de nature psychothérapique (Mynor-Wallis, 1989), l'efficacité en ayant été démontrée (Crisp 1991, Gowers 1994). **Les thérapies sont reconnues plus efficaces que les médicaments dans le traitement de l’anorexie mentale** (Yager, 1988). Les pratiques tendent à associer plusieurs techniques de psychothérapie telle que la thérapie individuelle et familiale (Wipple et Manning, 1978 ; Dare et Eisler, 1997). Cette approche doit être bien entendu couplée à d'autres soins indispensables à type de renutrition, de soins corporels, de médicaments si nécessaire (antidépresseurs, anxiolytiques, etc...).

Dès 1975, Morgan et Russell ont montré que la perturbation des relations familiales était un facteur corrélé avec un devenir de mauvaise qualité (Morgan et Russell 1975), sans que cela puisse être associé à une structure familiale anormale ou à des cas de pathologie mentale dans la famille. **L'expérience d'une contribution de l'approche familiale aux soins de l'anorexie mentale au travers du groupe de parents lors de l'hospitalisation (Jeammet et al, 1980), et l'expérience clinique de la thérapie familiale acquise par certains d'entre nous (Kaganski et al, 1989) au cours de la prise en charge ambulatoire de certaines anorexies mentales, nous ont conduit à poser l'hypothèse de l'intérêt d'une thérapie familiale systématique au cours de l'anorexie mentale, en plus de l'approche classique.**

Si de **nombreuses voix s’accordent pour souligner l’importance d’une approche familiale dans les troubles du comportement alimentaire,** la plupart se basent sur leur expérience clinique subjective pour l'affirmer. **Les études menées sur le sujet sont peu nombreuses et pas toujours très rigoureuses**. Russell souligne en 1993, que les traitements prescrits dans l’anorexie mentale ont rarement fait l’objet d’étude clinique standardisée. **Les pratiques traditionnelles ont rarement été évaluées**. De même Crip (1991) souligne que les **études d’efficacité sur le traitement de l’anorexie mentale sont rares** et n’ont jamais été conduites à long terme. Celles qui existent concernent le plus souvent des essais pharmacologiques ou des essais sur les thérapies comportementales.

La **nécessité de réaliser de nouvelles études avec des méthodologies plus solides portant sur la thérapie familiale**, plus particulièrement chez les patients anorexiques, est signalée par de nombreux auteurs (Diamond et al, 1996). En effet ils soulignent qu’il est important de réaliser ces études afin d’une part, de répliquer les résultats des quelques études antérieures (tels que ceux de l’étude de Russell et al, 1987), et d’autre part, d’utiliser les connaissances en psychopathologie et les procédures de recherche pour développer conjointement des modèles de traitement à partir de théories testées permettant des résultats plus robustes.

Enfin nous avons été particulièrement sensibles aux études de Russell et al (1987), car leurs conclusions favorables à la thérapie familiale concernent une population de patientes très similaire à celle majoritairement traitée dans notre centre (80%, voir étude de faisabilité annexe 1).

C’est pourquoi il nous semble **important de confirmer l’intérêt de la thérapie familiale dans l’anorexie mentale que nous avons observé dans notre pratique par une étude standardisée**. **Ce type d’étude standardisé n’a à notre connaissance jamais été réalisée en France**. De plus, si quelques équipes internationales ont traité de l’intérêt de la thérapie familiale dans l’anorexie mentale, les questions ont été posées différemment : la plupart comparaient l’intérêt de la thérapie familiale à d’autres traitements dont la thérapie individuelle ou d’autres types de soins, opposant ces prises en charge, à notre sens complémentaires. Ceci semble dépasser à l’heure actuelle, compte tenu de l’hypothèse d'une étiopathogénie multifactorielle de l’anorexie mentale. Une approche multidisciplinaire telle que nous la pratiquons est la seule envisageable, opposer thérapie familiale et thérapie individuelle ne nous semble pas pertinent. **Certes cet abord, antérieurement utilisé, permet de poser une question simplifiant de façon artificielle le problème ; en pratique aucun de nos patients n’est suivi exclusivement par l’une ou l'autre de ces méthodes. Dans un soucis constant de perfectionnement de notre pratique, nous nous interrogeons à la lumière des résultats des études antérieures sur l’intérêt de l’association d’une thérapie familiale à notre approche classique, après une hospitalisation pour anorexie mentale.**

## Pourquoi une étude préliminaire : les problèmes pratiques pour la réalisation d’une telle étude

### Nombre de sujets nécessaires à la mise en évidence des hypothèses

Le **nombre minimal de sujets nécessaire** à une étude dépend pour chaque critère de jugement défini de la force de l'effet attendu, de la variance et des risques d’erreur alpha et bêta.

Compte-tenu de la méthodologie des études réalisées antérieurement (voir revue de la littérature ci-dessous) leur résultats ne peuvent être extrapolés à notre étude pour estimer le nombre de sujets nécessaires. L’effet du traitement étudier mesurable par une différence attendue **n’est pas à ce jour connue, la littérature ne peut nous la fournir**. Nous devons donc réaliser une **étude préliminaire qui doit nous permettre d’observer la différence** afin de déterminer la taille de l’échantillon final utile à notre étude. **Cela déterminera la durée de l’étude compte tenu des autres paramètres que sont le recrutement (connu), et les taux d’acceptation et de compliance qui restent tous deux à déterminer.**

### Durée de l’étude

Pour estimer la durée de l’étude que nous projetons, nous avons fait une estimation de la faisabilité en terme de recrutement **(Voir annexe 1)**.

Nous avons arbitrairement choisi de faire une simulation pour un effectif dont la taille a été déterminée en tenant compte des impératifs méthodologiques ( nombre de variables, différence à mettre en évidence et effectifs des études précédentes les plus sérieuses) soit une soixantaine de familles au minimum. Or nous avons conclu avec cette simulation qu’il faudrait un recrutement de plus de deux ans, voire même trois ans pour atteindre cet effectif. La thérapie familiale est un traitement qui porte sur une durée de 18 mois en moyenne . En conséquence, la durée totale d’une telle étude sera donc de 4 ans et demi ans : trois ans de recrutement et 18 mois de traitement. Un tel engagement a des implications en terme de motivation des équipes mais aussi d’investissement structurel autour du projet (temps des thérapeutes familiaux, exigences imposées par la standardisation du suivi, etc .....). De plus, si nous avons, à partir de critères d’inclusion et d’exclusion, estimé un recrutement, certaines variables inconnues (telle l’acceptation des familles, leur compliance au traitement) ne peuvent être estimées. Ces éléments peuvent mettre en péril la réalisation d’une telle étude. Enfin, d’autres facteurs imprévus, peuvent apparaître et rendre caduques nos estimations.

### Conclusion

**C’est pourquoi nous avons souhaité réaliser une étude préliminaire**. Elle nous permettra, outre la mise en évidence des points précédemment soulignés (calculer l’effectif de l’échantillon à inclure dans une telle étude, déterminer sa durée, évaluer l’acceptation des familles et leur compliance au traitement), de tester notre protocole d’évaluation. Enfin l’obtention de résultats, même partiels, permettra une mobilisation et une implication plus facile de l’équipe. Cette étude préliminaire a obtenu un financement par le CRC-AP-HP 1997 et débutera aux environs du 1er avril 1998.

## Pourquoi une demande de financement avant la fin de l’étude préliminaire

**L’étude préliminaire** débutera aux environs du 1er avril 1998, elle porte sur un **recrutement de douze mois soit trente familles** et propose **un bilan à six mois** pour évaluer les paramètres précédemment définis (calculer l’effectif de l’échantillon à inclure dans une telle étude, déterminer sa durée, évaluer l’acceptation des familles et leur compliance au traitement), soit une **durée totale de dix-huit mois**. Elle nous permettra de préciser ces éléments et **d’affiner la méthodologie de notre projet définitif** exposé ci-dessous en détail. Elle comporte, outre le recrutement de trente familles, le bilan de la thérapeutique proposée à six mois. (Voir plan ci dessous)

**Patientes anorexiques**

**Hospitalisées**

**Age début ≤ 18 ans**

**Durée évolution ≤ 3 ans**

**Proposition de participation**

**à l’étude à la sortie d’hospitalisation**

**acceptation**

**Refus**

**Randomisation**

**1ère évaluation à la sortie 1ère évaluation à la sortie**

**Traitement Traitement Classique Classique + Thérapie Familiale**

**de traitement**

**Réévaluation Réévaluation**

**Plan de l’Etude préliminaire**

**sur l’évaluation de l’efficacité de la thérapie familiale**

**dans l’anorexie mentale**

**Le projet définitif qui justifie cette demande de financement** , propose outre le plan de l’étude préliminaire **un bilan de cette thérapeutique tous les six mois et ce jusqu’à dix huit mois** mais cette fois pour **soixante familles** (chiffre à confirmer par l’étude préliminaire). En **conséquence si l’on fait débuter l’étude définitive en continuité avec cette étude préliminaire la moitié de l’effectif serait déjà recruté** et ne manquerait pour cette partie de l’échantillon que douze mois de suivi.

De plus, même si nous n’avions pas les moyens de mettre en place l’étude définitive à l’issue cette étude préliminaire les thérapies familiales initiées pour cette étude, ainsi que la prise en charge globale des patientes anorexiques et de leur famille inclues dans ce protocoles préliminaire ne pourraient bien entendu pas être suspendus puisque qu’on sait que les soins de l’anorexie mentale sont des soins au long court (dix huit mois minimum pour une thérapie familiale) et l’on se priverait d’élément d’observation important.

**Compte tenu de la mobilisation importante de patients, de cliniciens et de chercheurs pour cette étude préliminaire il nous semble important de récolter des moyens de porter l’étude définitive à son terme en faisant fructifier les résultats de l’étude préliminaire.**

Or pour cela nous aurons **besoins de moyens** **à compter du sixième mois de l’étude préliminaire, soit en octobre 1998**. En effet, les premiers patients recrutés au début étude préliminaire arriverons alors au **terme de l’étude préliminaire** couverte par le financement du CRC AP-HP qui couvre les six premiers mois d’étude pour chaque patients.

Aussi souhaiterions nous alors **poursuivre la recherche entreprise sur dix huit mois durée classique de la thérapie familiale** (Voir revue de la littérature)

## Pourquoi une étude unicentrique

Plusieurs arguments plaident en faveur du choix d’un centre unique pour cette étude. Bien entendu nous avons tout intérêt à recruter l'échantillon le plus large possible, ce qui serait facilité par un recrutement multicentrique. **Cependant l’anorexie mentale bien que fréquente dans la population adolescente, est rarement traitée en grand nombre dans les services**. En effet ces patientes sont accueillies et traitées dans des services nombreux et de disciplines variées telles l’endocrinologie, la nutrition, la pédiatrie, la psychiatrie adulte, la psychiatrie infanto-juvénile, la médecine interne, la médecine générale etc... Aussi plusieurs difficultés nous sont apparues, toutes liées à un souci d’homogénéisation des modalités thérapeutiques pour réduire le « bruit de fond »:

1. Le plus souvent la file active de ces services dépasse rarement quelques cas par an. Dans un soucis d’homogénéité et de standardisation de l’échantillon il nous a semblé difficile de faire appel à de nombreux services, ce qui rendrait le recrutement très hétérogène.
2. Quand la file active des services considérés est importante, les populations recrutées sont bien souvent très différentes de celle prise en charge dans notre service (âge de début, durée de l’évolution, âge des sujets). Il n’existe pas à notre sens de service équivalent au notre dans la prise en charge de patients anorexiques en terme d’effectif dans la tranche d’âge qui nous concerne en France (hospitalisation de 13 à 23 ans).
3. Les services provinciaux pouvant le mieux correspondre à ces particularités n’ont pas tout à fait la même pratique de suivi. Les programmes thérapeutiques sont variables d’un centre à l’autre ce qui introduirait un biais de différence inter-centres non négligeable, gênant la mise en évidence d’une différence entre les groupes.
4. Il nous semble important que le traitement étudié puisse être le plus standardisé possible, ce qui est facilité par le choix d’une équipe unique avec une thérapeutique définie.

Néanmoins nous collaborons avec l’unité INSERM U 302, l’unité CNRS URA 1957 et l’équipe anglaise du Maudsley.

## Résultats attendus et leurs conséquences (perspectives concrètes et utilité pour les malades et pour la pratique médicale)

### Résultats attendus

Les **résultats** que nous attendons de cette étude préliminaire sont de plusieurs ordres.

1. Nous obtiendrons une **mesure de l’effet du programme de soins avec thérapie familiale** comparé au programme sans thérapie familiale.
2. Grâce à cette différence observée, nous pourrons **calculer l’effectif nécessaire à notre projet définitif** d’étude sur l’efficacité de la thérapie familiale.
3. Nous pourrons **tester notre méthodologie,** en testant d’une part le plan de l’étude et en testant les questionnaires. Ceci nous permettra éventuellement **d’effectuer les modifications nécessaires pour l’étude définitive.**
4. L’étude préliminaire nous permettra d’apprécier **la faisabilité de ce projet avant de nous engager dans un projet long (plusieurs années)**. En effet, elle nous permettra d’apprécier, outre l’effectif à recruter et l’adéquation de la méthodologie à notre projet, **le taux d’acceptation des patients et des familles à ce type d’étude et la compliance des patients** et des familles au traitement proposé. La détermination de l’effectif à recruter, du taux d’acceptation et de la compliance au traitement, nous permettront **d’évaluer la durée approximative d’une telle étude et sa faisabilité en termes de coût, de durée, et d’acceptation par les familles.**

### Conséquences : Perspectives concrètes et utilité pour les malades et pour la pratique médicale

#### Pour les malades

Grâce à cette étude préliminaire, nous souhaitons **obtenir les données nécessaires à la réalisation de notre projet d’étude standardisée et randomisée** d’évaluation des thérapeutiques dans l’anorexie mentale sur de plus larges effectifs.

Nous pensons pouvoir **mettre en évidence que la thérapie familiale, adjointe aux soins classiquement proposés après une hospitalisation pour anorexie mentale, permet d’en favoriser l’évolution**.

Nous pensons pouvoir montrer que :

- cette efficacité se traduit sur le plan clinique par **une amélioration plus rapide de la symptomatologie alimentaire des patients** et que cette amélioration serait stable au cours du temps.
- cette **amélioration se manifeste en dehors des symptômes alimentaires sur la psychopathologie générale,** **sur l’anxiété et sur la dépression** de ces patients.
- **l’adaptation psycho-sociale de ces patientes** sera beaucoup **plus rapidement améliorée par l’adjonction de la thérapie familiale**. Cette amélioration dans le domaine psycho-social serait observable aussi bien dans le domaine familial que dans le domaine extra familial.
- cette thérapie familiale pourra contribuer **à l’amélioration du fonctionnement familial.**

Cette thérapie familiale pourra donc bénéficier non seulement aux patientes présentant une anorexie mentale, mais pourrait aussi en diminuer les conséquences au sein de la famille.

#### Pour la pratique médicale

Ce projet est, à notre connaissance**, le premier projet d’évaluation standardisée et randomisée de l’efficacité d’une thérapeutique dans l’anorexie mentale**. Nous souhaitons mettre en évidence l’intérêt de la thérapie familiale dans la prise en charge de l’anorexie mentale, en tant que complément thérapeutique à un programme de soins classique. Cet **abord est original**, puisqu’il prend en compte les thérapeutiques classiquement appliquées à l’anorexie mentale, dont on sait qu’elles sont efficaces, et tente de mettre en évidence que celles-ci sont perfectibles. Pour cela nous souhaitons **nous appuyer sur des** données objectives obtenues par une étude standardisée.

**L’anorexie mentale est une pathologie lourde et ce à plusieurs titres** puisqu’elle met en péril :

- **l’équilibre psychologique individuel** des patientes, en favorisant, par la dénutrition, les symptômes dépressifs et anxieux
- **l’état somatique**, **en mettant en jeu le pronostic vital de jeunes individus**,et en hypothéquant leur avenir car les séquelles somatiques de la dénutrition peuvent se révéler même à long terme sous forme par exemple d’une ostéoporose ou de conséquences gravissîmes au niveau des dents
- **l’insertion sociale**, aussi bien l’insertion sociale extra-familiale, (avec souvent un arrêt de la scolarité lié à la durée des hospitalisations qui vont entraîner des redoublements et une perte de repères scolaires chez des sujets qui classiquement sont brillants sur le plan scolaire, une perte des liens avec les amis), que l’insertion dans la famille.

Les conséquences de cette pathologie sont aussi familiales. **L’anorexie mentale est une maladie chronique** qui va mobiliser les parents et les mettre en difficulté face à leur enfant. Les **conséquences sont lourdes sur le plan familial**, ainsi on constate **une dégradation des relations familiales et de la communication familiale** liée à cette pathologie, et pouvant aboutir à la mise au banc de la famille de la patients.

Enfin, **le coût de cette pathologie pour la société**, est **extrêmement important,** il est lié à la durée de la pathologie, qui nécessite une **prise en charge longue avec des soins généralement intensifs**.

Pour toutes ces raisons, **il apparaît donc** **extrêmement important de trouver des thérapeutiques ayant une efficacité plus importante sur l’anorexie mentale**, permettant à la fois de **soulager plus rapidement ces patients** et de les guérir plus rapidement, **afin de diminuer les conséquences de cette pathologie**, aussi bien **sur le plan psychologique** que **sur le plan somatique**, **sur le plan social** et **sur le plan familial**. Enfin, l’amélioration des thérapeutiques peut permettre **une diminution du nombre d’hospitalisation**s de ces patients et une diminution de la durée de soins intensifs permettant d’en **réduire le coût en termes humain et économique**.

# Données de la littérature

## Données de la littérature :

### Thérapie familiale et anorexie mentale: revue de la littérature

**L’intérêt pour la thérapie familiale dans le traitement de l’anorexie est ancien et important**. De nombreux auteurs ayant contribué au développement de la thérapie familiale tels Palazzoli, Minuchin, Stern l’ont appliqué avec enthousiasme à l’anorexie mentale. Ainsi en 1978 Minuchin , dans le cadre d’un essai ouvert sans groupe témoins, rapporte une efficacité de 86% de la thérapie familiale dans l’anorexie mentale. Certains ont même fait de la thérapie familiale des anorexiques un exemple paradigmatique (Dare et al, 1995). **Cependant si les articles sur les types de relations familiales dans l'anorexie et les discussions cliniques sur l’efficacité de la thérapie familiale dans cette pathologie sont très nombreux, cela contraste avec le peu d'études contrôlées d’évaluation des thérapies familiales dans l'anorexie mentale**. De plus les quelques études que nous avons retrouvées comparent les effets de la thérapie familiale à ceux de la thérapie individuelle, ou la considère dans le cadre de programmes thérapeutiques. Aucune étude n'évalue la thérapie familiale comme nous l’envisageons, c’est-à-dire comme une contribution thérapeutique supplémentaire et essentielle.

Nous avons retrouvé **trois équipes** impliquées dans ces recherches d’évaluation des thérapies familiales **entre 1966 et 1997, deux sont anglaises et une américaine**. Ce sont par ordre chronologique des publications :

- une équipe de l’Institute of Psychiatry et du Maudsley’s hospital ( Russell et al,1987,1993, Le grange et al 1992, Dare et al 1995) ayant réalisé quatre études sur la thérapie familiale systémique. (Voir tableau résumé 1 ci-dessous)
- une équipe du St George’s Hospital Medical School (Crisp et al, 1991, et Gowers et al 1994), ayant réalisé une étude sur la thérapie systémique. (Voir tableau résumé 2 ci-dessous)
- et enfin une équipe du Children hospital of Michigan (Robin et al, 1994,1995) ayant réalisé une étude sur la thérapie familiale comportementale. La technique de thérapie ici considérée n’est pas la même que celle que nous comptons employer, cependant nous citons ces études afin de réfléchir à leur méthodologie. (Voir tableau résumé 3 ci-dessous).

Tableau résumé 1

| **ANNEE** | **AUTEURS** | **CRITERES DIAGNOSTIC** | **GROUPES DE SUJETS** | **EFFECTIFS INCLUS** | | | **EFFECTIFS FINAUX** | **AGE** | **INSTRUMENTS** | **DUREE RECRUTEMENT** |
| --- | --- | --- | --- | --- | --- | --- | --- | --- | --- | --- |
| 1987 | RUSSELL et al | DSMI-III-R | - Début ≤ 18 ans et moins   de 3 ans   - Début ≤ 18 ans et plus de 3 ans d’évolution - Début ≥ 19 ans | Thérapie Individuelle 11  Thérapie Familiale 10  Thérapie Individuelle 7  Thérapie Familiale 8  Thérapie Individuelle 10  Thérapie Familiale 11 | | | 10  11  7  7  9  10 | ?  ?  ?  ?  ?  ? | - Poids / Poids théorique x 100 - Echelle de Morgan et Russell - Expressed Emotion | ? |
| 1992 | RUSSELL et al | DSMI-III-R | - 18 ans en post hospitalisation (NB : durée des troubles en moyenne 7 ans)   Durée d’évaluation ≥ 3 ans | | - Thérapie familiale - Psychothérapie psychanalytique - Thérapie de soutien   **Etude en cours lors de la publication** | | | | |  |
| 1992 | LE grange et al | DSM-III-R | Patients ≤ 19 ans durée évaluation < 18 mois   - Thérapie Familiale - Conseil Familial | 9  9  (2 hommes) | | | 9  9 | 15, 3 ±  1,8 | - Echelle de Morgan et Russell - FACES - Express Emotion |  |
| 1995 | DARE | DSM-III-R | Patients ≤ 18 ans  Durée d’évolution < 18 mois   - Thérapie Familiale - Conseil Familial | 20  20 | | étude en cours d’analyse | | | |  |
| 1995 | DARE | DSM-III-R | - Thérapie Familiale - Thérapie Individuelle - Thérapie de soutien | | étude en cours | | | | |  |

Tableau résumé 2

| **ANNEE** | **AUTEURS** | | **CRITERES DIAGNOSTIC** | | **GROUPES DE SUJETS** | **EFFECTIFS INCLUS** | **EFFECTIFS FINAUX** | **AGE** | **INSTRUMENTS** | **DUREE RECRUTEMENT** |
| --- | --- | --- | --- | --- | --- | --- | --- | --- | --- | --- |
| 1991 | | CRISP  et al | | DSM-III-R | 1. Hospitalisés 2. Thérapie individuelle + thérapie Familiale + conseil diététique 3. Thérapie de groupe pour le patient + thérapie de groupes pour les parents + conseil diététiques 4. Généraliste | 30  20  20  20 | 18  18  17  20 | 29,2 ± 4,9  21,2± 5,1  19,7 ± 2,6  21,9 ± 4,5 | - Echelle de Morgan et Russell | 4 ans |
| 1994 | | GOWERS et al | | DSM-III-R | 1 - Thérapie individuelle + thérapie Familiale + conseil diététique  2 - Thérapie de groupe pour le patient + thérapie de groupes pour les parents + conseil diététiques | 20  20 | 18  19 | - | - Echelle de Morgan et Russell | 4 ans |

Tableau résumé 3

| **ANNEE** | **AUTEURS** | | **CRITERES DIAGNOSTIC** | **GROUPES DE SUJETS** | **EFFECTIFS INCLUS** | **EFFECTIFS FINAUX** | | **AGE** | | **INSTRUMENTS** | | **DUREE RECRUTEMENT** | |
| --- | --- | --- | --- | --- | --- | --- | --- | --- | --- | --- | --- | --- | --- |
| 1994  étude  initiale | | ROBIN et al | DSM-III-R | 1 - Thérapie individuelle + consultation des parents  2 - Thérapie familiale | 12  12 | | 11  11 | | 14, 7 ± 2,7  13,9 ± 2,1 | | - BMI - PARQ - EDI - EAT - IBC | | ? |
| 1995  suivi  à 2 ans | | ROBIN et al | DSMI-III-R | 1 - Thérapie familiale  2 - Thérapie individuelle | 12  12 | | 11  9 | | 14,7 ± 4,7  13,9 ± 2,1 | | - BMI - PARQ - IBC | | ? |

- **Les études de l’Institute of Psychiatry et du Maudsley’s hospital :**

**La première étude ( Russell et al, 1987) :**

porte sur 57 anorexiques et 23 boulimiques. Ces sujets ont été recrutés suite à une hospitalisation pour trouble du comportement alimentaire, et la thérapie a été instituée en prévention des rechutes après hospitalisation et pour une durée de un an. Les thérapies étaient proposées à raison de un heure à chaque séance, en moyenne toutes les trois semaines, mais avec une adaptation à la situation clinique (plus rapprochée au début puis en fonction des besoins) Dans cette étude, les anorexiques étaient répartis en sous-groupes, en fonction de deux facteurs pronostiques reconnus comme déterminant de la guérison : l’âge et la durée de l’évolution de la maladie. Dans le sous-groupe I, la maladie a débuté avant l’âge de 18 ans inclus et dure depuis moins de 3 ans (N = 21) ; dans le sous-groupe II, la maladie a débuté avant l’âge de 18 ans inclus et dure depuis plus de 3 ans (N=15) ; dans le sous-groupe III, la maladie a débuté à l’âge de 19 ans ou plus (N=21) ; le sous-groupe IV est constitué de patientes boulimiques (N=23) dont nous ne parlerons pas ici. Cette étude opposait la thérapie familiale en référence à Minuchin et pratiquée par Christopher Dare, et à une thérapie individuelle de soutien non psychanalytique.

Les **auteurs montrent une meilleure efficacité de la thérapie familiale chez les sujets ayant débuté leur anorexie avant 18 ans et depuis moins de trois ans**. Ainsi on observe dans ce groupe lors du suivi à un an un " devenir bon ou intermédiaire" dans 9 cas sur 10 lors du traitement par thérapie familiale contre 2 cas sur 11 en cas de thérapie individuelle seule (p < 0,02). De même on observe que la thérapie familiale conduit à une amélioration significativement supérieure à celle de la thérapie individuelle pour le statut nutritionnel, le fonctionnement menstruel, l’ajustement psycho-sexuel, le statut socio-économique et le poids (25, 5 % contre 15 %, p < 0, 01).

Ceci n'est pas observé parmi les deux autres groupes de sujets (anorexie évoluant depuis plus de trois ans et/ ou ayant débuté après 19 ans). Cependant on doit remarquer que pour le groupe dont l'anorexie a débuté après 19 ans, le gain de poids est significativement supérieur (19, 5 % vers 5, 5 % p< 0,1) en cas de thérapie individuelle, mais ce gain de poids isolé n'est pas associé à une amélioration du devenir général du sujet.

Le suivi à cinq ans confirme ces conclusions qui sont stables ( Russell et al, 1993).

**Cette étude est considérée comme la plus remarquable qui soit parue à ce jour sur ce sujet**.(Yager et al, 1988)

**Leur deuxième étude** :

Elle concernait des adultes de plus de 18 ans présentant des troubles du comportement alimentaire (anorexie ou boulimie). Des patients présentaient des troubles anciens (la durée de l’évolution des troubles était en moyenne de 7 ans).

Ce sous-groupe correspond au dernier sous-groupe de leur première étude ( Russell et al, 1987).

Ces patients étaient inclus après une hospitalisation pour prise en charge de leurs troubles du comportement alimentaire. Ils étaient ensuite traités durant un an en psychothérapie.

Le traitement était attribué par randomisation stratifiée. Trois types de traitement étaient pris en compte dans cette étude : thérapie familiale, psychothérapie de type psychanalytique et thérapie de soutien individuelle.

Cette étude a montré que la thérapie familiale est significativement plus efficace en terme d’amélioration du devenir des patients que la thérapie individuelle ou la thérapie de soutien. La thérapie familiale semble plus utile pour les patients anorexiques dont l’anorexie a débuté durant l’adolescence (avant 19 ans).

Dans le sous-groupe de sujets dont les troubles du comportement alimentaire ont débuté à un âge de plus de 19 ans inclus, on ne retrouve pas de différence entre les trois groupes de traitement.

Les auteurs ont mis en place un suivi à 5 ans de cette étude, dont les résultats ne sont pas encore publiés.

**La troisième étude (Legrange et al 1992, Russell et al, 1992)** :

C’est une étude pilote ayant débouché sur une étude plus large. Ce travail concerne le sous-groupe pour lequel la thérapie familiale avait été montrée la plus efficace dans les études précédentes : les patients étaient plus jeunes, et inclus en ambulatoire sans avoir été hospitalisés. L’hypothèse était cette fois que la prise en charge ambulatoire en thérapie familiale pouvait tout à fait amener à une guérison des patients, sans qu’il y ait besoin d’hospitalisation.

Les auteurs comparent deux traitements : la thérapie familiale et le conseil familial (qui consiste en des consultations parentales sur le thème du trouble du comportement alimentaire de l’enfant). Pour ce dernier traitement, la patiente est reçue parallèlement en consultation individuelle.

Dans cette étude pilote, 18 patients anorexiques ont été inclus et attribués par randomisation, soit à la thérapie familiale, soit au conseil familial. Les résultats ne montrent pas de différence entre les deux types de traitement quelque soient les variables considérées, ce qui peut être dû au faible effectif. Les patients ayant mal répondu au traitement avaient des scores élevés aux facteurs d’ "expressed emotion" et insatisfaction de la FACES.

Suite à cette étude pilote, les auteurs ont continué leur essai. Ils ont inclus 40 patients de 18 ans ou moins. La durée moyenne des troubles était de 18 mois.

Le traitement durait un an et était exclusivement ambulatoire.

Les résultats complets de l’étude ayant suivi la phase préliminaire ne sont pas encore publiés. L’étude est partiellement résumée dans un article de Christopher Dare et al (1995).

**Quatrième étude du Maudsley (Dare et al 1995) :**

Les patients sont tous vus en ambulatoire, sans que cela fasse suite à une prise en charge en hospitalier. Ils sont randomisés à un type de traitement parmi quatre : la thérapie familiale, la psychothérapie focale d’orientation analytique, la thérapie cognitive analytique, ou la thérapie de soutien.

Cette étude est actuellement en cours au Maudsley et les résultats ne sont pas encore obtenus.

L’équipe du Maudsley a à ce jour la plus grande expérience de conduite des études sur la thérapie familiale dans l’anorexie mentale. **Cette équipe a accepté** **de nous conseiller dans l’élaboration de ce projet et dans sa réalisation.**

- **L’étude du St George’s Hospital Medical School (Crisp et al, 1991, Gowers et al 1994) :**

Nous citons ces études bien qu’elles n’évaluent pas l’efficacité de la thérapie familiale mais comparent des modalités de prise en charge ambulatoire versus prise en charge en hospitalier ou abstention thérapeutique. Les traitements ambulatoires avec thérapie familiale se sont révélés efficaces.

Cette étude concerne 90 patientes anorexiques mentales qui ont été réparties en 4 groupes de traitement par randomisation. Le premier groupe suit un traitement en hospitalier. Le deuxième groupe suit un traitement en ambulatoire, combinant une thérapie individuelle et une thérapie familiale, plus un conseil diététique. Le troisième groupe suit un traitement ambulatoire associant une psychothérapie de groupe pour le patient et une psychothérapie de groupe pour les parents, plus des conseils diététiques. Le quatrième groupe ne se voit pas attribuer de traitement et est réadressé à son médecin généraliste.

Les critères d’inclusion de ces patients étaient de répondre aux critères d’anorexie mentale du DSM-III-R, d'avoir une durée d’évolution de leur maladie de moins de 10 ans, d’avoir rédigé leur consentement par écrit pour entrer dans l’étude, et d’habiter à moins de 40 miles du centre de traitement.

Les auteurs ont utilisé l’échelle de Morgan et Russell. Cette échelle donne un score sur le statut nutritionnel, le fonctionnement menstruel, l’état mental et sexuel et l’adaptation socio-économique, en plus d'un score global.

Immédiatement après la randomisation, les patients et leurs parents étaient informés du type de traitement qui leur était attribué, ayant 10 jours pour y réfléchir et communiquer leur décision. Ceux qui ont accepté ce traitement étaient inscrits sur une liste d’attente et le traitement était débuté aussitôt que possible.

Ceux qui ont accepté ont été appelés "compliants" et ceux qui ont refusé ont été appelés "non compliants" ; aucun autre traitement que celui de l'étude ne leur a été proposé. Ces derniers étaient inclus dans les analyses de suivi en tant que non compliants, et restaient attribués à leur groupe de traitement respectif. Le suivi était prévu à 1 an, 2 ans et 5 ans après l’évaluation initiale. Cet article relate les résultats après 1 an de suivi, soit 5 ans d’étude. Les patientes étaient âgées en moyenne de 22 ans. La durée de la maladie était en moyenne de 39 mois. Il n’y avait pas de différence significative entre les groupes pour les évaluations initiales à l’échelle de Morgan et Russell. La plupart des patients n’ayant pas eu de traitement attribué, ont reçu un traitement ailleurs (groupe IV). Il y a eu des sorties d’essai dans les trois groupes, plus particulièrement dans le groupe de patientes suivies en ambulatoires. Les patientes hospitalisées l'ont été en moyenne 20 semaines. Elles ont ensuite été suivies en ambulatoire.

Tous les groupes de patients ont augmenté significativement leur poids. Les deux groupes de sujets ayant été pris en charge (groupe II et III) en ambulatoire, montrent une augmentation du poids significativement plus importante que dans le groupe IV (groupe sans traitement). Il est important de remarquer que, parmi les patientes compliants au traitement, celles hospitalisées ont pu atteindre un poids significativement plus élevé que dans les autres groupes. De plus elles ont pris moins de temps que dans les autres groupes (ambulatoire) pour atteindre ce poids maximum. Tous les groupes montrent une amélioration significative sur le test de Morgan et Russell.

Les auteurs notent une mauvaise observance du traitement qu'ils expliquent en particulier par l'attribution aléatoire de celui-ci.

Dans leur article de 1993, Gowers et al rapportent l’évolution à deux ans de l’un des groupes de patients pris en charge dans leur étude publiée en 1991 : le groupe pris en charge en thérapie individuelle ambulatoire et en thérapie familiale ambulatoire.

Sur les 20 sujets initialement inclus, seuls 10 ont pu suivre l’ensemble du traitement. Ceci diminue considérablement l'interprétabilité des résultats commentés dans cet article.

En terme de poids, il y a une amélioration significative à 1 an et 2 ans des sujets traités, mais aussi des sujets non traités. Cette amélioration est significativement plus importante dans le groupe de sujets traités. Il n’y a pas d’augmentation significative entre 1 an et 2 ans.

Les données concernant le devenir sur le plan physique, psychopathologique, social, sexuel et du fonctionnement en général sur l’échelle de Morgan et Russell, montrent une amélioration au fil du temps. Cette amélioration est significativement plus importante à 2 ans dans le groupe traité par rapport au groupe non traité, en ce qui concerne l’amélioration du fonctionnement social. Ils décrivent ensuite l'évolution des sujets en fonction des facteurs pronostiques connus, à savoir le poids, l’âge, la chronicité des troubles, la présence ou non de vomissements et la compliance au traitement. Aucun de ces facteurs n'influence ici significativement le devenir.

Les améliorations présentées par ces patients à un an se retrouvent à deux ans. L’amélioration est appréciée en terme d’adaptation sociale, de fonctionnement psycho-sexuel, et sur les mesures physiques. **Les auteurs insistent sur l'importance de dégager les facteurs prédictifs de succès des traitements dans les études à venir.**

- **L’étude du Children hospital of Michigan (Robin et al, 1994,1995) :**

Une autre équipe pratiquant des thérapies d’orientation théorique différente des précédentes et de la notre, a réalisé une étude comparant l'efficacité de la thérapie familiale comportementale versus la thérapie individuelle comportementale (durant de 12 à 18 mois). Bien que l’orientation théorique qui sous-tends ces traitements soit différente nous en présentons ici les résultats dans un soucis d’objectivité. L’hypothèse initiale des auteurs était que la thérapie familiale doit permettre une meilleure évolution que la thérapie individuelle sur le poids, les attitudes alimentaires et les mesures familiales, alors que la thérapie individuelle doit donner une amélioration supérieure sur le fonctionnement individuel, la dépression et les attitudes alimentaires intériorisées.

Cette étude concerne 22 jeunes filles anorexiques selon les critères DSM-III-R âgées de 12 ans à 19 ans et malades depuis moins de un an. Ces caractéristiques cliniques correspondent à un sous-groupe de la population dont Russell a démontré la meilleure indication par la thérapie familiale (maladie ayant débuté avant ou à l’âge de 18 ans et durant depuis moins de 3 ans). Les différentes évaluations réalisées comprennent :

- une mesure des conflits familiaux avec un questionnaire appelé le ARQ : Parents-Adolescent-Relation Ship-Questionnaire. Cet outil évalue deux types de conflits, les conflits généraux et les conflits autour des troubles du comportement alimentaire.
- une hétéro-évaluation clinique BMI, attitudes alimentaires avec l’EAT (Eating Attitude Test),
- l'insatisfaction concernant la silhouette avec le BSQ (Body Shape Questionnaire),
- l’échelle de insatisfaction de l’EDI (Eating Disorder Inventory), et
- l’Ineffectiveness, Interpersonal Distrust, and Interoceptive Ewareness Scales pour mesurer le fonctionnement psychologique individuel,
- le BDI (Beck Depression Inventory), et le Child Behavior Checklist Internalizing Behavior Problems Score pour mesurer la psychopathologie.

Les patients ont été suivi un an après la fin du traitement et réévalués. Chaque groupe était composé de 11 patientes d'âge moyen de 14,7 ans. Seuls 9 sujets du groupe de thérapie individuelle ont terminé l’étude alors que tous les sujets du groupe de thérapie familiale ont pu être évalués après 1 an de suivi. Sur 24 sujets inclus, 22 ont accepté de participer à l'étude et 20 l’ont terminé.

Sept adolescents ont reçu en moyenne 15,9 mois de thérapie et ont été évalué à 12 mois pour le suivi. Au cours de la thérapie individuelle, les parents rencontraient de façon bi-mensuelle le thérapeute. On constate une amélioration du BMI des sujets des deux groupes entre le début de la prise en charge et la fin du suivi. Cette amélioration est significativement supérieure en cas de thérapie familiale (augmentation du BMI de 5 contre 2,6, p<0,01). Cependant si l'on considère le critère clinique de guérison qui consiste à atteindre la normalisation du poids et / ou des règles, il n'y a pas de différence significative entre les deux groupes. De même on observe une diminution des conflits à propos de l'alimentation, une amélioration des attitudes alimentaires et de l'insatisfaction concernant la silhouette après le traitement (persistant lors du suivi), mais ne différant pas entre le groupe de thérapie familiale et le groupe de thérapie individuelle. **La supériorité de la thérapie familiale sur la thérapie individuelle dans le traitement de l'anorexie mentale n'est pas mise en évidence dans cette étude contrairement au travail de Russell, alors que les sujets pris en compte correspondent presque aux même caractéristiques. A cela on peut voir trois raisons : le faible nombre de sujets considérés (mais identique à Russell et al 1987), les techniques différentes de thérapie (thérapie systémique pour Russell et comportementale ici), la présence d’une consultation parentale bimensuelle qui peut diminuer la différence observable entre les deux groupes.**

Les auteurs n’observent donc pas de confirmation de leurs hypothèses initiales. Ils concluent que thérapie individuelle et thérapie familiale influencent toutes deux positivement l’évolution de l’anorexie mentale quand au BMI, au retour des règles et aux interactions familiales entre les adolescents anorexiques et leurs parents. Cette étude soutient aussi le fait que ces améliorations significatives observées après traitement sont maintenues à 1 an après l’arrêt de ce traitement.

Cependant, ils soulignent que la thérapie familiale améliore davantage la communication positive entre mère et fille, et diminue la communication négative, significativement plus que la thérapie individuelle.

Sur le plan méthodologique, cette étude nous semble critiquable du fait du petit nombre de sujets pris en compte, et des sujets disparus en cours d'étude. Il semble difficile de donner des conclusions compte tenu de ces éléments. Enfin, on se doit de remarquer que les critères de guérison définis par les auteurs ne sont pas classiques. Ils reposent soit sur le fait d’atteindre un poids fixé par un pédiatre, soit sur le fait que la patiente a de nouveau ses règles, qui sont deux critères a priori différents.

**EN CONCLUSION**

Nous répéterons ce que nous disions en début de cette exposé : si de **nombreuses voix s’accordent pour souligner l’importance d’une approche familiale dans les troubles du comportement alimentaire,** la plupart se basent sur leur expérience clinique subjective pour l'affirmer. **Les études menées sur le sujet sont peu nombreuses et pas toujours très rigoureuses**. Russell souligne en 1993, que les traitements prescrits dans l’anorexie mentale ont rarement fait l’objet d’étude clinique standardisée. **Les pratiques traditionnelles ont rarement été évaluées**. De même Crip (1991) souligne que les **études d’efficacité sur le traitement de l’anorexie mentale sont rares** et n’ont jamais été conduites à long terme. Celles qui existent concernent le plus souvent des essais pharmacologiques ou des essais sur les thérapies comportementales.

La **nécessité de réaliser de nouvelles études avec des méthodologies plus solides portant sur la thérapie familiale**, plus particulièrement chez les patients anorexiques, est signalée par de nombreux auteurs (Diamond et al, 1996). En effet ils soulignent qu’il est important de réaliser ces études afin d’une part, de répliquer les résultats des quelques études antérieures (tels que ceux de l’étude de Russell et al, 1987), et d’autre part, d’utiliser les connaissances en psychopathologie et les procédures de recherche pour développer conjointement des modèles de traitement à partir de théories testées permettant des résultats plus robustes.

### Fonctionnement familial

L’importance du fonctionnement familial sur la santé psychologique et physique des enfants et des adolescents atteints de maladie chronique a été depuis longtemps mis en évidence (Pless et al 1972), plus particulièrement dans le cadre de l’anorexie mentale. Nous relaterons ici les principales caractéristiques observées dans le fonctionnement familial en cas d’anorexie mentale.

#### Articles relatant des observations cliniques

**L'impossibilité à accepter la séparation et l'autonomie** est l'un des traits de fonctionnement des familles d'anorexiques décrites par les systématiciens (citons les principaux: Kalucy et al, 1977, Minuchin 1978, Selvini-Palazzoli 1978,). Cette impossibilité est la même pour chacun dans la famille.

Pour chaque membre de la famille, le sentiment d'appartenance est supérieur au sentiment d'autonomie, ce qui implique que tout ce qui mettrait en danger l'homéostasie familiale n'est pas autorisé à être exprimé. Pour Minuchin (1978), dans une majorité de familles d'anorexiques, on retrouverait les caractéristiques de fonctionnement de ce qu'il a appelé une famille “ psychosomatique ”:

- **L'enchevêtrement**
- **La surprotection**
- **La rigidité**
- **L'évitement des conflits**

La patiente est en général impliquée dans un conflit parental caché. M. Selvini (1982) et H. Bruch (1978, 1982) arrivent aux mêmes conclusions : l'expression de l'individualité n'est pas encouragée, **l'autonomie et l'indépendance sont sacrifiées à la loyauté envers la famille.** Ce sentiment très important d'identité de la famille aux dépens de l'identité individuelle est partagé par la **génération des grands parents souvent très présente, voire envahissante.**

Selvini (1978) pointe aussi la tendance à la valorisation du sacrifice de soi, à la loyauté extrême, et au maintien à tout prix des apparences, quelque soit le désespoir sous-jacent.

Selon J.P. Martinez, les **individus disparaissent au profit des fonctions** qu'ils assurent "comme des partenaires sur une scène de théâtre". **Ce qui compte, c'est la perpétuation des règles du jeu qui permettent de résister au changement ; les lois (règles hiérarchiquement supérieures) seraient alors digérées au profit du maintien de ces règles : loi des différences de génération, dénégation de la réalité de la mort par exemple.**

**Dans ce type de famille, l'anorexie permet à la fois de protéger l'homéostasie et de conférer une identité à la patiente à l'intérieur de la famille** (ce qui renforce sa difficulté à abandonner ce symptôme).

On retrouvons classiquement dans les familles de ces patientes anorexiques certaines des caractéristiques suivantes :

- La famille est souvent décrite comme une **famille idéale** où régnait une parfaite entente qui n'est troublée que par l'apparition brutale de la maladie, vécue comme un effondrement inattendu, inexplicable.
- Les parents s'enorgueillissent d'avoir donné à leurs enfants “ tout pour être heureux ”, et sont effarés de l'irruption incompréhensible des difficultés majeures de leur fille.

Cette souffrance des parents va majorer leurs tendances défensives et la famille va être prise dans une série d'interactions en chaîne où le symptôme provoque **une augmentation des tensions** qui ne feront **que renforcer la conduite anorexique.**

- Ces familles sont souvent très fermées sur elles-mêmes, **dans un isolement relationnel important**, cette unité fragile est maintenue grâce à l'évitement des conflits, à la non-expression des sentiments “négatifs ” tels que l'agressivité ou la colère. Les interrelations sont lisses.
- Le corps, comme dans les familles dites “ **psychosomatiques** ”, est l'objet d'une grande préoccupation. L'image du corps, le poids, la nourriture sont des contenus de communication importants. L'alimentation a une place fondamentale dans les échanges familiaux. La **nourriture est un moyen de définir les relations, d'exprimer l'amour ou la colère. C'est aussi un moyen de maintenir un contrôle des parents sur la patiente.**

Les repas sont parfois la seule occasion de réunion de la famille. Ils sont une préoccupation centrale pour chaque membre de la famille, le lieu où s'exercent les rituels familiaux. **La nourriture et les relations qui en résultent perpétuent aussi l'attachement exclusif de l'enfant à sa mère.**

- Les **affects intenses (colère, jalousie, anxiété, dépression) sont difficilement supportés,** et le stress provoque souvent des décharges explosives ou des maladies psychosomatiques (H.T.A., problèmes cardiovasculaires...).

L'ignorance et la banalisation des difficultés sont de règle, et seule une inflation de la symptomatologie, qui va parfois jusqu'à mettre en jeu le pronostic vital, peut bouleverser le pseudo-équilibre familial. Les parents craignent d'entrer en conflit avec leurs enfants et ont **une alternance inadéquate d'attitudes permissives et coercitives**. Les **mères** sont décrites comme à la fois rigides et vulnérables ; les **pères** ayant une intense difficulté à fantasmer, interviennent longtemps après le début du symptôme et s'en tiennent à des aspects très concrets.

Dans toutes ces familles, on retrouve une **difficulté à aborder la séparation,** ce qui contribue à l'apparition des symptômes à l'adolescence, période clé des processus de séparation-individuation

Les troubles apparaissent lorsque l'adolescente est encore très investie par sa famille d'origine, même si elle vit à l'extérieur, dans une pseudo‑indépendance. Malgré les apparences d'autonomie ou de rébellion, ces patientes sont en général extrêmement dévouées et loyales au système familial ; elles sont en quelque sorte les gardiennes des traditions et des mythes familiaux.

#### Etudes contrôlées concernant les relations familiales

Les études sur les relations familiales et les interactions au sein des familles des patients présentant une anorexie mentale, sont nombreuses. par contre, il est difficile d’en tirer des conclusions simples, car la plupart du temps les dimensions explorées et les instruments utilisés sont différents. Ces études sont de deux types, celles qui concernent les perceptions des patients concernant les relations familiales et celles concernant les perceptions des parents.

##### Perceptions des patients concernant les relations familiales

**Palmer et al. (1988)** utilisant le Parent Bonding Instrument (PBI), montre que les patients anorexiques ont le souvenir d’une mère moins attentive dans leur enfance que les témoins.

**Garner et al. (1985)** ont fait une étude en utilisant le Family Assessment Mesure (FAM).

Ils trouvent que les patients anorexiques boulimiques et les patients boulimiques, décrivent leur famille comme ayant plus de difficultés sur toutes les dimensions mesurées par cet instrument : expression des affects, investissement affectif, contrôle, communication, accomplissement du devoir, performances, valeurs et normes, contrairement aux anorexiques restrictives qui n’ont pas ces caractéristiques.

**Waller et al. (1989)** montrent, en utilisant le Family Assessment Device (FAD), que les patients anorexiques rapportent plus de relations familiales de mauvaise qualité que les contrôles.

**Humphrey et al. (1986),** montrent que les anorexiques boulimiques, contrairement aux anorexiques restrictives et aux témoins, trouvent leurs parents plus critiques, rejetant et négligeant à leur égard, et disent aussi ressentir un manque d’affection et d’empathie de la part de leurs parents.

Les mesures sont réalisées avec le Beased On The Structural Analysis of Social Beahavior (SASB).

**Waller et al. (1990)**, utilisant la Family Adaptability and Cohesion Evaluation Scale (FACES), retrouvent que ces patients anorexiques perçoivent leur famille comme moins adaptable et ayant une cohésion moins importante que les sujets contrôles.

**Russell et al. (1992).** a mesuré les liens des patients adolescents anorexiques et de leurs parents avec le Parental Bounding Instrument (PBI) . Ils ont montré que les scores des patients anorexiques sont plus semblables à ceux des contrôles qu’à ceux des patients. Les patients anorexiques décrivent leurs parents tels que leur père et leur mère prennent plus soin d’eux et que leurs mères sont moins surprotectrices que dans le groupe des patients psychiatriques. Ce qui remet en question l’hypothèse classique dans l’anorexie selon laquelle les parents d’anorexiques sont hyperprotecteurs mais cela reste à répliquer.

Dans un article de synthèse sur les relations familiales dans l’anorexie mentale, Humphrey (1992),conclut sur l’ensemble de ces études que la perception de la famille par les patients anorexiques est globalement plus perturbée que celle des familles au sein desquelles il n’y a pas de patients anorexiques.

##### Perceptions des relations familiales par les parents et les patients.

**Strober, en 1980**, trouve que les parents des patients anorexiques boulimiques sont plus conflictuels, désorganisés et insatisfaits de leur mariage. Il trouve aussi que les parents des patients anorexiques boulimiques sont moins unis que ceux des parents anorexiques restrictives. Pour cela il utilise deux instruments, le FES : Family Environnement Scale et le SMAT : Short Marital Adjustement Test.

**Garfinkel, en 1983**, compare des parents de sujets anorexiques et des témoins. Il utilise pour cela le FAM, Family Assessement Mesure. Il montre que les patients anorexiques et leur mère voient tous deux la famille comme ayant plus de problèmes dans l’accomplissement des devoirs, les performances, la communication, l’expression des affects que les témoins.

**Stern, en 1989**, utilisant le FES, montre que les évaluations des parents sont généralement plus positives que celles de leurs enfants. On perçoit cependant, dans l’évaluation des parents, les mêmes déficits dans les relations familiales que leurs filles. Les parents des patientes anorexiques et les patientes anorexiques sont moins expansifs et moins orientés vers les activités récréatives que les contrôles.

**Kog et al. (1985)**, utilisant la FACES montre que les patients anorexiques rapportent des conflits plus importants et plus de désorganisation familiale que ne le font les contrôles.

**Humphrey**, utilisant la FACES et la FES, trouve que les pères et mères et les patientes anorexiques restrictives et boulimiques, rapportent que leur famille font une moins bonne cohésion et sont plus chaotiques que les familles des contrôles.

**En conclusion**

**Sur ces études, il semble assez clair que les relations familiales mesurées auprès des patients anorexiques et de leurs parents semblent perturbées**. Ces perturbations sont perçues de façon plus aiguë par les patientes, mais elles sont à une moindre mesure relatées par les parents aussi. Les mesurer est extrêmement important (essentiel ou capital), car elles semblent être un facteur pronostic important dans le devenir des patients sur lequel intervient la thérapie familiale que nous nous proposons d’étudier ici.

Il apparaît que la FACES est l’instrument le plus souvent utilisé. De plus il est important d’utiliser à la fois un auto et un hétéro-questionnaire pour mesurer les relations familiales dans la mesure où certains auteurs mettent en évidence un déni des troubles dans les auto-questionnaires.

### Symptomatologie et facteurs de pronostic

Le devenir des patients, qui est notre critère d’évaluation principal, est influencé par des **facteurs de pronostiques** connus, que nous devons par conséquent prendre en compte dans cette étude.

Ainsi le sous-groupe des **anorexiques boulimiques** se caractériserait par un devenir plus grave (Casper et al. 1980 ; Strober et al. 1982).

De plus, ont été individualisés des facteurs de pronostic défavorables tels qu'une **longue durée d’évolution des troubles, un poids bas au début du traitement, des éléments dépressifs et des comportements compulsifs** fréquemment rapportés dans la littérature (Bemis 1978; Crisp et al., 1979; Deter et al., 1989; Halmi et al. 1973; Herzog et al. 1988; Hsu et al. 1979; Hall et al. 1984; Morgan et al. 1983; Seidensrtricker & Tzagournis 1968).

Les symptômes significatifs signalés dans la littérature comme **critères d'évolution** favorables dans l'anorexie sont la **normalisation du poids**, des **cycles menstruels** et **du comportement alimentaire**. Dans une étude récente portant sur le devenir de 129 cas (Ph. Jeammet et al. 1991) avec un recul de 4 à 20 ans et une moyenne de 11 ans, nous trouvons une répartition des modalités évolutives comparables à la fourchette optimale des études étrangères similaires, soit à 80 une normalisation du poids et des conduites alimentaires, à 70 % un retour des règles, mais un peu moins de 50 % d'états psychologiques jugés satisfaisants. Les troubles psychiatriques susceptibles d'apparaître dans les suites de l'anorexie sont très variables et concernent pratiquement toute la nosographie. Néanmoins, **les plus habituels sont de l'ordre de la dépression**, des **phobies invalidantes** restreignant beaucoup les contacts affectifs et sociaux, et de la **pathologie obsessionnelle compulsive** (Godart, 1994).

### Caractéristiques familiales et facteurs de pronostic.

Aucun facteur n’a pu être clairement individualisé. Crisp et al (1974) ont retrouvé que les mères des patients ayant un **mauvais devenir avaient des scores de dépression plus élevés** sur les échelles de personnalité. Nous souhaitons évaluer ce facteur pronostique, en fonction de la comorbidité familiale de dépression ( Russell et al 1987).

De multiples hypothèses ont été faites à ce jour sur le rôle de la famille dans le développement ou la pérennisation des troubles du comportement alimentaire. Parmi ces hypothèses, certaines concernent la transmission familiale de ces troubles et des pathologies qui leurs sont fréquemment associés à savoir les troubles anxieux , les troubles dépressifs, et les abus de substances (alcoolisme et toxicomanie). Tout et son contraire ont été dit sur la transmission familiale de l'anorexie et de ces troubles. Nous n'avons pas retrouvé d'études portant à la fois sur la transmission familiale des pathologies thymiques, anxieuses et de l'alcoolisme chez les sujets anorexiques. Ces sujets sont généralement traités séparément. Ce point nous semble regrettable car on sait que alcoolisme et troubles anxieux sembles liés quant à leur transmission familiale (Merikangas et al 1985), et que la comorbidité entre dépression et troubles anxieux est fréquente.

**Au total les études sur ces sujets sont peu nombreuses, et quand elles existent les résultats sont contradictoires.**

Enfin la **transmission familiale des troubles obsessionnels, des troubles thymiques, de l'alcoolisme ainsi que celle de l'anorexie**, semble assez uniformément reconnue, sans que l'on puisse encore déterminer l'importance de chaque facteur, environnemental ou proprement psychique, ( Wiroken et al 1980 ; Hudson et al 1983 ; Rivines et al 1984 ; Merikangas et al 1985 ; Levy et al, 1989 ; Holden et al 1990 ; Strober et al, 1991 ; Pasquale et al, 1994 ; Kendler 1995 ; Walters et Kendler 1995). Dans la mesure où la comorbidité individuelle et familiale est un facteur pronostique important nous nous proposons d'investiguer ces dimensions.

### Adaptation sociale et facteurs de pronostic.

Les indices prédictifs d'involution prolongée des T.C.A. sont : **une mauvaise adaptation sociale au début de la maladie** (Hsu et al. 1979, Engel et al. 1987), **de mauvaises relations avec la famille d'origine** (Morgan et Russell 1975), ainsi qu'une **dépendance marquée vis-à-vis** d'elle. Engel (1990) considère que les patientes parvenant au moins à de nouvelles relations sociales, pendant ou après la thérapie, ont un bon pronostic.

### Alexithymie et maladies à composante psychosomatique

.Selon les hypothèses actuelles les troubles psychosomatiques sont liés à une carence de la mentalisation, marqué par un mode de fonctionnement mental nommé opératoire (pauvreté de la vie fantasmatique, diminution des capacités d’élaboration et d’association...),mesurable par une dimension appelée alexithymie, accompagné d’une dépression dite essentielle (sans symptômes, avec effacement du fonctionnement mental simulant une normalité extrême), (P. Marty, 1963).

C'est lors de la [Xe Conférence Européenne de Psychosomatique, à Vienne en 1972, que Sifneos propose le **terme d'alexithymie pour désigner le fonctionnement de nombreux patients souffrant d'affections organiques chroniques à forte composante psychosomatique**. Etymologiquement, alexithymie, dérivée du grec, **renvoie à l'absence de mots pour exprimer les émotions**: a = absence, lexis = mot, thymos = humeur, affectivité, sentiment, émotion. (Farges ,1996).

Sifneos (1973, 1977, 1993)., présente ainsi son concept: «une vie fantasmatique pauvre avec comme résultat une forme de pensée utilitaire, une tendance à utiliser l'action pour éviter les conflits et les situations stressantes, une restriction marquée dans l'expression des émotions et particulièrement une difficulté à trouver les mots pour décrire ses sentiments».

Selon les circonstances et la personnalité, le fonctionnement alexithymique correspondrait soit à un trait, soit à un état. (Farges, 1996). C'est pour cette raison que Freyberger (1977) propose de distinguer alexithymie primaire et alexithymie secondaire.

- L'alexithymie primaire désigne un facteur prédisposant à l'éclosion et l'évolution d'un trouble psychosomatique. Cette forme est considérée comme «innée», rebelle à la plupart des traitements et déterminante. Dans cette perspective, l'alexithymie est un «trait" de personnalité, que ce dernier soit déterminé génétiquement ou qu'il soit largement redevable aux particularités des relations affectives précoces qui ont marqué le développement de l'individu. L'alexithymie primaire signerait une insuffisance ou une fragilité du système des défenses psychiques de l'individu.
- L'alexithymie secondaire est la conséquence de facteurs traumatiques. Elle est définie par un état apparaissant chez des patients qui présentent une pathologie somatique grave (dialyse rénale, réanimation, cancer) ou qui sont soumis à des stress d'une autre nature que la maladie (situation de catastrophe). Cet état, qui est donc une réaction à l'angoisse suscitée par la maladie ou le traumatisme peut être transitoire (dans le cas de maladie) ou permanent (dans les suites de traumatisme). L'alexithymie secondaire protégerait contre la signification émotionnelle et la gravité de la maladie ou de l'événement, elle est assimilée à un mécanisme de défense.

L'alexithymie secondaire est un «état" style défensif se mettant en place dans certaines circonstances particulières, lorsque l'expérience reçue dépasse les capacités habituelles d'adaptation (états de stress post‑traumatique) ou lorsque la réalité quotidienne est potentiellement source d'une angoisse déstructurante (survenue d'une maladie particulièrement douloureuse car handicapante, ou mettant en jeu à brève échéance le pronostic vital).

La prévalence de **l'alexithymie serait significativement plus élevée au cours de l'anorexie mentale** par rapport à la population générale même en l'absence de dépression (Jimerson, 1994).

De nombreux auteurs (Marty et de M'Uzan, 1963) considèrent que l**'alexithymie** (mode de pensée particulier centré sur le concret, avec difficultés de représentation mentale des émotions) est un **facteur de risque pour les conduites addictives comme les troubles des comportements alimentaires**. La conduite addictive constituerait un mécanisme compensateur du fait de la faiblesse des mécanismes de défense et de l'incapacité du moi à réguler et moduler les émotions.

**L'alexithymie ne serait corrélée ni à la sévérité de la maladie, ni à l'amaigrissement, ni à la fréquence des vomissements**. Lorsque l'on utilise le seuil de 74 à la TAS, pour identifier les sujets alexithymiques, **le pourcentage d'alexithymiques chez les anorexiques oscille entre 63 % et 77 %** (Bourke, 1992; Cocrane, 1993; Laquatra, 1994).

Dans un échantillon de 114 femmes présentant des troubles du comportement alimentaire, Cochrane (1993) a différencié les **anorexiques strictes**, les boulimiques strictes, les **anorexiques‑boulimiques** et les TCA non spécifiques. Si les résultats montrent un score moyen à la TAS **significativement supérieur à celui d'une population standard d'étudiantes**, aucune distinction n'a pu être faite entre les différents sous‑groupes.

Cochrane (1993) et Laquatra (1994) ne sont pas davantage parvenus à établir de corrélation entre le score d'alexithymie et les différents facteurs de gravité des troubles alimentaires, tels que la durée de la maladie, le déficit pondéral, la fréquence des vomissements ou des accès boulimiques.

Laquatra (1994) a observé sur un échantillon de 308 étudiantes, **une corrélation entre le score d'alexithymie et la présence de troubles alimentaires identifiés au moyen de 1'EDI** (Inventaire des Troubles Alimentaires). Il ressort aussi de cette étude une corrélation positive entre la note globale d'alexithymie et la fréquence des idées suicidaires.

Il n’existe pas à notre connaissance d’étude portant sur la concurrence de l’alexithymie et des maladies psychosomatiques chez les patients anorexiques et leurs famille.

analyses concernant la cooccurrence de maladies psychosomatiques chez les patientes présentant des troubles des conduites alimentaires et leur famille nous semble donc particulièrement intéressante..

# Objectifs de la recherche (hypothèses testées, objectif primaire, objectifs secondaires)

## Hypothèses testées

### Première hypothèse

Notre hypothèse principale est que **la thérapie familiale adjointe aux soins classiquement proposés après une hospitalisation pour anorexie mentale permet d’en favoriser l'évolution.**

Cette efficacité peut être mise en évidence sur le plan clinique par une amélioration de :

- la symptomatologie alimentaire, en terme de rapidité d’amélioration et de devenir
- la psychopathologie générale
- la symptomatologie anxieuse et dépressive
- l’adaptation psychosociale des patients
- la qualité du fonctionnement familial

### Deuxième hypothèse

Les **facteurs familiaux habituels de mauvais pronostic** que sont **l'adaptation sociale au sein de la famille** et , **la mauvaise communication familiale** sont compensés par la mise en place d'une thérapie familiale.

## Objectif du projet

### Objectif primaire

Cette recherche se fixe pour objectif principal **d'étudier si l’adjonction d’une thérapie familiale aux soins classiquement proposés après une hospitalisation pour anorexie mentale permet d’en favoriser l'évolution.**

Pour cela nous projetons de réaliser une **étude clinique randomisée, prospective**, évaluant **l’efficacité de l’adjonction d’une psychothérapie familiale au programme de soins classiquement proposé dans l’anorexie mentale**.

Cet objectif sera poursuivi à travers plusieurs dimensions car le devenir des sujets s’apprécie sur plusieurs axes :

- Nature et gravité des symptômes alimentaires,
- Symptomatologie anxio-dépressive,
- Facteurs psycho-sociaux (adaptation sociale),
- Qualité des relations familiales.

On cherchera à déterminer :

- La manière dont la thérapie familiale influence ces différents axes

#### Effet du traitement sur la symptomatologie alimentaire

L’objectif est d’étudier **l’évolution des symptômes alimentaires** (poids, peur intense de prendre du poids, altération de la perception du poids ou de la forme de son propre corps, influence excessive du poids ou de l'apparence corporelle sur l'estime de soi, déni de la gravité de la maigreur actuelle, aménorrhée), **comparativement dans les deux groupes de sujets**. Il s'agit de réfléchir en terme de rapidité d’amélioration et de devenir, pour déterminer si la **thérapie familiale permet une amélioration plus rapide et plus importante de ces éléments**.

#### Effet du traitement sur la symptomatologie anxieuse et dépressive

On évaluera la **symptomatologie anxieuse et dépressive** avant l’instauration de la thérapie familiale et on essaiera de **mettre en évidence un effet de la thérapie familiale sur cette symptomatologie** intriquée à l’anorexie mentale.

#### Effet du traitement sur la symptomatologie psychosociale

Ce chapitre de l’étude se penchera sur **l’évolution de l’adaptation psychosociale** des patients en terme de devenir, pour déterminer si **la thérapie familiale permet une meilleure adaptation sociale des sujets.**

#### Effet du traitement sur le fonctionnement familial

Nous tenterons de mettre en évidence une **modification qualitative des relations familiales liée à la thérapie familiale**. Ainsi nous déterminerons si ces familles peuvent faire preuve d’une plus grande souplesse dans leurs interactions, en fonction d'une augmentation de l’autonomie de chacun, et d’une amélioration de la communication entre les membres de la famille.

Pour chacun des trois points précédemment cités ( nature et gravité des symptômes alimentaires, symptomatologie anxio-dépressive, adaptation sociale) **nous tenterons de corréler l’évolution constatée avec les modifications de communication familiale observées**.

Dans la mesure où il existe actuellement un consensus sur le fait **qu’il n’y a pas de famille d’anorexique typique,** (Vanderlinden et al, 1991) les objectifs de la thérapie pour chaque famille ne peuvent se concevoir qu’individuellement. Aussi nous proposons **d’évaluer, à l’entrée dans le traitement, des objectifs de travail dans les domaines précités, et d’évaluer tous les l’état de ces objectifs pour chaque famille.**

Il s'agit aussi de décrire les familles et les sujets pour lesquels l'issue de la thérapie s'est révélée corrélée avec une amélioration, **pour mieux en cerner les indications**.

### Objectifs secondaires

#### Evaluation comorbidité individuelle et familiale

**La comorbidité individuelle et familiale** (dépression, anxiété, dépendance alcoolique et toxicomaniaque, antécédents psychosomatiques et alexithymie) **pour la mettre en lien avec les profils d’évolution « favorable ou défavorable » et la thérapie familiale.**

#### Effet du traitement sur la symptomatologie psychosociale

Ce chapitre de l’étude se penchera sur **l’adaptation psychosociale** des patients et de leurs parents, pour déterminer si **elles sont liées.**

# plan expérimental :

## Choix du plan expérimental et justification

### Plan de l'étude

Cette recherche se fixe pour objectif de **comparer deux programmes de traitement proposés dans l’anorexie mentale familiale après une hospitalisation : le traitement classique seul et le traitement classique auquel on associe une thérapie familiale**. La **thérapie familiale sera instaurée après l’hospitalisation,** car il a été montré que son instauration avant une renutrition , à la phase aiguë de la maladie, n’est pas bénéfique et peut entraver le processus psychothérapique, ( Danzinger et al, 1989).

Nous nous proposons de réaliser **une étude prospective unicentrique randomisée, portant sur deux groupes de sujets anorexiques mentales issues de la population des anorexiques hospitalisées dans notre service.** **Le premier groupe suivra un traitement classique de l'anorexie mentale et un second groupe suivra ce même traitement plus une thérapie familiale** . Ces deux groupes de patients feront l'objet de comparaisons statistiques. Bien entendu cet essai ne peut se dérouler en double aveugle, dans la mesure où les thérapeutes et les patients savent s'ils suivent ou non une thérapie familiale.

### Randomisation

#### Moment

Le tirage au sort sera réalisé à la fin de l'hospitalisation, après acceptation de participation à l’étude, et avant la sortie pour éviter les perdus de vue. La thérapie familiale sera débutée à la sortie.

#### Modalités

Le tirage au sort ne sera ni ajusté ni stratifié. Le traitement ne sera pas délivré à l’aveugle.

## Critères de sélection des personnes, mode de recrutement

### Critères d'inclusion

- **Pathologie**

Seront considérées :

- tous les patients anorexiques mentales selon les critères du DSM-IV (Anorexia Nervosa Type restrictif ou avec crises de boulimie) 307.1(voir annexe 2)
- acceptant, ainsi que de leurs parents de participer à l'étude (consentements signés),
- dont l’anorexie mentale a débuté strictement avant 19 ans (18 ans ou moins),
- et dont la durée d’évolution est inférieure ou égale à 3 ans le jour de l’hospitalisation.
- **Sujets**

Seront exclusivement considérés les sujets :

- de sexe féminin,
- après une hospitalisation pour anorexie mentale dans le service du Pr Jeammet.
- habitant ainsi que leurs parents Paris ou sa banlieue
- **Traitements**
- Les patients ne recevront aucun autre traitement psychothérapique familial pour leur anorexie que ceux prescrits pour cet essai. Les thérapies individuelles seront prises en compte lors des analyses.
- En cas d'anxiété ou de dépression, les troubles pourront bénéficier d'une prescription complémentaire de psychotrope qui devra être mentionnée dans le dossier.
- Les traitements antérieurement suivis par les sujets et les traitements pour d'autres affections devront être décrits.
- Les parents pourront s'ils le souhaitent continuer à participer au groupe de parents qui leur est classiquement ouvert, même après la sortie d'hospitalisation de leur enfant .

### Critères de non inclusion

- **Pathologie**

Sont exclus les sujets pour lesquels est porté un des diagnostics suivants :

- état psychotique avéré,
- arriération mentale,
- trouble organique cérébral,
- pathologie métabolique interférant avec l'alimentation ou sa régulation (la plus fréquemment rencontrée étant le diabète).
- **Sujets**

Les sujets ne maîtrisant pas le français ne seront pas inclus.

- **Traitements**
- les sujets suivis en thérapie familiale avant ou pendant l'hospitalisation ne seront pas inclus.

### Mode de recrutement et Bilan d'inclusion

Une **fiche de screening** permettra la procédure de sélection. Elle sera remplie par l’équipe de recherche sur les données cliniques relatées par le clinicien soignant la patiente **pour toutes les patientes anorexiques hospitalisées dans le service du Pr Jeammet pendant la durée de l’étude.**

Ainsi, les patientes et les familles présentant les critères d’inclusion , en l'absence des critères d'exclusion définis ci-dessus, seront sollicitées, ainsi que leurs parents, pour participer à cette recherche. Ils seront alors systématiquement reçus par un membre de l’équipe de recherche. Le bilan d’inclusion consistera en un ou plusieurs entretiens cliniques durant lesquels la nature et les modalités de la recherche leurs seront expliqués. Ils resteront libres de donner leur consentement, selon les termes de la déclaration d'Helsinki. Un consentement écrit sera rempli par la patiente et ses parents à la suite d'une explication claire et adaptée du protocole.

Le nombre et la nature des refus de participation sera noté. Les patients et les parents refusant de participer à l’étude seront néanmoins évalués initialement et tous les 6 mois s’ils l’acceptent.

### Non respect du protocole

- **Inclus à tort**

Ils devront être évités en veillant au respect des critères d’inclusion et d’exclusion. Néanmoins en cas de sujets inclus à tort, leur exclusion devra être mentionnée.

- **Non respect du protocole**

Ils doivent être limités et si ils sont constatés, ils doivent être décrits. L’adjonction d’une thérapie familiale à une famille ayant été assignée au groupe sans thérapie familiale n’entraînera pas d’exclusion. Inversement l’arrêt de cette thérapie aura la même conséquence.

- **Perdus de vue**

Ils doivent être limités par un rappel systématique par le thérapeute individuel, le consultant, et le thérapeute familial des sujets arrêtant leur suivi**.** En cas d'abandon le motif doit être précisé, et le sujet tout de même recontacté pour lui proposer le suivi.

## Nombre prévu de personnes et justification

Nous pensons recruter **60 patients et 60 familles**

Comme nous l’avons expliqué au début de cet exposé le nombre minimal de sujets nécessaires à une étude dépend pour chaque critère de jugement défini de la force de l'effet attendu, de la variance et des risques d’erreur alpha et bêta. Si on s’intéresse aux données de la littérature, seule l’étude de Russell a pu mettre en évidence une efficacité de la thérapie familiale. Pour cela il utilisait l’échelle de Morgan et Russell que nous utiliserons aussi. Les sujets que nous retiendrons correspondent au premier groupe de cette étude. Russell et al ont inclus dans leur étude 21 sujets de ce groupe, soit **10 à 11 sujets** par groupe de comparaison (Voir tableau 1). Ils ont réussi à mettre en évidence un effet de la thérapie familiale mesuré sur l’échelle de Morgan et Russell en faisant des regroupements de modalité sur l’échelle, avec un test de Fisher (p<0,002 si les groupes « bon et intermédiaire » sont réunis et p<0,02 si les groupes « mauvais et intermédiaire » sont réunis).

**Tableau 1 : Thérapie familiale versus thérapie individuelle à un an de suivi : effet sur le devenir général**

|  | *Catégories de devenir* | | | |  |
| --- | --- | --- | --- | --- | --- |
| *Thérapie* | *Total* | *Bon* | *intermédiaire* | *mauvais* | *p( test de Fisher)* |
| Familiale | 10 | 6 | 3 | 1 | *<0,02 |
| Individuelle | 11 | 1 | 1 | 9 | **<0,002 |

Compte-tenu de la méthodologie les résultats ne peuvent être extrapolés à notre étude pour en estimer le nombre de sujets nécessaires. En effet, les traitements comparés ne sont pas les mêmes. Intuitivement, on peut penser que la différence observée entre les deux groupes dans notre étude sera probablement moindre, car les deux groupes recevront le même traitement, reconnu efficace pour leur anorexie. En conséquence nos effectifs seront peut être plus importants .

**Cette différence n’est pas à ce jour connue, la littérature ne peut nous la fournir**. Nous réalisons une **étude préliminaire qui doit nous permettre d’observer la différence** afin de déterminer la taille de l’échantillon final utile à notre étude. **Cela déterminera la durée de l’étude compte tenu des autres paramètres que sont le recrutement (connu), et les taux d’acceptation et de compliance qui restent tous deux à déterminer.**

**En première approximation, et compte tenu des données exposées ci-dessus nous évaluons à 60 patients et 60 familles l’échantillon nécessaire, ce qui sera réévalué en cours d’étude au vu des résultats de l’étude préliminaire.**

## Durée de participation de chaque personne ayant accepté de participer à la recherche

Chaque sujet et chaque famille seront donc inclus pour une durée de dix huit mois s’ils l’acceptent.

# Schéma et conduite de la recherche (voir schéma ci-dessous)

Toutes les patientes anorexiques du service et leur famille et se verront proposer la recherche au cours d’un entretien d’inclusion initial. Les sujets qui l’acceptent seront **tous évalués qu’ils soient inclus ou non.**

## Suivi des patients :

Les sujets inclus seront **évalués à l’entrée dans l’étude et tous les 6 mois** jusqu’à la fin du traitement par thérapie familiale. Cette durée est de dix huit mois, c’est celle classiquement proposée dans le service. Elle correspond à celles décrites dans la littérature (Voir revue ci dessus : 12 à 18 mois)

**Patientes anorexiques**

**Hospitalisées**

**Age début ≤ 18 ans**

**Durée évolution ≤ 3 ans**

**Proposition de participation**

**à l’étude à la sortie d’hospitalisation**

**acceptation**

**Refus**

**Randomisation**

**1ère évaluation à la sortie 1ère évaluation à la sortie**

**Traitement Traitement**

**Classique Classique**

**+ Thérapie Familiale**

**Puis tous les**

**6 mois durant 18 mois**

**Réévaluation Réévaluation**

**Plan de l’Etude**

**sur l’évaluation de l’efficacité de la thérapie familiale**

**dans l’anorexie mentale**

### chronologie et contenu des visites

Les patientes seront vu d’une part pour **suivre leur traitement** (suivi par un consultant avec ou sans thérapie familiale) selon un rythme et des modalités définies **au chapitre 7 en détails**.

D’autre part elles seront vues ainsi que leur famille **être évalués lors d’entretiens de recherche** selon le plan suivant :

#### Recueil des données

##### Par qui

Les membres de l’équipe de recherche formés à l’utilisation de ce questionnaire (deux ou trois personnes pour limiter la variation intercotateurs) rencontreront les patients et leur famille.

##### Qui

Les sujets et les parents seront évalués individuellement.

##### Quand

Ils seront évalués :

- à l’entrée dans l’étude,

puis tous les six mois plus tard

##### Comment

Lors d’entretiens menés par un membre de l’équipe de recherche, grâce au questionnaire défini au chapitre méthodologie (chapitre 8)à la consultation du service. Les sujets seront reconvoqués par téléphone et/ou par courrier.

#### Actes thérapeutiques

Les patients recevront tous un traitement classique pour leur anorexie mentale. La moitié d’entre eux se verra prescrire en plus **une thérapie familiale systémique entant dans le cadre de la recherche**. (Voir chapitre 7 pour le détail de la thérapeutique).

### actes

Les actes réalisés sont des consultations de psychiatrie et de thérapie familiale.

### lieu de réalisation des examens

Toutes ces évaluations seront réalisées dans le service du Pr JEAMMET.

## Durée totale prévisionnelle de la recherche

La **durée** de cette recherche **couverte par la présente demande de financement est de trois ans.**

La durée totale est de trois ans et demi (six mois sont exclusivement couverts par le CRC et concernent l’étude préliminaire)

# Procédures évaluées

Tous les patientes inclues le seront après une hospitalisation pour anorexie mentale. Puis elles seront toute randomisées pour l’attribution de la thérapie familiale et auront toute des traitements associés composant une prise en charge classique. Nous allons donc décrire successivement le traitement en hospitalier, la thérapie familiale et les traitement associés.

## Traitement en hospitalier

La prise en charge des patientes anorexiques dans notre service est individualisée pour chaque sujet, mais répond néanmoins à des règles communes. Les sujets sortent dans un état somatique et psychologique permettant la poursuite des soins à l’extérieur. On peut le schématiser comme suit : le traitement des sujets présentant une anorexie mentale peut s'envisager en hospitalier en fonction de la gravité somatique (maigreur importante avec retentissement somatique) et/ou psychiatrique et psychique (dépression, tentative de suicide, repli important) et en fonction de la situation (Agman et al, 1994).

L'approche de ce trouble ne se conçoit que dans le cadre d'une collaboration multidisciplinaire (infirmiers, psychologues, psychiatres, généralistes, nutritionnistes, gynécologues, rhumatologues, diététiciennes, ergothérapeutes, assistantes sociales...) Un suivi psychiatrique est à notre sens indispensable et doit être complété par un suivi médical régulier.

La prise en charge devra prendre en compte la symptomatologie alimentaire elle-même, les conséquences de la dénutrition, les troubles psychologiques du sujet, les interactions familiales autour de l'anorexie. La prise en compte de la famille est fondamentale en tant qu'alliée thérapeutique, dans le but d'évaluer les interactions éventuellement pathogènes. Cette prise en charge est faite systématiquement par des psychiatres.

Dans tous les cas, on cherchera à diversifier les investissements des sujets et à utiliser des médiations rendant tolérables les relations instaurées. On utilisera pour cela un contrat de soins ambulatoire ou hospitalier (Jeammet, 1984).

Un contrat d'hospitalisation (Jeammet 1984) est mis en place entre l'équipe, la famille et le patient. Ce contrat va définir un temps de séparation entre la famille et la patiente. Il est construit autour de deux axes : le poids de sortie définitive permettant au patient de quitter le service et le poids intermédiaire appelé "poids de levée de séparation". Ce poids de levée de séparation est ainsi nommé car il met fin à la première partie d'hospitalisation qui se déroule en séparation d'avec le milieu de vie du patient (parents, amis, scolarité...). La deuxième phase de l’hospitalisation est une reprise progressive des investissements et de la vie extérieure ( week-end en famille, école à hôpital...). On parle de séparation et non d’isolement dans la mesure où le sujet vit dans le service une vie institutionnelle avec les soignants et les autres soignés. Il y a des activités de groupe et d'échange qui sont indispensables à l'épanouissement des patients. La patiente réapprend le plaisir de l'échange presque à son insu.

A cela s'ajoutent des entretiens réguliers, des réunions soignants soignés, un groupe de parole une fois par semaine pour les sujets sortis de la phase de séparation, un groupe de parole mensuel pour les parents ; les parents sont reçus régulièrement en entretien familial sans leur fille pendant la phase de séparation, puis avec elle.

Un médecin généraliste est attaché au service et suit ces patientes sur le plan somatique. Un réanimateur peut intervenir en cas d'urgence. Les patientes sont pesées une fois par semaine voire plus (2 à 3 fois) si leur état somatique est inquiétant.

La renutrition est, sauf cas d'extrême urgence, proposée d'emblée par des repas normaux en salle à manger commune, et en service libre. Après une évaluation de durée variable, selon l'état de la patiente, on propose des mesures d'aménagement. Les repas peuvent être proposés sur plateau dont les quantités sont préparées, puis avec une stimulation accrue des soignants. Des repas en chambre avec accompagnement d'un soignant peuvent être mis en place. En dernier recours, on peut utiliser une nutrition par sonde oeso-gastrique. Les cas extrêmes de dénutrition sont adressés en réanimation. Les repas sont suivis de périodes de repos en chambre, de durée variable en fonction de l'état physique de la patiente. Bien entendu s’ajoutent à cela des soins corporels : prévention des complications de décubitus avec massage, matelas anti-escarres, mobilisation, soins des dents, etc...

Aucun traitement médicamenteux n'est spécifique de l'anorexie. On peut utiliser en appoint des anxiolytiques en cas d'anxiété, voire des antidépresseurs en cas de dépression, mais rarement d’emblée. Ceci est d'autant plus important qu'il existe des risques d'effets secondaires graves des traitements proposés pour l'anxiété ou la dépression en cas de dénutrition.

La durée de l'hospitalisation est en moyenne de trois mois, mais peut aller jusqu’à un an. Une hospitalisation trop rapide ne permet pas un travail psychologique satisfaisant. Il faut parfois plusieurs hospitalisations. L’intervention d'une assistante sociale est souvent indispensable pour mettre en place une scolarité aménagée ou non, et permettre un retour à la vie sociale.

L'hospitalisation débouche toujours sur un suivi ambulatoire de plusieurs mois, voire plusieurs années. Parfois la poursuite intensive des soins est nécessaire et les patientes intègrent un internat médico-social permettant une reprise de scolarité avec un soutien psychologique intense.

## Traitement étudié : la thérapie familiale

### Modalités pratiques

La thérapie familiale sera dispensée à l'Institut Mutualiste Montsouris par l'équipe des thérapeutes du service. Elle commencera à la sortie d’une hospitalisation pour anorexie mentale. La thérapie familiale **est d’orientation théorique systémique**. **Les** **séances sont dispensées à la fréquence de une toutes les trois à quatre semaines**, pour une durée de 18 mois (prolongeable), soit 24 séances approximativement. **Elles** **durent une heure et trente minutes**.

Cette thérapie concerne **les patientes, leur mère, leur père, et leurs frères et soeurs de plus de 6 ans.** La thérapie familiale systémique sera réalisée conformément aux pratiques classiques par des thérapeutes n’étant pas impliqués dans la prise en charge personnelle de la patiente, (Dally et al, 1981). Elle sera pratiquée par **un binôme de thérapeutes** familiales expérimentés en co-thérapie : une psychiatre psychothérapeute et une psychologue. Les entretiens sont libre avec des interventions actives des thérapeutes tout au long de la séance. En fin de séance les thérapeutes délivrent un commentaire global. .

### Généralités

**L'approche familiale s'avère complémentaire des autres modalités de prise en charge**. Les prises en charge familiales ont paru nécessaires à instaurer pour les anorexiques du fait de l'origine de la demande thérapeutique (les parents le plus généralement demandent les soins, les anorexiques elles‑mêmes déniant tout problème), de l'implication très active de la famille chez de jeunes patientes (l'anorexie mentale se manifeste souvent au début de l'adolescence), la patiente vivant en général encore complètement dans sa famille.

Le modèle thérapeutique initial très codifié a été proposé par S. Minichin pour les anorexiques et leurs familles (1978) . Cependant, l'expérience clinique appelle une grande souplesse par rapport à ce cadre formel. Nous pensons également que ce sont les conceptions des valeurs fonctionnelles du symptôme dans la famille qui sont importantes. Minuchin proposait une thérapie familiale à partir de l'hospitalisation de la patiente anorexique, ce modèle servant en quelque sorte de base à de nombreuses variantes. Nous avons fait le choix de réaliser des entretiens familiaux en cours d'hospitalisation, et de débuter une thérapie familiale à la sortie de l’hospitalisation comme l'a fait l’équipe de Russell (1987)

Les thérapies familiales tirent leur originalité de **l'examen de la souffrance d'un individu d'un point de vue autre qu'intrapsychique ou nosographique, c'est-à-dire d'un point de vue intersubjectif et relationnel**. C’est la famille et les relations au sein de celle-ci qui sont prises en compte dans la thérapie et non plus l’individu.

Les **symptômes s'expriment à l'intérieur d'un groupe** spécifique, premier lieu de sens pour l'individu : **la famille**, prise comme une unité fonctionnelle composée de personnes dont les relations sont intenses (donc pas nécessairement la famille nucléaire ou celle dont les membres vivent sous le même toit).

Le groupe familial ne se réduit pas à la seule articulation des fonctionnements individuels des individus qui la composent. Il évolue constamment selon une logique interne, un jeu ou chacun assure des fonctions destinées à perpétuer l'existence du groupe, tout en essayant de promouvoir son intentionnalité propre, de délimiter son espace personnel, d'accroître son degré d'autonomie.

Cette **tension entre les besoins d'appartenance au groupe et les besoins d'individuation**, entre le degré de liberté individuelle et la nécessité de maintenir la cohésion du groupe est **en perpétuel remaniement,** au fur et à mesure que la famille poursuit son cycle vital.

Le groupe familial ne se contente pas de réagir à la réalité psychique du patient mais s'organise en l'incluant.

**Sans nier la souffrance personnelle, ses aspects intrapsychiques et métapsychologiques, ni les aspects somatiques et biologiques de la pathologie, l'accent est mis sur les interactions fonctionnant autour du symptôme.** **L'implication relationnelle et le symptôme prennent un sens particulier, à un moment donné de la vie familiale où la dynamique du groupe dans son ensemble et ses rapports avec ses singularités sont particulièrement sensibles.**

**Il s'agit donc d'élaborer en permanence les relations, au carrefour de l'interaction et de la subjectivité, relations considérées comme refontes incessantes des limites et des identités.**

La définition de la relation est dans ce contexte un processus dynamique, s'appuyant sur l'oscillation entre interface et intrapsychique, entre appartenance et séparation, incluant activement le tiers participant à cette définition, tiers qui n'est pas un observateur ayant un point de vue neutre et objectif sur cette relation.

**L'adolescence peut être considérée comme un de ces moments à la fois désorganisateur pour le groupe familial, et créateur de nouveaux échanges** et de modifications des liens pour chacun. La survenue du **symptôme anorexique** peut être compris d'une part comme **un** **indicateur et un facteur de crise familiale,** d'autre part il joue **un rôle de renforcement de la stabilité du système** **familial** (ce symptôme anorexique étant une tentative de maîtrise induit par l’adolescence, entraînant une rigidification des transactions entre ses membres qui ne peuvent tolérer cette désorganisation transitoire).

**Dans ces familles les individus sont peu différenciés, l'autonomie et les compétences personnelles ne sont pas reconnues, les distances entre individus ne sont pas respectées. L'enchevêtrement entre les générations se traduit par une perméabilité des frontières verticales.**

De même ces familles partagent **un niveau de tension émotionnelle intense** ; **ces** **émotions sont peu différenciées**, peu symbolisées, leur perception n'est pas reconnue ; les réactions et le comportement sont régulés par les fluctuations émotionnelles pour lesquelles la parole circule difficilement.

On accordera en conséquence de l'importance à l'aspect non verbal des relations, à l'expression analogique dans la famille : attitudes, place des uns par rapport aux autres, expression des affects...

La famille en général évolue donc avec des émotions partagées et peu différenciées, construit son histoire en intégrant et en transformant ce qui a été transmis dans la succession des générations. Les familles de sujets anorexiques ont des croyances implicites et rigides qui concernent souvent le rôle des filles dans la famille (White, 1983). **La perspective transgénérationnelle** est donc essentielle au mode de lecture systémique de la famille. Elle représente l'axe diachronique, pendant indissociable de l'ici et maintenant de la famille que nous rencontrons.

A travers les générations, la filiation, l'histoire des familles d'origine de chacun des parents sont transmises par injonctions, dettes, obligations réalisées ou non qui constituent des loyautés, c'est-à-dire des nécessités pour chaque individu de répondre aux attentes du groupe familial.

Dans ces familles très rigides, les changements vécus comme dangereux favorisent l'émergence de symptômes chez un adolescent. Celui-ci est souvent lié par des loyautés invisibles à la famille d'origine, à l'un de ses membres, parfois mis au banc de la famille à la suite d'une rupture sans parole plus ou moins brutale d'avec un ancêtre, sans que les émotions liées à la perte puissent être partagées.

### Axes de traitement

Si dans l'absolu il s'agit de conduire la famille à un point où le symptôme n'est plus nécessaire, il nous paraît plus important d'essayer d'amener une plus grande différenciation des individus dans la famille, de permettre à chacun d'être plus en contact avec ses émotions et ses affects et de mieux aménager les relations (distance-proximité) à l'intérieur comme à l'extérieur de la famille.

Pour cela il nous semble indispensable de travailler sur deux axes (diachronique et synchronique), c'est‑à‑dire à la fois sur le passé, le présent et le futur :

- **Le passé** : la construction de l'arbre généalogique, la mise à jour des mythes familiaux, l'exploration des loyautés et des pseudo secrets, l'histoire du couple parental vont permettre de faire des hypothèses sur la dynamique transgénérationnelle propre à chaque famille.
- **Le présent** : les conceptions du symptôme (origine, causes...) de chaque membre de la famille, les modes de communication verbale et non verbale, les alliances, les coalitions, le procédé du bouc émissaire... nous conduisent à une évaluation de la structure familiale (avec ses frontières, ses liens aux familles d'origine) et du processus évolutif de la famille.
- **Le futur :** il nous apparaît nécessaire que chacun exprime ses attentes en ce qui concerne la conduite alimentaire symptomatique, donne son opinion sur les traitements mis en route, imagine l'impact des changements dans la famille et sur chacun de ses membres, ce qui nous donne une idée sur les aptitudes au changement des individus et du système dans son entier.

**Proposer un cadre thérapeutique pour le groupe familial c'est reconnaître le débordement de ses capacités adaptatives, la souffrance des individus dans ce groupe, la difficulté actuelle à vivre ensemble avec la patiente et ce qu'elle fait éventuellement subir à son entourage. Mais c'est aussi reconnaître la compétence de la famille dans ses modes de perception, de compréhension et d'action...(soulagement et intérêt de la fratrie lorsqu'ils sont associés par exemple).**

La demande initiale et explicite de la famille est l'effacement du symptôme et le retour à l'état antérieur d'équilibre familial, ou à son illusion, fantasme dont tous ont la nostalgie.

Un des premiers objectifs du traitement familial est de faire alliance avec cette famille, porteuse d'une demande contradictoire. Le symptôme est donc le point d'appui de cette affiliation, il permet aux thérapeutes d'entrer dans la famille.

S'affilier à la famille est la première étape nécessaire à la construction d'un système thérapeutique qui devra être évolutif et créatif, le temps suffisant pour que les thérapeutes comme la famille puissent mutuellement se dégager, afin que celle-ci retrouve ses propres capacités de régulation et d'invention.

En séance les thérapeutes s'intéressent à toutes les modalités de communication (verbale et analogique). Il s'agit non seulement de susciter et de soutenir la parole, mais aussi de repérer les conditions dans lesquelles celle-ci est énoncée, la façon de réagir des autres membres de la famille, de se laisser aller à éprouver les émotions qui surgissent.

Comme tout travail thérapeutique, la thérapie familiale nécessite, de la part des thérapeutes, un incessant aller et retour entre penser et ressentir. Cependant elle nous met en contact avec des systèmes d'éprouvés très complexes, des organisations préverbales qui renvoient volontiers au vécu de l'adolescent qui agit un ressenti et cherche éventuellement, dans les cas les plus favorables, à le comprendre dans l'après coup.

Le cadre thérapeutique doit offrir un contenant, limitant l'excitation, organisant les échanges, sans laisser l'association libre ou les projections s'exprimer sous une forme trop crue.

La connotation positive est d'une grande utilité dans le processus thérapeutique. Le symptôme est une création du système familial pour tenter de résoudre une crise, c'est-à-dire une solution de compromis visant à dépasser ses contradictions. Le thérapeute familial peut faire part à la famille de cette conviction, conférer au symptôme un sens positif par rapport à la position du groupe familial dans une formulation acceptable pour la famille. La connotation positive n'a d'intérêt que dans l'aspect singulier d'une famille, ce qui indique la reconnaissance par les thérapeutes de l'identité de la famille.

En conséquence, le processus thérapeutique ne vise pas un savoir de plus en plus complet des thérapeutes sur la famille par l'accumulation d'informations, mais plutôt la mise en place d'un espace commun où thérapeute et famille sont liés mais différenciés. S'appuyant sur une résonance mutuelle, il va être possible d'utiliser des métaphores qui renvoient au sens du symptôme dans le fonctionnement actuel de la famille, dans ses liens transgénérationnels, dans la place et le rôle de chacun à l'intérieur du groupe.

Par leur action les thérapeutes tentent d'amener une plus grande autonomie des individus dans la famille, permettent à chacun d'être plus en contact avec ses émotions et ses affects et de mieux les différencier, d'aménager les relations et les limites (rapprochement-éloignement) à l'intérieur comme à l'extérieur du système (Caillé, 1978).

## Traitements ambulatoire après hospitalisation

### Pratique classique

Un suivi **ambulatoire** bifocal est mis en place à la sortie de l'hospitalisation, associant un suivi par un psychiatre consultant référent de la prise en charge et souvent, un psychothérapeute. Cependant des variations peuvent survenir dans la mise en place de ce suivi. Ainsi l’intensité de la prise en charge ambulatoire sera variable en fonction du degré d'évolution de l'anorexie. Cette prise en charge doit être coordonnée par un consultant référent dont le rôle est tenu dans le service par un psychiatre. La patiente est reçue en consultation par un psychiatre assurant le suivi global (pesée sur place), qui prescrit les interventions nécessaires aux soins.

Une prise en charge des parents au cours d'entretiens avec la patiente est indispensable. La fréquence des entretiens est de une fois par mois. L'ambivalence des parents face aux soignants risque d'être dommageable aux soins, et cette consultation psychiatrique peut permettre la coordination nécessaire au bon fonctionnement thérapeutique. Bien souvent les parents se sentent culpabilisés par le corps médical ; revaloriser leur position est indispensable.

Dans un deuxième temps, d'emblée pour certains sujets quand cela est possible ,un travail psychothérapique individuel plus élaboré est mis en place. Les psychothérapies proposées dans le service sont d'inspiration analytique.

Un suivi somatique, soit par le médecin de famille, soit par le généraliste du service sera généralement mis en place assurant entre autre une prévention des complications osseuses, des longues aménorrhées et de la dénutrition en collaboration avec des collègues gynécologues, rhumatologues et endocrinologues si nécessaire.

### Adaptations pour l’étude

On ne modifiera pas ces aspects de la prise en charge sauf pour un point. En effet dans un souci de standardisation et d’homogénéisation des pratiques les consultants suivant les patients en post-hospitalisation seront recrutés **dans notre** **équipe**. Les traitements associés seront décrits pour en tenir compte dans l’analyse des données, en particulier pour la thérapie individuelle.

# Critères d’évaluation : variables mesurées et méthodologie

## Critères de jugement

### Critères de jugement principaux

L'efficacité du protocole de soins sera apprécié sur les facteurs classiquement définis dans la littérature, qui sont **les témoins de l’amélioration clinique de la patiente** : (Voir méthode pour les instruments cités ci-dessous)

- **amélioration du fonctionnement global** (psychique, alimentaire, relationnel, social, sexuel) évalué par le score global de l’échelle de Morgan et Russell
- **amélioration clinique symptomatique au niveau du poids et des attitudes alimentaires :**
- la normalisation du poids et la durée du maintien de ce poids évalué par le BMI (Body Mass index, Beumont, 1988)
- la reprise des règles et la régularité du cycle menstruel,
- l'évolution du score à l’autoquestionnaire « eating disorders inventory » (EDI)
- **diminution des hospitalisations et des rechutes,**
- **facteurs psycho-sociaux** : une augmentation de l’autonomie et de l’adaptation sociale évaluées par le score à l’échelle sociale de Morgan et Russell, l’autoquestionnaire d’adaptation sociale (Social adjutsment scale self report) (Weissman MM, Bohwell S., 1976) et l’échelle de Groningen: "Groningen Social Disability Schedule seconde version" (Wiersma 1988a et b).
- symptomatologie anxio-dépressive évaluée par une échelle de psychopathologie générale SCL 90-R, l'échelle de phobie sociale de Liebowitz, le questionnaire de Beck, l'échelle de Hamilton.
- Doivent aussi être mesurées les modifications des relations familiales obtenues, et l'amélioration de la communication pour les mettre en relation avec l’évolution clinique à l'aide des instruments suivants :
- **Family Adaptability and Cohesion Scale (FACES III)**
- **Un hétéro-questionnaire à définir**

### Critères de jugement annexes

L’efficacité des soins sera évaluée par le devenir des patientes. Ce devenir est influencé par des **facteurs pronostiques reconnus**, présents chez le sujet ou sa famille. Ils doivent donc être évalués. Ce sont, outre la durée d’évolution des troubles et l’âge de début

- la présence de **syndromes dépressifs ou anxieux** personnels et familiaux évalué par le MINI,
- l’existence de **symptômes boulimiques**,
- un **poids** bas au début du traitement,
- des **symptômes dépressifs**,
- et des symptômes **obsessionnels et compulsifs**,
- on évaluera donc en plus de ce qui est précédemment décrit :
- l'abus de substance, les pathologies psycho-somatiques et les antécédents de trouble du comportement alimentaire, évalué par le MINI et
- la qualité des liens entre le patient et ses parents durant la petite enfance, évaluée par le PBI Parental Bounding Instrument) .

### Actes réalisés

Pour recueillir ces données **les patients et leurs familles devront être évalués lors d’entretiens de recherche** selon le plan suivant :

#### Recueil des données

##### Par qui

Les membres de l’équipe de recherche formés à l’utilisation de ce questionnaire (deux ou trois personnes pour limiter la variation intercotateurs) rencontreront les patients et leur famille.

##### Qui

Les sujets et les parents seront évalués individuellement.

##### Quand

Ils seront évalués :

- à l’entrée dans l’étude,

puis tous les six mois (voir ci-dessous).

##### Comment

Lors d’entretiens menés par un membre de l’équipe de recherche, grâce au questionnaire défini au chapitre méthode, (voir tableau ci-dessous) à la consultation du service. Les sujets seront reconvoqués par téléphone et/ou par courrier.

|  | **Patient** | | **Parents** | |
| --- | --- | --- | --- | --- |
| **Auto-évaluation** | **évaluation initiale** | **évaluation à 6,12,18,mois** | **évaluation initiale** | **évaluation à 6,12,18 mois** |
| **Données cliniques** | + | + | + | + |
| **PBI** | + |  | + |  |
| **Beck** | + | + | + |  |
| **Blatt** | + | + | + |  |
| **EDI** | + | + | + | + |
| **SAS-SR** | + | + | + | + |
| **TAS 20** | + | + | + | + |
| **SCL-90-R** | + | + | + |  |
| **FACES** | + | + | + | + |
|  |  | |  | |
|  | **Patient** | | **Parents** | |
| **Hétéro-évaluation** | **évaluation initiale** | **évaluation à 6,12,18 mois** | **évaluation initiale** | **évaluation à 6,12,18 mois** |
| **Données cliniques** | + |  | + |  |
| **Psychosomatiques** | + | + | + | + |
| **GRONINGEN** | + | + | + | + |
| **HAMILTON** | + | + | + |  |
| **LIEBOWIETZ** | + | + | + |  |
| **MINI** | + | + | + |  |
| **MORGAN ET RUSSELL** | + | + | + |  |
| **SADS (AS)** | + |  | + |  |
| **Y-BOCS** | + | + | + |  |
| **Relations familiales** | + | + | + | + |

## Méthodes et techniques utilisées : instruments composants le questionnaire et l’autoquestionnaire

### Diagnostics

Le diagnostic d’anorexie mentale sera émis à partir des critères du DSM IV.

Pour évaluer les antécédents familiaux de troubles anxieux, de troubles thymiques, d'abus de substances et de troubles du comportement alimentaire nous utiliserons un hétéroquestionnaire appelé Mini International Neuropsychiatric Interview (MINI). C'est un entretien diagnostique structuré permettant d'explorer de façon standardisée chacun des critères nécessaires à l'établissement des principaux diagnostics psychiatriques tels qu'ils sont définis dans le DSM-IV. (Lecrubier 1995)

Pour les antécédents d'angoisse de séparation : la SADS-LA(R).

Nous utiliserons la section " angoisse de séparation" de la SADS-LA (R) (version révisée de la "Schedule for Affective Disorders and Schizophrénia modified for the study of anxiety disorders"), version modifiée pour les troubles anxieux, traduite en français et validée (M. Leboyer, M. Teherani, JP. Lépine).

La SADS-LA (R) est un entretien semi-structuré permettant de poser un diagnostic rétrospectif d'angoisse de séparation selon les critères DSM-III-R. Cet instrument introduit une nuance dans ce diagnostic en qualifiant ce trouble soit de certain (trois manifestations certaines, au minimum, parmi les neuf contribuant au diagnostic), soit de probable (deux manifestations certaines et une probable, minimum, parmi les neuf contribuant au diagnostic). Nous attendons la version DSM-IV.

### Symptômes et attitudes alimentaires :

#### Eléments cliniques

Seront rercherchés :

- l’existence de symptômes boulimiques,
- le poids au début du traitement,
- la durée d’évolution des troubles et l’âge de début,
- l’évolution du poids qui sera évaluée en calculant le BMI (Beumont, 1988),
- la reprise des règles et la régularité du cycle menstruel,
- le nombre des hospitalisations et des rechutes,

#### L'inventaire des troubles des conduites alimentaires (Eating Disorders Inventory, EDI)

Il s'agit d'un autoquestionnaire pour les troubles de conduites alimentaires construit par Garner et al. (1983) qui, selon Vandereycken et Meermann (1984) "est actuellement le meilleur instrument disponible pour évaluer les TCA, du fait de sa validité". Ce questionnaire multidimensionnel évalue différentes caractéristiques psychologiques et comportementales des patientes anorexiques et boulimiques.

La construction des échelles s'est appuyée sur un certain nombre d'hypothèses théoriques. Il comporte 64 items qui se répartissent en 8 échelles de la manière suivante :

1. Désir de minceur
2. Boulimie
3. Insatisfaction concernant le corps
4. Inefficacité
5. Perfectionnisme
6. Méfiance dans les relations interpersonnelles
7. Préoccupations par rapport aux perceptions intéroceptives
8. Craintes par rapport à la maturité

Les trois premières échelles évaluent le trouble des TCA et l'image du corps. Les autres échelles évaluent les aspects psychopathologiques et psychogénétiques des troubles. Les réponses sont cotées sur une échelle à six points : toujours, généralement, souvent, parfois, rarement, jamais.

La validité et la fidélité de chacune des échelles de l'EDI varient entre 0,82 et 0,90. Certaines des échelles font la distinction entre les patientes souffrant d'anorexie, celles souffrant de boulimie ou d'autres troubles des conduites alimentaires. Garner et al. (1983) ont donné les scores moyens pour un large échantillon de patientes anorexiques (N=129) et un groupe contrôle de 770 femmes.

#### Le questionnaire de Morgan et Russell

Il s'agit d'un questionnaire construit pour évaluer l'évolution au long cours. Les échelles de Morgan et Russell (basées sur les travaux de Morgan et Hayward 1988) permettent d'évaluer les principaux aspects de l'anorexie sur une période de six mois. La procédure d'évaluation comporte 14 sous-échelles réparties en 5 groupes :

A : Prise alimentaire

A-1 Restriction de la prise alimentaire

A-2 Préoccupations autour de l'image du corps

A-3 Poids corporel

B : Etat menstruel

C : Etat mental

D : Etat psycho-sexuel

D-1 Attitude par rapport à la sexualité

D-2 Objectifs dans le domaine sexuel

D-3 Comportement sexuel désinhibé

D-4 Attitude par rapport aux règles

E : Etat socio-économique

E-1 Relation avec la famille

E-2 Emancipation de la famille

E-3 Contacts sociaux en dehors de la famille

E-4 Activités sociales en dehors de la famille

E-5 Travail

Le score moyen final est calculé à partir de la moyenne des scores aux sous-échelles. Morgan et Hayward (1988) ont fourni les résultats d'une étude de suivi portant sur 40 patientes anorexiques chroniques et sur 75 anorexiques subcliniques. Les 14 sous-échelles étaient significativement corrélées avec le poids corporel (sous-échelle A-3) et le cycle menstruel (sous-échelle B), qui sont considérées comme de bons critères pronostiques de l'évolution.

### Les facteurs psycho-sociaux :

#### Le questionnaire de Morgan et Russell

Voir ci-dessus

#### Echelle de GRONINGEN: "Groningen Social Disability Schedule seconde version"

Cette échelle est utilisée avec un entretien semi-structuré pour évaluer l’adaptation sociale (Wiersma 1988a et b). Nous utilisons uniquement les sections "rôle occupationnel" (traduction française par A. Cariat et Y. Lecrubier) et "rôle social" (traduction française par N. Godart et M. Flament).

Le rôle social évalue les relations avec les amis et les connaissances à partir de deux dimensions : la qualité des contacts d'une part, et leur fréquence et étendue d'autre part.

Le rôle occupationnel évalue l'adaptation aux activités quotidiennes à partir de quatre dimensions (Adaptation à la routine quotidienne, Performances, Contacts avec autrui liés au rôle, Activités quotidiennes).

Les dimensions sont évaluées chacune séparément (0 = pas d'inadaptation, 1 = inadaptation légère, 2 = inadaptation moyenne, 3 = inadaptation sévère). Le score global est obtenu en prenant la plus élevée de ses dimensions. Cependant en cas de grande divergence entre ces notes le cotateur peut adapter la note globale en fonction de son impression clinique.

L'évaluation concerne les quatre semaines précédentes et, pour les anorexiques hospitalisées, les quatre semaines précédant leur hospitalisation.

#### Le questionnaire d’adaptation sociale (Social adjutsment scale self report : SAS-SR)

Le questionnaire d’adaptation sociale (Social adjutsment scale self report) (Weissman MM, Bohwell S., 1976) est une version par auto-évaluation de l’échelle d’adaptation sociale (SAS : Social adjustment scale). Ses 54 questions portent sur six secteurs de fonctionnement social :

- travail
- vie sociale et loisirs
- famille
- relations conjugales
- relations avec les enfants
- unité de la famille

Sont appréciés à la fois le comportement objectif du sujet, ses sentiments, et son degré de satisfaction.

D’une administration simple, il permet habituellement une surveillance clinique régulière des patients, et surtout d’évaluer les effets au long cours de tous types de traitements, en particulier psychothérapique et institutionnels.

### La psychopathologie générale : le SCL-90-R

L'évaluation de la **psychopathologie générale** sera réalisée à l'aide du **SCL-90-R** (L.R. Derogatis 1977)

Ce questionnaire d'auto-évaluation des symptômes est un auto-questionnaire utilisé pour évaluer une variété de symptômes gênants. Les 90 items sont divisés en 9 sous-catégories factorielles :

1. Somatisation

2. Obsessionnelle-compulsive

3. Sensitivité

4. Dépression

5. Anxiété

6. Hostilité

7. Anxiété phobique

8. Idéation paranoïde

9. Symptômes d'allure psychotique.

Les réponses concernant la présence des symptômes variés sont codés selon une échelle en 5 points selon la gravité : pas du tout, un peu, considérablement, beaucoup, beaucoup trop.

La consistance interne de ces 9 sous-catégories factorielles va de 0,80 pour l'idéation paranoïde à 0,90 pour la dépression dans un échantillon de N = 565 sujets.

Des indications concernant la validité peuvent être témoignées par de nombreuses études sur les névrosés, utilisant l'ancienne version (Hopkins, Symptome Check-List, l'auto-questionnaire Hopkins). La validité de construction pourrait être vérifiée pour 8 de ces 9 facteurs sur 1002 patients suivis en ambulatoire (l'exception constitue les symptômes d'allure psychotique).

### La symptomatologie anxio-dépressive

#### L'échelle de phobie sociale de Liebowietz

Nous utiliserons la version française (traduite par Lépine et Cardot 1990). Elle évalue d'une part la peur et l’anxiété sociale et d'autre part l'évitement social par un hétéroquestionnaire (Liebowietz, 1987).

Elle comporte 24 items, divisibles en deux groupes : les expositions sociales de performance (ex : passer un examen) ou de situation (ex : aller à une soirée).

Chaque item est côté deux fois, tout d'abord pour la peur ou l'anxiété suscitée (0 = aucun, 1 = légère, 2 = moyenne, 3 = sévère), ensuite pour l'évitement en découlant (0 = jamais, 1 = occasionnel (0-33%), 2 = fréquent (33-66%), 3 = habituel (67-100%)). Plus les scores sont élevés, plus l’anxiété et l’évitement sont importants. Cela nous permet de quantifier la peur et l'évitement social.

#### L'échelle d'Obsession Compulsion de Yale-Brown (Yale-Brown Obsessive-Compulsive Scale, Y-BOCS)

L'échelle d'obsession compulsion de Yale-Brown (Goodman W.K. et al. 1989 a,b) est un instrument global, synthétique et spécifique pour mesurer l'intensité et la sévérité des symptômes obsessionnels compulsifs. Elle présente l'avantage d'être indépendante du contenu des symptômes et de permettre la comparaison de sujets appartenant à des catégories d'obsessions et de compulsions différentes. Elle apparaît comme l'instrument actuel de référence dans l'évaluation de l'intensité et de la sévérité des obsessions- compulsions.

L'atout principal de la Y-BOCS est de séparer l'évaluation des obsessions (idées) de celle des compulsions (actes rituels), chacune comportant 5 items :

- le temps occupé par les comportements ou les pensées pathologiques ;
- l'interférence avec la vie quotidienne ;
- la détresse ;
- la résistance aux phénomènes pathologiques ;
- le degré de contrôle exercé sur ceux-ci.

Ainsi la Y-BOCS contient 10 items, coté chacun de 0 à 4 permettant d'obtenir une note globale de 0 à 40 et deux notes partielles de 0 à 20 pour les obsessions et les compulsions.

Dans les premières études, on a prouvé la validité des 10 items, avec une excellente fidélité inter-juge du score total et du score de chacun des items (r <.85) et une cohérence interne élevée (coefficient alpha de Cronbach = .89). La validité convergente avec d'autres mesures d'obsessionnalité et la validité discriminante avec la dépression et l’anxiété sont également satisfaisantes. L'échelle de Yale-Brown a montré, entre autre, une sensibilité élevée au changement thérapeutique (Kim S.W. et al. 1994; Woody S.R. et al. 1995).

La traduction et la validation française de l'échelle a été effectuée par Bouvard et al (1992).

#### L’échelle de dépression de Hamilton (Hamilton Depression Rating Scale) Hdrs

C’est selon G.D. Guelfi (1993) l’échelle de dépression la plus utilisée dans le monde depuis sa création en 1960. Cette échelle représente une façon simple d’évaluer de façon quantitative la sévérité d’un état dépressif. Il ne s’agit pas d’un instrument à visée diagnostique.

Pour de nombreux auteurs et dans une première approximation, la note totale de l’échelle de dépression de Hamilton a une validité suffisante (validité courante et discriminante). Sa sensibilité au changement et sa fidélité test-retest, de même que sa fidélité inter-juges, sont élevées (G.D. Guelfi - 1993).

L’échelle est remplie dans les minutes qui suivent un entretien au cours duquel le clinicien cherche à mettre en évidence d’authentiques symptômes dépressifs et non de simples fluctuations thymiques mineures. Des questions doivent être posées au patient dans cette optique. Les données cliniques ainsi recueillies peuvent être complétées si besoin par des informations fournies par l’entourage (Guelfi JD 1993).

Les items de l’échelle sont cotés de 0 à 4. Les cotations de 0 à 4 correspondent respectivement à des symptômes : absents, douteux ou non significatifs, légers, moyens ou importants. La cotation repose sur les données de l’entretien ainsi que sur les phénomènes rapportés par le patient dans le cadre de l’épisode actuel au cours de la semaine écoulée.

Les auteurs français considèrent généralement que les notes proposées dans les études américaines sont nettement trop basses. Une note totale d’au moins 18 est nécessaire ( et non suffisante) pour parler de dépression modérée selon Cottraux (1985).

#### Echelle de Beck : Beck Depressive Inventory

Il s’agit d’un inventaire de mesure de profondeur de la dépression, qui a été développé par Beck à partir de 1962. La forme originale à 21 items incluait tous les symptômes de la constellation dépressive. La forme abrégée n’a retenu que les items les plus fortement corrélés avec la note globale de l’échelle de Beck à 21 items (corrélation supérieure à 90) et ayant également les corrélations maximales avec les évaluations par les cliniciens de l’intensité du syndrome dépressif. Il a été ainsi obtenu un questionnaire de 13 items qui corrélait à 96 avec le score total de l’échelle originale de 21 items, et à 61 avec les évaluations de la dépression effectuées par des cliniciens. (J. Cottraux - 1993). Les 13 items retenus mesurent les symptômes suivants :

- Tristesse
- Pessimisme
- Echec personnel
- Insatisfaction
- Culpabilité
- Dégoût de soi
- Tendances suicidaires
- Retrait social
- Indécision
- Modification négative de l’image de soi
- Difficulté au travail
- Fatigabilité
- Anorexie

On retrouve dans cet ensemble les éléments de la triade cognitive décrite par Beck. Celle-ci comprend des considérations négatives sur soi, le monde extérieur et le futur (item 1 à 8 et item 10). De plus, le ralentissement est appréhendé par les items 9, 11 et 12. L’item 13 représente les troubles somatiques (Cottraux 1993).

Le score total de l’échelle de Beck est corrélé avec le score global de l’échelle de Hamilton (26 items) et avec le score global de l’échelle de ralentissement de Widlöcher. Les deux corrélations ne diffèrent pas significativement (Cottraux et al. 1993).

Chaque item est constitué de 4 phrases correspondant à 4 degrés d’intensité croissante d’un symptôme : de 0 à 3. Dans le dépouillement, il faut tenir compte de la cote la plus forte choisie pour une même série. La note globale est obtenue en additionnant les scores des 13 items. L’étendue de l’échelle va de 0 à 39. Plus la note est élevée, plus le sujet est déprimé (Cottraux et al. 1993).

Elle permet d’alerter le clinicien qui utilise les différents seuils de gravité retenus par Beck et Beamesderfer (1974) :

0 - 4 : pas de dépression

4 - 7 : dépression légère

8 - 15 : dépression modérée

16 et plus : dépression sévère

#### L’échelle de Blatt (DEQ)

Sydney J. Blatt a développé depuis de nombreuses années une réflexion sur les sources primaires de la dépression, s'appuyant à la fois sur des travaux psychanalytiques et cognitivistes. Le « *Depressive Experiences Questionnaire* » a été élaboré dans le souci de permettre une évaluation de l'humeur dépressive à partir d'une approche dimensionnelle fondée sur les expériences de vie, et non sur la symptomatologie.

La question centrale est celle du maintien du contact avec l'objet gratifiant. La perte d'objet réelle ou fantasmée ne peut être assimilée que si le développement des relations d'objet (intériorisation et représentation d'objets) a atteint un niveau suffisant de maturité, après résolution de la crise oedipienne et renoncement aux objets infantiles.

Le questionnaire établit une corrélation de la dépression :

- Soit à des sentiments d'abandon et d'impuissance (helplesness), de faiblesse (weakness) et d'épuisement (depletion), avec peur intense d'être abandonné et lutte désespérée pour maintenir une relation avec des objets gratifiants (*dépression anaclitique*).
- Soit à des sentiments d'autodépréciation (worthlesness), d'autocritique (self-criticism), de honte et de culpabilité, et des difficultés ou une impossibilité à vivre avec des images parentales dures, critiques ou idéalisées (*dépression d'intériorisation*).

Le Depressive Experiences Questionnaire (DEQ) est un autoquestionnaire en cours de traduction optimale et de validation en France (dans le cadre du réseau INSERM « Dépendance », F.Atger, M. Corcos, A. Meilhoc). Il comprend 66 items, chacun ayant trait à un type d'expérience souvent associé au vécu dépressif. Aucun n'appartient au registre symptomatique proprement dit. Les items sont positifs ou négatifs et cotés de l à 7 (l pas du tout d'accord à 7 tout à fait d'accord), 4 étant le point médian*.* L'analyse factorielle a déterminé les trois facteurs orthogonaux suivants :

***Le facteur I Dépendance*** (dépression anaclitique) du DEQ analyse spécifiquement la qualité des relations interpersonnelles, l'angoisse de séparation et d'abandon, les sentiments de solitude et d'impuissance, et les potentialités régressives. Certaines questions portent de plus sur les sentiments de rejet ou d'humiliation, sur la gestion de la colère et de l'agressivité, sur la peur de la perte d'un être cher. Il semble que le facteur I peut être considéré comme une dimension essentielle et individualisable de la dépression.

***Le facteur II Autocritique*** (dépression d'intériorisation) comprend des questions portant sur les sentiments de honte et de culpabilité, les sentiments de vide, d'insécurité, d'insatisfaction, de désespoir (hopeless) ; les impressions d'échec par rapport aux espérances personnelles ou aux stéréotypes sociaux, les sentiments d'incapacité à assumer les blâmes ou les critiques, les difficultés à gérer l'ambivalence (par rapport à soi ou aux autres), à assumer un niveau de responsabilité ou de supporter des menaces de changement (personnels, sociaux...).

***Le facteur III Efficacy*** comprend des items appréciant la confiance en ses ressources et ses capacités, le niveau d'ambition, le sentiment d'indépendance, de force intérieure, de responsabilité, d'orgueil. Ce troisième facteur peut mettre en évidence des défenses hypomaniaques ou un déni des difficultés chez des patients dépendants.

Il faut noter qu'une forme adaptée aux étudiants a été créée par la suite. Le DEQ-A tend à une formulation plus claire des items, des tournures positives remplacent la négation (items 9, 18, 26), et la question des résultats scolaires remplace l'activité professionnelle (item 47).

Nous utiliserons les deux formes dans le cadre de cette recherche, en fonction de l’activité socio-professionnelle des sujets.

#### Le Test de Rorschach

Nous souhaitons ajouter à ces mesures quantitative une mesure qualitative par le test projectif de Rorschach **exclusivement pour les sujets qui accepteront ce test**.

Outre l'inventaire classique des défenses psychiques utilisées par ces patientes, nous individualiserons les indices proposés par JE. EXNER. Cette méthode, largement validée aux Etats-Unis, mais aussi en France, permet de répertorier un large éventail d'indices psychologiques précis, validés statistiquement.

Ce programme à l'avantage de distinguer les constellations psychologiques suivantes: constellation suicidaire, indice de schizophrénie, indice de dépression, déficit de l'adaptation sociale, indice d'hypervigilance, indice d'obsessionnalité.

L'investigation en cours d'évolution peut nous renseigner sur la genèse du trouble, et parait incontournable sur le plan du traitement et du pronostic de ces patientes. Pour ce faire, nous avons besoin du logiciel de traitement et d'analyse EXNER.

### Antécédents de maladies psychosomatiques et d’alexithymie

Nous nous proposons d’investiguer les antécédents de maladies à composante psychosomatique dans l’anamnèse personnelles et familiale des adolescentes présentant des troubles des conduites alimentaires et de les mettre en relation avec l’alexithymie

#### Recherche des antécédents de maladies psychosomatiques

Nous rechercherons cliniquement chez les patients et leurs parents les maladies psychosomatiques (voir tableau suivant).

- Cancer
- Leucémie
- Infarctus
- Angine de poitrine
- Diabète sucré (non insulino-dépendant)
- Diabète sucré (insulino-dépendant)
- Epilepsie
- Ulcères gastro-duodénaux
- Réctolite ulcéro-hémorragique
- HTA essentielle
- Arthrite rhumatoïde
- Asthme
- Migraine
- Psoriasis
- Eczéma
- Urticaire allergique
- Anorexie du nourrisson
- Colique idiopathique
- Régurgitation - Méricysme
- Pelade
- Retard de croissance psychogène
- Spasme du sanglot

#### Echelle d'Alexithymie de Toronto (TAS-20)

Toronto Alexithymia Scale, (Bagby et al, 1992). Traduction et validation G. Loas, M.P. Marchand, (1994).

La T.A.S. 20 est un questionnaire comprenant 20 items cotés de 1 à 5 provenant d'une liste de 41 items mis au point par Bagby et al. en 1992 à partir des 5 composantes de l'alexithymie : difficultés à décrire ses sentiments, difficultés à faire la distinction entre ses sentiments et ses sensations corporelles, manque d'introspection, conformisme social, pauvreté de la vie fantasmatique et peu de souvenirs des rêves. Après sélection le nombre d'items a été ramené à 20 items se répartissant en 4 composantes selon les résultats des analyses en composantes principales, suivies de rotation varimax pratiquées sur les matrices de corrélation des 26 items. Ces composantes sont la capacité à décrire ses sentiments et à pouvoir les distinguer des sensations corporelles (I), la capacité à communiquer ses sentiments à autrui (II), la rêverie diurne (III) et les pensées tournées vers l'extérieur plutôt que vers les sensations intérieures (IV). Le score total est obtenu par sommation des items après recodage pour certains d'entre eux.

La traduction, l'adaptation et la validation de la TAS‑20 en français ont été réalisées par Loas et collaborateurs (1995). Dans cette version française, la TAS‑20 montre une cohérence interne satisfaisante (coefficient alpha de Cronbach supérieur à 0,7) et supérieure à celle de la TAS.

Par contre, contrairement à la version canadienne qui comporte une structure à trois facteurs, la version française n'en retrouve que deux. Dans l'étude de Loas (1995) les facteurs 1 et 2 sont confondus pour ne faire qu'un seul facteur correspondant à la difficulté de reconnaître et de décrire ses sentiments. Le second facteur correspond au troisième facteur de Taylor (pensées tournées vers l'extérieur). Ainsi, les auteurs recommandent de n'utiliser que le score total de cette échelle et non les scores factoriels.

La TAS‑20 contient 20 items dont 14 sont communs à la TAS mais contrairement à cette dernière, il n'existe pas d'items évaluant la rêverie diurne. La TAS représente une approche plus multidimensionnelle de l'alexithymie que la TAS‑20.

Les notes seuils, variant selon la culture d'appartenance de la population, ont été déterminées par Loas (1995) pour la version française. Les notes seuils de la TAS 20 (56 et 44) diffèrent de la version canadienne (61 et 51).

- Score TAS > 56: fonctionnement alexithymique.
- Score < 56 et > 44: indéterminé.
- Score < 44: non alexithymique.

### Les relations familiales :

#### Family Adaptability and Cohesion Scale (FACES III)

La FACES III est un autoquestionnaire standardisé, validé en français (Tubiana, Ruffi et al. 1991) permettant d’évaluer le fonctionnement familial au travers de deux dimensions que sont : la **cohésion et l’adaptabilité familiale**. La **cohésion** familiale est une notion exprimant les liens les **attaches émotionnelles et les sentiments** entre les différents membres d’une famille ainsi que le **degré d’autonomie** au sein de la famille. Les auteurs ont **identifiés quatre niveaux** de cohésion familiale croissant : **familles éclatées, séparées, unies, fusionnelles**. L’**adaptabilité** exprime la possibilité d'une famille à **modifier ses règles de vie** (discipline, rapports d'autorité) en situation particulière (stress, maladie, mort). Elle a été répartie en **4 niveaux, d'intensité croissante d'adaptabilité : les familles rigides, structurées, souples et chaotiques**. Cet auto‑questionnaire court comprend deux parties de 20 items chacune. La première partie de l'échelle FACES III, décrit la famille telle qu'elle est perçue actuellement et la deuxième partie décrit la famille idéale. Pour chaque affirmation, une réponse chiffrée doit être indiquée de 1 (presque jamais) à 5 (presque toujours).

Le développement de FACES III a été l'objet d'une méthodologie rigoureuse. L'échelle est validée, fiable et largement utilisée depuis 81 dans le domaine médical et psychosocial.

Pour la version française la validité structurelle et la validité perçue par les sujets sont excellentes, ainsi que la fidélité. Le coefficient alpha de Cronbach de l'échelle dans sa totalité est de 0,82 (famille perçue et famille idéale), versus 0,68 dans la version originale d'Olson. Ce coefficient est de 0,79 dans la sous-échelle de cohésion (versus 0,77 dans la version originale) et de 0,72 dans la sous‑échelle d'adaptabilité (versus 0,62 dans la version originale) du questionnaire sur la famille perçue. L'étude des coefficients alpha de l'échelle sur la famille idéale indique des valeurs respectives de 0,82, 0,86 et 0,83 pour les scores totaux, de cohésion et d'adaptabilité. Aucun item n'a dû être supprimé pour améliorer ce coefficient.

La cohérence interne du questionnaire dans ses deux parties « perçue » et « idéale » et dans sa totalité est satisfaisante, comme en témoignent les taux élevés de coefficients alpha de Cronbach. La version française de FACES III présente donc une bonne fiabilité, d'un niveau équivalent à l'échelle originale d'Olson, tant pour l'échelle dans son ensemble que pour les sous‑échelles de cohésion et d'adaptabilité.

#### Le Parental Bounding Instrument (PBI)

**Le Parental Bounding Instrument (PBI)** a été construit par Parker et al. (1979-1983). Cet instrument a été utilisé dans différents pays tels l’Angleterre (Gamsaa, 1987), l’Australie (Russell et al. J.D., 1992), l’Espagne (Gomez-Beneyto et al, 1993), le Japon (Katimura et al., 1993), les Etats-Unis (Murphy et al., 1997) etc...

**Cet instrument a été construit sur l’hypothèse que les expériences relationnelle de la petite enfance, principalement les relations avec les parents et leur protection, contribuent à l’étiologie de troubles mentaux à l’âge adulte** (Roe et Siegelman, 1963, Schaefer, 1965, Raskin et al. 1971). **Il est clairement établit dans la littérature (Parker 1979), que pour que les enfant aient un bon développement psychoaffectif, il faut que les liens qu’ils ont avec leurs parents bons** C’est à dire qu’il leur faut une relation d’amour avec leurs parents qui conduit à un attachement sans faille et qui donne des stimulations adéquates au développement.

Cet instrument, mesure, sur une échelle, le souvenir qu’a le patient de sa avec ses parents, père et mère, durant les 16 premières années de sa vie : il évalue la qualité des liens qu’avait le patient avec ses parents durant son enfance Il mesure deux dimensions que sont l’attention et de soins et la sur-protection pour chaque parent. Les échelles d’attention et de soins et de surprotection peuvent être utilisées séparément ou combinées.

Le PBI contient 25 questions, c’est un auto-questionnaire.. Le questionnaire est une liste d’attitude variée et de comportement des parents. On demande au patients de le remplir en cochant ce qui leur rappelle leur père ou leur mère dans leur 16 premières années. On leur demande de choisir, pour chacune des 25 questions, ce qui leur semble être l’appréciation la plus proche de la réalité entre 4 modalités pour évaluer les liens parentaux d’un optimum à des liens perturbés: tout à fait, moyennement, pas tout à fait ou pas du tout. Les mesures sur cette échelles sont indépendantes du sexe des parents et les mères prennent plus soin des enfants et sont plus surprotectrices que les pères aux yeux des patients, mais que cette dimension n’est pas influencée par le sexe de l’enfant.

La surprotection est généralement liée à un manque de soins. Parker (1979) a trouvé que la surprotection est liée avec des scores faibles de soins et que les liens de bonne qualité sont définis par la combinaison de soins importants et de faible hyperprotection.

Une association entre différentes pathologies et les scores de surprotection été montrée comme pour la schizophrénie (Parker et al. 1982), la dépression névrotique (Parker 1979 A), l’anxiété (Parker, 1980), les états phobiques (Parker 1979 B), le transsexualisme (Parker et Barr, 1982), l’asthme (Parker et Lipscombe, 1979 A) et l’hypochondrie (Baker et Merskey, 1982).

# Gestion des données et statistique :

## 9.1 Stratégie d’analyse des données collectées

Les analyses seront réalisées après six mois de suivi des 32 sujets et familles pour l’étude préliminaire (soit 18 mois après le début) afin d’utiliser les résultats pour affiner la méthodologie de l’étude . Et ensuite les analyses seront réalisées tous les six mois jusqu’à la fin .

Nous donnerons une description détaillée de nos échantillons. Les sujets ayant refusés de participer et les non compliants seront comparés au reste de l’échantillon.

La comparaison de l’efficacité des deux protocoles thérapeutiques proposés se fera en comparant les deux groupes de sujets définis sur les variables considérées (cf supra).

## Justification des tests statistiques

Dans un premier temps, nous ferons des tests univariés pour déterminer les facteurs de pronostic autre que la thérapie familiale (Test du Chi deux, test t de, test de Mann-Whitney) en fonction de la nature qualitative ou quantitative, gaussiennes ou non, des variables.

Puis dans un second temps, nous considérerons les variables pour lesquelles les tests univariés sont significatifs et la thérapie familiale dans une analyse multivariée (régression logistique ou régression multiple).

Enfin nous tenterons de déterminer si les sujets ayant initialement des facteurs de mauvais pronostic ont un devenir différent entre les deux groupes.

Et enfin nous tenterons de déterminer sur le groupe de sujets traités par thérapie familiale si des facteurs de mauvaise compliance peuvent être individualisés et quelles sont les facteurs prédisant une meilleure issue.

## Responsable de l’analyse des données (déclaration CNIL si nécessaire) et logiciels de travail

Le conseil statistique sera assure par Fernando Perez-Diaz Statisticien au CNRS URA 1957 hôpital de la Pitié Salpétrière.L’analyse des données sera réalisée grâce au logiciel SPSS.

Nous comptons demander l’accord du CCPPRB de Paris-Cochin et de la CNIL.

# Effets graves éventuels :

Aucun effet grave n’a été constaté.

# Aspects légaux et éthiques

## Obtention des Consentements des sujets inclus :

***.***

L’investigateur remet au sujet et à sa famille une fiche d’information explicative sur l’étude, une information orale de l’étude et de ses modalités leur est donnée. Un consentement écrit est demandé au sujet désirant participer à l’étude initiale, un autre accord pour le suivi lui est également proposé.

- La fiche d’information ainsi qu’un double des consentements sont conservés par le sujet et sa famille.

Les sujets mineurs ne peuvent participer à la recherche qu’après contact des parents, réception de leur accord et signatures des consentements.

***Attribution des fiches d’information et des consentements à chacun des sujets du projet d’étude***

***Apport de la thérapie familiale au traitement des patientes anorexiques***

|  | Fiche Information  Patiente  et famille  (1)* | Consentement  Patiente  Majeure  (2) | Consentement  Patiente  Mineure  (signature des parents)  (3) | Consentement  Famille  (4) | Consentement  Fratrie  Mineure  (signature des parents)  (5) | Consentement  Suivi  Patient Majeur  (6) | Consentement  Suivi  Patiente Mineure  (signature des parents)  (7) |
| --- | --- | --- | --- | --- | --- | --- | --- |
| Patiente Majeure | X | X |  |  |  | X |  |
| Patiente Mineure | X |  | X |  |  |  | X |
| Fratrie Majeure | X |  |  | X |  | X |  |
| Fratrie Mineure | X |  |  |  | X |  | X |
| Parent(s) | X |  |  | X |  | X |  |

* Les fiches d’informations sont déposées au secrétariat.

Les fiches de consentement sont à distribuer **par l’investigateur.**

Elles sont à retirer au bureau recherche.

## Note d’information aux sujets et formulaire de consentement adaptés à chaque groupe

***FICHE D'INFORMATION DU PATIENT***

***ET DE LA FAMILLE***

Nous sollicitons votre concours pour une étude actuellement en cours portant sur l’évaluation des modalités de traitement dans l’anorexie mentale.

Ce projet est placé sous la direction de l'équipe du Professeur Ph. JEAMMET (I.M.M.), 42 bd Jourdan, 75014 Paris, en collaboration avec l’URA 1957 du CNRS et l’unité INSERM 302.

Votre participation à cette étude dépendant de votre accord, nous vous demanderons d'accorder un entretien au médecin ou au psychologue chargé de l'étude. Si vous l’acceptez nous vous demanderons également de remplir seul un questionnaire sur ce sujet. Le traitement donnera également lieu à une évaluation et un test projectif ( ce dernier étant facultatif).

Nous souhaitons comparer deux protocoles de traitement dans l’anorexie mentale, dont l’attribution est aléatoire, en fonction de la présence ou non d’une thérapie familiale.

Les réponses aux questionnaires seront traitées selon des modalités de nature à garantir une stricte confidentialité, ces informations seront soumises comme il se doit au secret médical.

Ces informations seront retranscrites dans un dossier codé strictement confidentiel qui servira à constituer un fichier sur ordinateur de tous les sujets examinés à l'occasion de cette étude. La constitution d'un tel fichier a été soumise à l'approbation de la Commission Nationale de l'Informatique et des Libertés (CNIL), et du Comité Consultatif de Protection des Personnes dans la Recherche Biomédicale (CCPPRB de Paris-Cochin).

Les informations recueillies ne seront consultées que par des professionnels de la santé participant au projet, et ne seront transmises en aucun cas à d'autres personnes (proches, médecin...).

Nous nous intéressons aussi à l’évolution de ces traits psychologiques. C'est pour cela que nous vous demandons, si vous êtes d'accord, l'autorisation de reprendre contact avec vous dans 6,12 et 18 mois et éventuellement ultérieurement, et ce indépendamment du traitement que vous pouvez entreprendre, poursuivre ou interrompre, dans notre service ou dans un autre lieu, pendant la période intermédiaire.

Le refus de ce suivi ne vous empêche pas de participer aux évaluations initiales.

Pour tous renseignements concernant cette étude, vous pouvez contacter :

le Docteur _________________________ Tél. : __________________

**Merci de votre coopération.**

***CONSENTEMENT***

***FAMILLE***

Je soussignée,

**déclare accepter de participer à une étude portant sur l’évaluation des modalités de traitement dans l’anorexie mentale.**

Mon consentement ne décharge pas les organisateurs de la recherche de leurs responsabilités. Je conserve tous mes droits garantis par la loi.

Le promoteur de l'étude a souscrit à une assurance conformément à la Loi Huriet

(art. L 209-7 du CSP du 20/12/1988 et art. 5 du 25/07/1994).

Ce projet est placé sous la direction de l'équipe du Professeur Ph. JEAMMET (I.M.M.), 42 bd Jourdan, 75014 Paris, en collaboration avec l’URA 1957 du CNRS.

Les différentes modalités de cette étude (objectifs, méthodes) m'ont été clairement expliquées.

J'ai eu la possibilité de consulter la fiche d'information préparée à cet effet et de poser à son sujet toutes les questions complémentaires, et j'ai obtenu des réponses claires qui m'ont satisfaite.

J'ai le droit de consulter l'ensemble des informations me concernant en en faisant la demande auprès du centre coordinateur de l'étude.

Un double de ce formulaire m'a été remis pour que je puisse le conserver.

Je suis libre d'arrêter l'étude à tout moment si je le désire.

Nom : Prénom :

Fait à : le :

Signature : Signature du responsable :

***CONSENTEMENT***

***ENFANT(S) MINEUR(S)***

Je soussigné(e),

**déclare accepter que mon enfant participe à une étude portant sur l’évaluation des modalités de traitement dans l’anorexie mentale.**

Mon consentement ne décharge pas les organisateurs de la recherche de leurs responsabilités. Je conserve tous mes droits garantis par la loi.

Le promoteur de l'étude a souscrit à une assurance conformément à la Loi Huriet

(art. L 209-7 du CSP du 20/12/1988 et art. 5 du 25/07/1994).

Ce projet est placé sous la direction de l'équipe du Professeur Ph. JEAMMET (I.M.M.), 42 bd Jourdan, 75014 Paris, en collaboration avec l’URA 1957 du CNRS.

Les différentes modalités de cette étude (objectifs, méthodes) m'ont été clairement expliquées.

J'ai eu la possibilité de consulter la fiche d'information préparée à cet effet et de poser à son sujet toutes les questions complémentaires, et j'ai obtenu des réponses claires qui m'ont satisfait(e).

J'ai le droit de consulter l'ensemble des informations concernant mon enfant, en en faisant la demande auprès du centre coordinateur de l'étude.

Je suis libre d'arrêter l'étude à tout moment si mon enfant ou si moi-même le désirons.

Un double de ce formulaire m'a été remis pour que je puisse le conserver.

Nom, prénom de l'enfant : __________________________ âge : ____

Nom, prénom de l'enfant : __________________________ âge : ____

Nom, prénom de l'enfant : __________________________ âge : ____

Nom, prénom de l'enfant : __________________________ âge : ____

Nom, prénom du parent : ___________________________________

Fait à : le :

Signature : Signature du responsable :

***FORMULAIRE DE CONSENTEMENT***

Je soussigné(e),

**déclare accepter de participer à une étude portant sur l’évaluation des modalités de traitement dans l’anorexie mentale.**

Mon consentement ne décharge pas les organisateurs de la recherche de leurs responsabilités. Je conserve tous mes droits garantis par la loi.

Le promoteur de l'étude a souscrit à une assurance conformément à la Loi Huriet

(art. L 209-7 du CSP du 20/12/1988 et art. 5 du 25/07/1994).

Ce projet est placé sous la direction de l'équipe du Professeur Ph. JEAMMET (I.M.M.), 42 bd Jourdan, 75014 Paris, en collaboration avec l’URA 1957 du CNRS et l’Unité INSERM 302 .

Les différentes modalités de cette étude (objectifs et méthodes) m'ont été clairement expliquées.

J'ai eu la possibilité de consulter la fiche d'information préparée à cet effet et de poser à son sujet toutes les questions complémentaires, et j'ai obtenu des réponses claires qui m'ont satisfait(e).

J'ai le droit de consulter l'ensemble des informations me concernant, en en faisant la demande auprès du centre coordinateur de l'étude.

Je suis également d'accord pour donner des informations concernant mes origines.

Un double de ce formulaire m'a été remis pour que je puisse le conserver.

Je suis libre d'arrêter l'étude à tout moment si je le désire.

Ma participation n'affectera en aucune façon les soins médicaux que je recevrai.

Nom : Prénom :

Fait à : le :

Signature : Signature du responsable :

***FORMULAIRE DE CONSENTEMENT***

***POUR UN ENFANT MINEUR***

Je soussigné(e),

**déclare accepter que mon enfant participe à une étude portant sur les modalités de traitement dans l’anorexie mentale.**

Mon consentement ne décharge pas les organisateurs de la recherche de leurs responsabilités. Je conserve tous mes droits garantis par la loi.

Le promoteur de l'étude a souscrit à une assurance conformément à la Loi Huriet

(art. L 209-7 du CSP du 20/12/1988 et art. 5 du 25/07/1994).

Ce projet est placé sous la direction de l'équipe du Professeur Ph. JEAMMET (I.M.M.), 42 bd Jourdan, 75014 Paris, en collaboration avec l’URA 1957 du CNRS et l’Unité INSERM 302.

Les différentes modalités de cette étude (objectifs et méthodes) m'ont été clairement expliquées.

J'ai eu la possibilité de consulter la fiche d'information préparée à cet effet et de poser à son sujet toutes les questions complémentaires, et j'ai obtenu des réponses claires qui m'ont satisfait(e).

J'ai le droit de consulter l'ensemble des informations me concernant, en en faisant la demande auprès du centre coordinateur de l'étude.

Je suis également d'accord pour donner des informations concernant nos origines.

Un double de ce formulaire m'a été remis pour que je puisse le conserver.

Je suis libre d'arrêter l'étude à tout moment si mon enfant ou si moi-même le désiront.

Cette participation n'affectera en aucune façon les soins médicaux qu'il recevra.

Nom, prénom de l'enfant : __________________________ âge : ____

Nom, prénom du parent : ___________________________________

Fait à : le :

Signature : Signature du responsable :

***CONSENTEMENT POUR ACCORD DE SUIVI***

***PATIENT MAJEUR***

J'ai participé à l'étude initiale sur l ’évaluation des modalités de traitement dans l’anorexie mentale.

**J'accepte que les responsables de cette étude reprennent contact avec moi dans 6,12 et 18 mois pour d’autres investigations.**

Je serai libre si je le désire de ne pas participer au suivi de l'étude. Il me suffira de le signifier à ce moment là au responsable.

Un double de ce formulaire m'a été remis pour que je puisse le conserver.

Les renseignements ci-dessous resteront confidentiels, et ne seront en aucun cas mis en rapport avec mes réponses aux questionnaires.

Voici les renseignements nécessaires :

Nom, prénom : ____________________________________________

Adresse : ____________________________________________

____________________________________________

Tél. : __________________________

Fait à : le :

Signature : Signature du responsable :

***CONSENTEMENT POUR ACCORD DE SUIVI***

***PATIENT MINEUR***

Mon enfant a participé à l'étude initiale sur l’évaluation des modalités de traitement dans l’anorexie mentale.

**J'accepte que les responsables de cette étude reprennent contact avec moi dans 6,12,18 mois pour d’autres investigations avec mon enfant.**

Nous seront libres (lui ou moi) de ne pas participer au suivi de l'étude. Il nous suffira de le signifier à ce moment là au responsable.

Un double de ce formulaire m'a été remis pour que je puisse le conserver.

Les renseignements ci-dessous resteront confidentiels, et ne seront en aucun cas mis en rapport avec les réponses aux questionnaires.

Voici les renseignements nécessaires :

Nom, prénom de l'enfant : __________________________________ âge : ____

Nom, prénom du parent : __________________________________

Adresse : ____________________________________________

____________________________________________

Tél. : ____________________________

Fait à : le :

Signature : Signature du responsable :

# Analyse et chiffrage des surcoûts hospitaliers avec leurs modalités de financement

**Remarque :**

**Ce projet vient à la suite d’une étude préliminaire financée par le crc . Nous incluons cette étude préliminaire dans le calcul de coût et nous la déduisons des frais en fin de budget**

## personnel (médical, non médical)

### Surcoût hospitalier lié à l’inclusion

Chaque famille incluable(nombre estimé à 60 environ) sera retenue d’après la grille de sreening remplie par les cliniciens, puis elle sera reçue par l’équipe de recherche pour expliquer l’étude, recueillir le consentement et procéder à l’inclusion. Pour cela sont nécessaires des entretiens médicaux avec un psychiatre expérimenté ayant un coût horaire de 250 francs pour l’hôpital ( chiffre au 15/07/97 donnés par l’administration).soit

**122 entretiens de ½ heure = 15 250 francs.**

**Tableau 1 : Estimation du surcoût lié à l’inclusion**

|  | **Entretien d’inclusion** |
| --- | --- |
| **Nombre de familles** | **122** |
| **Nombre de séances** | **1** |
| **Durée de l’évaluation** | **0 heure 30** |
| **Nombre d’ heures** | **61** |
| **Coût/heure** | **250 Francs** |
| **Coût** | **15250 Francs** |

### Surcoût hospitalier lié à l’évaluation par une psychologue

Les sujets seront évalués individuellement avec le questionnaire établit au cours d’un entretien d’une heure trente, à l’entrée dans l’étude, puis six mois plus tard par une psychologue expérimentée. soit un coût horaire de 200 francs pour l’hôpital ( chiffre au 15/07/97 donnés par l’administration).(Voir tableau 2)

**Tableau 2 : Estimation du surcoût lié à l’évaluation**

Le coût peut en être estimé comme suit :

- 1 heure 30 par évaluation, par personne
- 3 personnes pour 60 familles
- 1 évaluation initiale
- et une évaluation tous les 6 mois
- soit 4 évaluation par personne

**Soit : 1h30 X 4 x 3 x 60 x 200 francs/heure = 216 000 francs**

|  | **Evaluation initiale** | **évaluation à six, douze et dix huit mois** | **Total sur 18 mois** |
| --- | --- | --- | --- |
| **Nombre de sujets** | 3*60=180 | 3*3*60=180 | 4*3*60=720 |
| **Nombre de séances** | 1 | 1 | **4** |
| **Durée de l’évaluation** | 1 heure 30 | 1 heure 30 | **1 heure 30** |
| **Nombre d’ heures** | 270 | 810 | **144** |
| **Coût/heure** | 200 Francs | 200 Francs | **200 Francs** |
| **Coût** | 54 000 Francs | 162 000 Francs | **216 000Francs** |

### Surcoût hospitalier lié à la thérapie familiale

- Nous comptons inclure 60 familles, soit 30 en thérapie familiale.
- Chaque famille sera vue toutes les 3 à 4 semaines pendant 18 mois soit 18 séances. (voir tableau 3).

**Tableau 3 : Estimation du surcoût lié à la thérapie familiale**

|  | **Total sur 18 mois** |
| --- | --- |
| **Nombre familles** | **30** |
| **Nombre de séances** | **24** |
| **Durée de la séance** | **1 heure 30** |
| **Nombre d’ heures** | **1080** |
| **Coût/heure** | **450 Francs** |
| **Coût** | **486 000 Francs** |

- Chaque séance est assurée par une psychiatre et une psychologue expérimentée, soit un coût horaire de 450 frs pour l’hôpital ( chiffre au 15/07/97 donné par l’administration).
- Chaque séance nécessite 1h30 de travail soit un coût de 675 frs/séance.

**Le coût total est de 24 x 60 familles X 675 frs = 486 000 francs**

| Prévision du nombre d'heures de psychothérapie nécessaire pour un projet sur trois ans et demi , dont 18 mois de CRC | | | | |  |  |  |  |
| --- | --- | --- | --- | --- | --- | --- | --- | --- |
| **Semaine de l'année** | **Première**  **année** | **Deuxième**  **année** | **Troisième**  **année** | **Quatrième**  **année** |
|  | **Nb Heures TF** | **Nb Heures TF** | **Nb Heures TF** | **Nb Heures TF** |
| 3 | 1,5 | 24 | 34,5 | 12 |
| 6 | 3 | 25,5 | 33 | 10,5 |
| 9 | 4,5 | 27 | 31,5 | 9 |
| 12 | 6 | 28,5 | 30 | 7,5 |
| 15 | 7,5 | 30 | 28,5 | 6 |
| 18 | 9 | 31,5 | 27 | 4,5 |
| 21 | 10,5 | 33 | 25,5 | 3 |
| 24 | 12 | 34,5 | 24 | 1,5 |
| 27 | 13,5 | 36 | 22,5 | 0 |
| 30 | 15 | 36 | 21 | 0 |
| 33 | 16,5 | 36 | 19,5 | 0 |
| 36 | 18 | 36 | 18 | 0 |
| 39 | 19,5 | 36 | 16,5 | 0 |
| 42 | 21 | 36 | 15 | 0 |
| 45 | 22,5 | 36 | 13,5 | 0 |
| 48 | 22,5 | 34,5 | 12 | 0 |
| **total en heures de thérapie** | **180** | **486** | **360** | **54** |
| **total en heures de thérapeutes** | **360** | **972** | **720** | **108** |
| **Nombre familles inclues** | **15** | **30** | **30** | **30** |
| **Nombre de séances** | **16** | **8 à 16** | **1 à 16** | **1 à 8** |
| **Durée de la séance** | **1 heure 30** | **1 heure 30** | **1 heure 30** | **1 heure 30** |
| **Coût/heure** | **450 Francs** | **450 Francs** | **450 Francs** | **450 Francs** |
| **Coût** | **81 000 Francs** | **218 700 Francs** | **162 000 Francs** | **24 300 Francs** |
| **prise en charge CRC de l'AP-HP** | **78 300 Francs** | **18 900 Francs** | **0** | **0** |
| **Demande au PHRC** | **2700 Francs** | **199 800 Francs** | **162 000 Francs** | **24 300 Francs** |

## dépenses générales (missions, fournitures de bureau ...)

### Surcoût lié à l’impression des dossiers.

Selon les données financières de nos précédentes études :

- pour 60 familles x 3 sujets x 4 évaluations = 720 dossiers ;
- l’impression des dossiers est évaluée à **4500 francs.**

### Surcoût lié au traitement des données.

Le codage, et le secrétariat concernant les dossiers sont respectivement évalués au vu du coût des études précédentes à **13 125 Francs**

### Surcoût lié à la saisie des données.

La saisie des dossiers est évaluée au vu du coût des études précédentes à **4000 Francs**

### Surcoût lié à l’ analyse des données.

L’analyse des dossiers est évaluée au vu du coût des études précédentes à **20 000 francs**

### Prévision des dépenses de Mission

Elles se résument à des voyages d’étude pour la supervision du projet par l’équipe anglaise du Maudsley.

8 réunions de 3 personnes sur 48 mois soit :

**3 x 8x 1600 francs = 38 400 francs**

### Surcoût lié à l’acquisition du logiciel Exner

Ce logiciel coûte 921 Francs.

## .Conclusion

On peut estimer le surcoût global de cette étude à **798 196 francs**. Nous avions demandé et obtenu pour l’étude préliminaire  **149 921 Francs**

Nous sollicitons donc le complément : **648 275 Francs**

**Tableau 4 :**

**Récapitulatif : estimation du surcoût global**

| **surcoût en Francs lié à** | **Budget CRC** | **Première**  **année PHRC** | **Deuxième**  **année PHRC** | **Troisième**  **année PHRC** | **Coût**  **Total** | **Coût**  **Total PHRC** |
| --- | --- | --- | --- | --- | --- | --- |
| **l’inclusion** | **5 000** | **6 800** | **3 450** | **0** | **15 250** | **10 250** |
| **l’évaluation** | **28 800** | **62 400** | **62 400** | **62 400** | **216 000** | **187 200** |
| **la thérapie familiale** | **97 200** | **100 000** | **186 000** | **102 800** | **486 000** | **388 800** |
| **impression des dossiers** | **1 200** | **3 300** | **0** | **0** | **4 500** | **3 300** |
| **préparation des dossiers** | **2 500** | **3 000** | **3 000** | **4 625** | **13 125** | **10 625** |
| **saisie des dossiers** | **1 200** | **1 000** | **1 000** | **1 000** | **4 000** | **3 000** |
| **l’analyse des données** | **3 500** | **4 000** | **4 000** | **8 500** | **20 000** | **18 800** |
| **missions** | **9 600** | **9 600** | **9 600** | **9 600** | **38 400** | **28 800** |
| **exner** | **921** | **0** | **0** | **0** | **921** | **0** |
| **Total** | **149 921** | **189 900** | **269 450** | **188 925** | **798 196** | **648 275** |

# Références bibliographiques

1. American Psychiatric Association Diagnostic and Statistical Manuel of Mental Disorders Fourth edition Published by the American Psychiatric Association 1994.
2. American Psychiatric Association DSM-III-R : Manuel diagnostique et statistique des troubles mentaux Ed Masson 1989.
3. Baker, B. & Merskey, H. (1982), Parental representations of hypochondriacal patients from a psychiatric hospital British Journal of Psychiatry, 141, 233-238.
4. Beck A.T., Et Beamesderfer A. Assessment of Depression : The Depression Inventory. Psychological Measurements in Psychopharmacology. Mod. Probl. in Pharmacopsychiatry, 7, 151-159, ed. P. PICHOT, Paris, Karger, Basel, 1974.
5. Beck A.T., Steer R. Et Garbin M. Psychometric properties of the Beck depression inventory : txenty-five years of research. Clinical Psychology Review, 1988, 8, 1, 77-100.
6. Beumont P, Al‑Alami.Touyz S.Relevance of a StandardMeasurement of Undernutritionto the Diagnosis of AnorexiaNervosa: Use of Quetelet's BodyMass Index (BMI) International Journal of Eating Disorders, Vol. 7, No. 3, 399‑404( 1988)
7. Blatt S.J. et coll., Dependency and self-criticism : psychological dimensions of depression, in Journal of consulting and clinical psychology, 1982, Vol. 50, 1, 113-124.
8. Blatt S.J., Blass R., Attachement and separateness : a dialectic model of the products and processes of psychological development, in Psychoanalytical Study of the Child, New York, 1990, 45, 107-127.
9. Blatt S.J., Ford R.Q., Berman W., Cook B., Meyer R., The assessement of therapeutic change in schizophrenic and borderline young adults, in Psychoanalytic psychology, 1988, 5, 127-158.
10. Blatt S.J., Ford R.Q., Berman W., Cook B., Meyer R., The assessement of therapeutic change in schizophrenic and borderline young adulte. in Psychoanalytic psychology, 1988, 5, 127‑158.
11. Blatt S.J., Levels of object representation in anaclitic and introjective depression, in Psychoanalytical Study of the Child, New York, 1974, 24, 107-157.
12. Blatt S.J., Zuroff D., Interpersonal relatedness and self-definition : two prototypes for depression, in Clinical Psychology Review, 1992, vol. 12, 527-562.
13. Blatt S.J., Zuroff D., Psychometric properties of the depressive experiences questionnaire, in Journal of Personality Assessement, 1990, 55, 65-72.
14. Blatt Sj., The differential effect of psychotherapy and psychoanalysis with anaclitic and introjective patients : the Menninger psychotherapy research project revisited, J Am Psychoanal Assoc, 1992, 40 (3) : 691-724.
15. Bourke M.P, Taylor G.J, Parker J.D.P., Bagby M.R (1992) Alexithymia in women with anorexia nervosa, a preliminary investigation. BrJ. Psychiatry, 161, 240‑243.
16. Bruch H Anorexia Nervosa: therapy and theory Am J Psychiatry 1982 Dec;139(12):1531-8
17. Bruch H. Les yeux et le ventre (L'obèse, l'anorexique). Paris: Bibliothèque scientifique, Payot, 1978.
18. Caillé P, Abrahamsen P, Girolami , Sörby B Utilisation de la théorie des sytèmes dans le traitement de l'anorexie mentale Évolution Psychiatrique, 1978, 43 (3): 563‑581.
19. Caputo-Sacco L. Et Al., MMPI correlates of Exner’s Egocenticity Index is an adolescent psychiatric population, J Pers Assess, 1991 feb, 56 (1) : 29-34.
20. Cochrane C.E, Brewerton T.D; Wilson D.B, Hodges E.L (1993): alexithymia in the eating disorders, Int. J. Eat. Disorders, 4, 2, 219‑222.
21. Collet L. Et Cottraux J. Inventaire abrégé de la dépression de Beck (13 items). Etude de la validité concurrente avec les échelles de Hamilton et de ralentissement de Widlöcher. L’encéphale, 1986, 12, 77-79.
22. Collet L., Cottraux J. inventaire abrégé de la dépression de Beck (13 items). Etude de la validité concurrente avec les échelles de Hamilton et de ralentissement dépressif de Widlöcher. Encéphale 1986, 12, 77-79.
23. Cottraux J. Depressive cognitions of obsessive-compulsive patients : a factorial analysis of the shorter form of the Beck depression inventory. In perris C et Eisemann M. Cognitive Therapy : an update, Dopuu Press, Umea, 1988.
24. Cottraux J., Bouvard M., Et Legeron P. Méthodes et échelles d’évaluation des comportements. Editions d’applications psychotechniques. Issy les Moulineaux, 286 p., 1985.
25. Crisp AH, Harding B, McGuinness B: Anorexia nervosa: Psychoneurotic characteristics of parents: Relationship to prognosis. J Psychosom Res 18:167 ‑ 173,1974

Crisp AH, Norton K, Gowers S, Halek C, Bowyer C, Yeldham D, Levett G and Bhat A. A controlled study of the effect of therapies aimed at adolescent and amily psychopathology in anorexia nervosa. British Journal of Psychiatry (1991), 159, 325-333

1. Crisp AH: Anorexia Nervosa: Let Me Be, New York, Grune and Stratton, 1980
2. Dally P Treatment of anorexia nervosa. Br J Hosp Med 1981 May;25(5):434, 437-8, 440
3. Danziger Y., Carel C A, Tyano S., Mimouni,A Is Psychotherapy Mandatory During the Acute Refeeding Period in the Treatment of Anorexia Nervosa? Journal Of Adolescent Health Care 1989;10:328 331
4. Dare c, Eisler Y, Cohalan M,Crowther C,Senior R, and Asen E. The listening heart and the chi square: clinical and empirical perceptions in the family therapy of anorexia nervosa. Journal of family therapy (1995) 17: 31-57
5. Dare c,and Eisler Family therapy for anorexia nervosa Journal of family therapy (1997) in Handbook of psychotherapy for anorexia nervosa and bulimia 2nd edition in press
6. Diamond G, . Serrano A, Dickey M, Sonisw Current Status of Family‑Based Outcome and Process Research. J. Am. Acad. Child Adolesc Psychiatry. 35: 1. 6‑16 (1996)
7. Ellul E et Al, Rorschach et dépendance psychique, Psychologie médicale, 1989, 21, 9 : 1291-1294.
8. Epstein NB, Baldwin LM, Bishop DS: The McMaster Family Assessment Device.Journal of Marital and Family Therapy 9:171‑180, 1983
9. Farges F sous la direction de Godart N Etude de l'Alexithymie chez des sujets présentant une Dépendance aux Opiacés Mémoire du DES de Psychiatrie Présenté et soutenu publiquement à Rouen le 14 octobre 1996
10. Flament M Épidémiologie des troubles des conduits alimentaires Épidémiologie Psychiatrique Lépine, Terra et Rouillon(Eds) Paris Ellipse à paraître, 1995.
11. Garfinkel PE, Garner DM, RoseJ, et al A comparison of characteristics in families of patients with anorexia nervosa and normal controls . Psychol Med 13:821828, 1983
12. Garner DM, Garfinkel PE, O'Shaughnessy M The validity of the distinction between bulimia with and without anorexia nervosa. AmJ Psychiatry 142:581587, 1985
13. Gatard M Anorexie mentale : approche familiale Soins Psychiatr 1982 Apr;(18):30
14. Godart N sous la direction du Dr M. Flament Le rôle de la pathologie anxieuse dans le développement des troubles des conduites alimentaires :comorbidité et évolution temporelle de la phobie sociale et des troubles boulimiques et anorexiques. Mémoire pour le D.E.A de Psychopathologie et neurobiologie des comportements Octobre 1994
15. Gomez‑Beneyto M, Pedros A, Tomas A, Aguihr K, and Leal C Psychometric properties of the parental bonding instrument in a spanish sample Soc Psychiatry Psychiatr Epidemiol (1993) 23: 252‑255
16. Gowers S, Norton K, Halek C, Crisp A H Outcome of Outpatient Psychotherapy in aRandom Allocation Treatment Study ofAnorexia Nervosa
17. Guelfi J.D., Dreyfus J.F., Ruschel S., Blanchard C., Pichot P. : Structure factorielle de l’échelle de dépression de Hamilton Ann. Médico-Psychol., 1981, 139, 199-214.
18. Haller E Eating disorders. A review and update. West J Med 1992 Dec;157(6):658-62
19. Halmi KA Eating disorder research in the past decade. Ann N Y Acad Sci 1996 Jun 18;789:67-77
20. Hamilton M A rating Scale for depression . J. Neurol. Neuro-surg. Psychiatry 1960, 23, 56-62.
21. Herzog D B, Keller M B, Lavori P W. Outcome in Anorexia Nervosa and Bulimia Nervosa A review of the litterature Journal of nervous and Mental Disease Vol.176, n°3 1988.
22. Humphrey L L,Chapter 13 Family Relationships in K. H. (ed) Psychobioly and treatement of anorexia nervosa and bulimia nervosa. Washington, D.C. American Psychiatric Press Halmi
23. Jeammet P Anorexies et boulimies Cah. Nutr. Diet., XXIV, 3, 1989a.
24. Jeammet P, Brechon G, Payan C, et al Le devenir de l'anorexie mentale : une étude prospective de 129 patients évalués au moins quatre ans après leur première admission. Psychiatrie de l'enfant, XXXIV, 2, 1991, p. 381 à 442.
25. Jeammet P, Gorgé A.Une forme de thérapie familiale:le groupe de parents Bilan de huit années de fonctionnement d'un groupe ouvert de parents d'anorexiques mentales adolescentes Psychiatrie de l'enfant, XXIII,2,1980
26. Johnson C, Flach A Family characteristics of 105 patients with bulimia . AmJ Psychiatry 142:1321‑1324, 1985
27. Kaganski I et Rémy B. Aspects familiaux, cliniques et thérapeutiques des troubles des conduites alimentaires Confrontations psychiatriques n° 31 ‑ 1989
28. Kalucy R S, Crisp AH, and Harding B. A study with 56 families with anorexia nervosa Br. Med. Psycho. (1977),50 381-395
29. Kitamura T A validation study of the Parental Bonding Instrument in a Japanese population.
30. Kog E, Vandereycken W Family interact on in eating disordered patients and normal controls. Internat onalJournal of Eating Disorders 8:11‑23, 1989
31. La Quatra T.A., Clopton J.R (1994): Characteristics of alexithymia and eating disorders in college women, Addictive behavior, 19, 4, 373‑380.
32. Le Grange PDF., Dare C., Eisler I. Russel G.F.M Evaluation of Family Treatementsin adolescent anorexia nervosa : a Pilot Study Int J Eat Disord 1992 vol 12,n°4 347-357 (1992)
33. Lecrubier Y, Boyer P, Lepine Jp, Weiller E: Paris Centre Report. inMental Illness in General Health Care. An international Study. (eds T.B. Ustun & NSartorius), John Wiley Sons Ltd.,1995: pp. 211‑225.
34. Lecrubier Y, Sheehan DV, Weiller E, Amorim P, Bonara L Sheehan KH, Janavs J, Dunbar GC: The MINI International Neuropsychiatric Interview (MINI) a short diagnostic structured interview: Reliability and validity according to the CIDI. European Psychiatry ; à paraitre
35. Lemperiere T., Lepine J.P., Rouillon F., Hardy P., Ades J., Luaute J.P.; Et Ferrand I. Comparaison de différents instruments d’évaluation de la dépression à l’occasion d’une étude sur l’Athymil 30 mg. Ann. Médico-Psychol., 1984, 142, 1206-1212.
36. Loas G, Fremaux D, Marchand M.P (1995): Etude de la structure factorielle et de la cohérence interne de la version française de l'échelle d'alexithymie de TORONTO à 20 items (TAS 20) chez un groupe de 185 sujets sains, L'Encephale, XXI, 117‑122.
37. Loas G, Fremaux D, Marchand M.P, Chaperot C, Dardennes R (1993): L'alexithymie chez les sujet sain: validation de l'échelle d'alexithymie de TORONTO (TAS) dans une population tout venanb> de 144 sujets, application au calcul de la prévalence, Annales médico‑psychologiques, 151, 96, 660‑663.
38. Loas G, Fremaux D, Otmani O, Verrier A (1995): Prévalence de l'alexithymie en population générale. Etude chez 183 sujets tout venanb> et chez 263 étudiants, Annales médico‑psychologiques, 5, 153, 355‑357.
39. Loas G, Otmani O, Fremaux D, Lecercle C, Duflot M, Delahousse J (1996): Etude de la validité externe, de la fidélité et détermination des notes seuil des échelles d'alexithymie de Toronto (TAS et TAS 20) chez un groupe de malades alcooliques, l'Encephale, XXII; 35‑40.
40. Loas G, Otmani O, Verrier A, Fremaux D, Marchand M.P,: Etude de validation de l'échelle d'alexithymie de Toronto à 20 items. Congrès de psychiatrie et de neurologie de langue française ‑ Toulouse, Masson (Juin 1994).
41. Loas G., Fremaux D., Marchand M.P., Chaperot C., Dardennes R., L'Alexithymie chez le sujet sain validation de l'échelle d'alexithymie de Toranto (TAS) dans une population tout venant de 144 sujets, application au calcul de la prévalence. Communication du 17 mai 1993 à la Société Médico-Psychologique de Paris.
42. Mackinnon AJ. The Parental Bonding Instrument (PBI): an epidemiological study in a general population sample. Psychol Med 19(4), 1023‑1034 (1989)
43. Marocco Muttini C, Minerva Psichiatr, 1993 dec, 34 (4) : 225-229.
44. Martinez J.P. Anorexie, table des jeux et table des lois. In C.E.C.C.O.F.: L'anorexique et sa famille. Paris, 18 octobre 1986. C.E.C.C.O.F.
45. Marty P (1968): La dépression essentielle, Revue française de psychanalyse, 3, 595‑598.
46. Minuchin S, MD; Baker L, RosmanB L; Liebman R; MilmanL;. Todd T C A Conceplual Model of Psychosomatic Illness in Children Family Organization and Family Therapy Arch Gen Paychiatry—Vol 32, Aug 1975
47. Morgan HG; Russell GF Value of family background and clinical features as predictors of long-term outcome in anorexia nervosa: four-year follow-up study of 41 patients. Psychol Med 1975 Nov;5(4):355-71
48. Murphy E. The assessment of parenting using the parental bonding instrument: two or three factors? Psychol Med 27(2), 333‑341 (1997)
49. Mynor-Wallis L M The psychological treatement of eating disorders British Journal of Hospital Medecine, 1989 May,41 470-475
50. Ordman AM, Kirschenbaum DS: Bulimia Assessment of eating, psychological adjustment, and familial characteristics . International Journal of Eating Disorders 5:865‑878, 1986
51. Palazzoli M Self‑Starvation: From Individual to Family Therapy in the Treatment of Anorexia Nervosa . New York, Jason Aronson, 1978
52. Palmer RL, Oppenheimer R, Marshall PD: Eat ng‑disordered patients remember their parents: a study using the parental‑bonding instrument. International Journal of Eating Disorders 7:101‑106, 1988
53. Parker G Factorial validity of the EE scales.Psychol Med 19(2), 435‑446 (1989)
54. Parker G, Tupling H and Brown A Parental Bonding Instrument British Journal of Medical Psychology (1979), 52, 1‑10
55. Parker, G. & Lipscombe, P. (1979a). Parental overprotection and asthma. Journal of Psychosomatic Researd 295-300.
56. Parker, G. & Lipscombe, P. (1979b). Parental characteristics of Jews and Greeks in Australia. Australian an Zealand Journal of Psychiatry, 13, 225-230.
57. Parker, G. & Barr, R. (1982). Parental representations of transsexuals. Archives of Sexual Behaviour, 11, 22.
58. Parker, G. (1979a). Parental characteristics in relation to depressive disorders. Bristish Journal of Psychiatry 138-147.
59. Parker, G. (1979b). Reported parental characteristics of agoraphobics and social phobics. British Journal of Psychiatry. 135. 555-560.
60. Parker, G. (1983). Parental Overprotection. New York : Grune & Stratton.
61. Parker, G., Fairley, M., Greenwood, J., Jurd, S. & Silvoe, D. (1982). Parental representations of schizophrenia and their association with onset and course of schizophrenia. British Journal of Psychiatry, 141, 573-581.
62. Parker, G., Tupling, H. & Browm, L. B. (1979). A Parental Bonding Instrument. British Journal of Medical Psychology, 52, 1-10.
63. Parker. G. (1981). Parental representations of patients with anxiety neurosis. Acta Psychiatrica Scandinavic. 33-66.
64. Patton G C. Mortality in eating disorders Psychological Medecine, 1988, 18, 947-951.
65. Pless l.B., Roghmann K., Haggerty R.J Chronic illness, family functioning and pyschological adjustment: a model for the allocation of preventive mental health service . Int. J. EpidemioL, 1972,1, 271‑9.
66. Robin A. L., Siegel P.T., Koepke T., Moye A, Tice S. Family versus individual therapie for adolescent females with anorexia nervosa Developmental and behavioral pediatrics of eating disorders Vol 15, n°2 ; 111-116 (1994).
67. Robin A. L., Siegel P.T., Moye A. Family versus individual therapie for anorexia : impact on family conflict International journal of eating disorders Vol 17, n°4 ; 313-322 (1995).
68. Russel G.F.M., Dare C., Eisler I Le Grange PDF.(1992) Controlled trials of family treatements in anorexia nervosa and bulimia nervosa in K. H. (ed) Psychobioly and treatement of anorexia nervosa and bulimia nervosa. Washington, D.C. American Psychiatric Press Halmi
69. Russel G.F.M., Szmukler G.I., Dare C., Eisler I.An evaluation of family therapie in anorexia nervosa and bulimia nervosa Arch Gen Psychiatry 1987; 44: 1047-1056.
70. Russell G Évolution et evaluation des traitementsde l'anorexie mentale. Neuropsychiatrie de l'Enfance, 1993, 41 (5‑6), 341‑350.
71. Russell JD, Kopec‑Schrader E, Rey JM, Beumont PJV. The Parental Bonding Instrument in adolescent patients with anorexia nervosa. Acta Psychiatr Scand 1992: 86: 236‑239.
72. Selvini‑Palazzoli M. La famille de l'anorexique et la famille du schizophrène. Une étude transactionnelle Actualités psychiat., 1982, 12 (1): 15‑25.
73. Sheehan DV, Lecrubier Y, Sheehan KH, Janavs J, Weiller E, Keskiner A, Schinka J, Knapp E, Sheehan MF, Dunbar GC: Reliability and validity of the Mini International Neuropsychiatric Interview (MINI) according to the SCID‑P. European Psychiatry; à paraitre
74. Sheehan DV, Lecrubier Y, Sheehan KH, Janavs J, Weiller E: Comparison of the Mini International Neuropsychiatric Interview (MINI) with the SCID‑P and the CIDI: A validity study NCDEU Annnal Meeting May 31‑ June 3, 1995.
75. Sifneos P.E (1973): The prevalence of alexithymic characteristic psychosomatics patients ‑ Psychotherapy and psychosomatics, 22, 255‑262.
76. Sifneos P.E (1988): Alexithymia and its relationship to hemisph specialization, affect and creativity, Psychiatric clinics of North American, 287‑292.
77. Sifneos P.E, Apfeisavitz R, Frankel F.H (1977): The phenom of alexithymia ‑ Observations in psychosomatic patients, Psychotherapy psychosomatics, 28, 47‑57.
78. Skinner HA, Steinhaner PD, Santa‑Barbara J: The Family Assessment Measure. Canadian Journal of Mental Health 2:91‑105, 1983
79. Strober M: The significance of bulimia in juvenile anorexia nervosa: an exploration of possible etiologic factors. International Journal of Eating Disorders 11 1:28‑43, 1981
80. Taylor G.J., Bagby Rm, Parker J.Da, The revised Toronto alexithymia scale Some relinbility, validity and normative data. Psychother Psychosom, 1992, 57, 34-41.
81. Taylor G.J., Bagby RM, Ryan DP Et al., Criteria validity of the Torondo alexithymia scale. Psychosom. Medicine, 1988, 50, 500-509.
82. Taylor, Graeme J., James C.B. Parker M.A., Bagby R.M., A preliminary investigation of alexithymia in men with psychoactive substance dependance. Am. J. Psych. 147-9, sept 1990.
83. Tubiana‑Ruf Ni, L. Moret, K. Bean, M. Mesbah, S. Feard, J.P. Deschame P. Czernichow, A.J. Chwalow Validation en langue française d'une échelle d'évaluation du fonctionnent familial (FACES III): un outil pour la recherche et la pratique clinique Rev. Epidém. et Santé Publ., 1991, 39,531‑541 '
84. Vanderlinden J, Vandereycken W Guidelinefor the Family Therapeutic Approach to Eating Disorders Psychoter Psychosom 1991; 56:36-42
85. WallachJ.D., Lewenkupf El. Five bulimic women. MMPI. Rorschach and TAT characteristics. Int. J. Eating disorder, 1984, 3 53-66.
86. Waller G, Calam R, Slade P Eating disorders and family interaction . BrJ Clin Psychol 28:285‑286, 1989
87. Waller G, Slade P, Calam R Family adaptability and cohesion: relation to eating attitudes and disorders. International Journal of Eating Disorders 9:225‑228, 1990
88. Whipple SB; Manning DE Anorexia nervosa. Commitment to a multifaceted treatment program. Psychother Psychosom 1978;30(3-4):161-9
89. White M Anorexia nervosa: a transgenerational system perspective. Fam Process 1983 Sep;22(3):255-73
90. Yager J Psychosocial treatments for eating disorders. Psychiatry 1994 May;57(2):153-64
91. Yager J Family Issues in the Pathogenesis of AnorexIa Nervosa Psychosomatic Medicine, Vol. 44, No. 1 (March 1982)
92. Yager J The Treatement of Eating Disorders. J Clin Psychiatry 49 ( 9 Suppl) : 18-25, 1988
93. Zgourides G. et al., Anxiety and perceived helplessness as measured by MMPI and Exner-scored Rorschach protocols in a sample of adolescent outpatients, Jpn. J. Psychiatry Neurol. 47(1), 29‑36 (1993)

# Annexes

## Annexe 1

## Annexe 2

**Critères de définition de l’anorexie mentale selon le DSM-IV :**

**Anorexia Nervosa**

**Type restrictif ou avec crises de boulimie 307.1**

**(traduction, M. Flament)**

**A -** Refus de maintenir un poids corporel au niveau ou au-dessus d'un poids minimum normal pour l'âge et la taille ( p.ex perte de poids conduisant au maintien du poids corporel inférieur à 85% du poids attendu, ou incapacité à prendre du poids pendant la période de croissance, conduisant à un poids corporel inférieur à 85% du poids attendu.)

**B -** Peur intense de prendre du poids ou de devenir gros, alors que le poids est inférieur à la normale.

**C -** Altération de la perception du poids ou de la forme de son propre corps, influence excessive du poids ou de l'apparence corporelle sur l'estime de soi, ou déni de la gravité de la maigreur actuelle.

**D -** Chez les femmes post-pubères, aménorrhée, c.-à-d. absence d'au moins trois cycles menstruels consécutifs (une femme est considérée comme aménorrhéique si les règles ne surviennent qu'après administration d'hormones, par exemple d'oestrogènes).

**Type Restrictif (« restricting type ») :** pendant l'épisode actuel d'Anorexie Mentale, le sujet n'a pas, de manière régulière, présenté des crises de boulimie ni recouru aux vomissements provoqués, ou à la prise de purgatifs (c.-à-d. laxatifs, diurétiques, lavements).

**Type avec crises de boulimie / vomissements prise de purgatifs(« binge-eating / purging type »):** pendant l'épisode actuel d'Anorexie Mentale, le sujet a de manière régulière, présenté des crises de boulimie et/ou recours aux vomissements provoqués, ou à la prise de purgatifs (c.-à-d. laxatifs, diurétiques, lavements).

1. ^1^ Chef de clinique assistant dans le « Service de psychiatrie de l’adolescent et du jeune adulte » Institut Mutualiste Montsouris [↑](#footnote-ref-2)
2. ^2^ ^2^ Chargée de recherche à l’INSERM U 302 et attachée dans le « Service de psychiatrie de l’adolescent et du jeune adulte » Institut Mutualiste Montsouris [↑](#footnote-ref-3)
3. ^3^ Assistant dans le « Service de psychiatrie de l’adolescent et du jeune adulte » Institut Mutualiste Montsouris [↑](#footnote-ref-4)
4. ^4^ Assistant dans le « Service de psychiatrie de l’adolescent et du jeune adulte » Institut Mutualiste Montsouris [↑](#footnote-ref-5)
5. ^5^ Assistant thérapeute familial dans le « Service de psychiatrie de l’adolescent et du jeune adulte » Institut Mutualiste Montsouris [↑](#footnote-ref-6)
6. ^6^ Psychologue thérapeute familial dans le « Service de psychiatrie de l’adolescent et du jeune adulte » Institut Mutualiste Montsouris [↑](#footnote-ref-7)
7. ^7^ Chef de Service du « Service de psychiatrie de l’adolescent et du jeune adulte » Institut Mutualiste Montsouris [↑](#footnote-ref-8)
8. ^8^ Statisticien au CNRS URA 1957 [↑](#footnote-ref-9)
9. ^9^ Head of Section « Departement of Psychiatry, Section of Psychotherapy » The Maudsley, Institute of Psychiatry De Crespigny Park Denmark Hill, London SE 8 AF [↑](#footnote-ref-10)
10. ^1 0^ Senior lecturer « Departement of Psychiatry, Section of Psychotherapy » The Maudsley, Institute of Psychiatry De Crespigny Park Denmark Hill, London SE 8 AF [↑](#footnote-ref-11)
